# A Rab/Kinesin-12/kinase module couples vesicle delivery and phragmoplast dynamics during plant cell cytokinesis

**DOI:** 10.1038/s44318-026-00804-1

**Published:** 2026-05-15

**Authors:** Liam Elliott, Moé Yamada, Monika Kalde, Michal Hála, Andrei Smertenko, Frédérique Rozier, James Evry, Niloufer Irani, Yvon Jaillais, Patrick J Hussey, Viktor Žárský, Ian Moore, Roman Pleskot, Charlotte Kirchhelle

**Affiliations:** 1 Laboratoire de Biogenèse Membranaire, Université de Bordeaux, CNRS UMR5200, Villenave d’Ornon, France; 2 Department of Plant Sciences, University of Oxford, Oxford, UK; 3 Laboratoire Reproduction et Développement des Plantes, ENS de Lyon, INRAE, CNRS, UCBL1, Lyon, France; 4 Division of Biological Science, Graduate School of Science, Nagoya University, Nagoya, Japan; 5 Department of Experimental Plant Biology, Faculty of Science, Vinicna 5, Charles University, Prague, Czech Republic; 6 Institute of Experimental Botany, Academy of Sciences of the Czech Republic, Prague, Czech Republic; 7 Institute of Biological Chemistry, Washington State University, Pullman, WA USA; 8 Department of Biosciences, University of Durham, Durham, UK

**Keywords:** Cell Cycle, Membranes & Trafficking, Plant Biology

## Abstract

Cytokinesis is a key process in the development of multicellular organisms, through both formative and proliferative divisions. In land plants, successful cytokinesis requires precise targeting of Golgi-derived vesicles to the future cell division plane by the phragmoplast, a cytoskeletal structure that undergoes continuous remodeling. Endomembrane trafficking and phragmoplast remodeling are tightly coupled, indicating the existence of active crosstalk. However, although many molecular regulators of membrane trafficking and cytoskeletal organization are known, it remains poorly understood how these processes are coordinated. Here, we describe a regulatory module consisting of the membrane-associated GTPase RAB-A2a, cytoskeleton-associated Class II Kinesin-12 proteins, and the Fused kinase ortholog TIO. We provide evidence that the interaction between these molecules at the midzone, and differential functions of Class II Kinesin-12 members at the leading and lagging cell plate domains, are essential for cytokinesis initiation and progression in *Arabidopsis* by simultaneously targeting vesicles to the midzone and coupling phragmoplast remodeling to vesicle delivery.

## Synopsis

Plant cell cytokinesis requires large-scale reorganization of the endomembrane and cytoskeleton systems within a short timeframe. This study provides evidence that a module involving Class II Kinesin-12 proteins, the GTPase RAB-A2a, and the kinase TIO/FUSED functionally couples endomembrane delivery and phragmoplast dynamics during plant cell cytokinesis.

- Arabidopsis Class II Kinesin-12 proteins interact with endomembrane-associated RAB-A2a.
- Class II Kinesin-12 proteins function collectively, but not strictly redundantly, to support membrane targeting and phragmoplast dynamics.
- Class II Kinesin-12 proteins and RAB-A2a also contribute to positioning of TIO/FUSED during cytokinesis.
- TIO/FUSED is required for phragmoplast formation and reorganization during cytokinesis.

## Abstract (web summary)

Class II Kinesin-12 proteins, the GTPase RAB-A2a, and the kinase TIO/FUSED interact to enable phragmoplast remodeling and vesicle delivery to the midzone.

## Graphical abstract

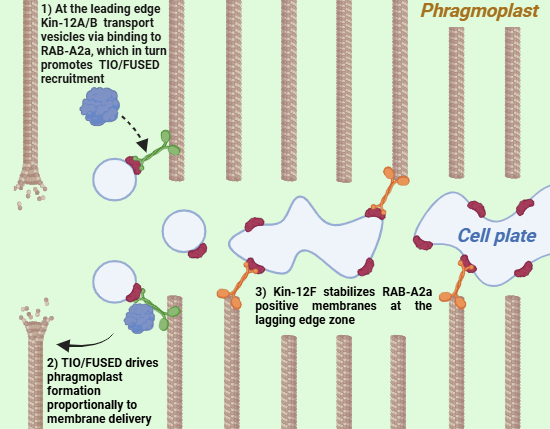

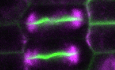

## Introduction

During cytokinesis in plant cells, both the endomembrane and cytoskeleton systems undergo substantial remodeling, which differs markedly from that found in other eukaryotic lineages. Following the dissolution of the spindle at the end of anaphase, plant cells form a phragmoplast, a cytoskeletal structure unique to land plants and some algae. The phragmoplast is predominantly composed of microtubules arranged into two partially-overlapping arrays, with microtubule plus-ends directed towards the future division site at the midzone. Endomembrane vesicles from the *trans*-Golgi network/early endosome (TGN/EE) are mass-transported along phragmoplast microtubules to the midzone where they fuse to form the cell plate, a transient endomembrane compartment that is filled with oligosaccharides. Following the dissolution of the spindle, a brief “phragmoplast initials” phase occurs, where some microtubules persist at the spindle poles while phragmoplast assembly begins at the cell midzone. The phragmoplast initials give rise to a disk phragmoplast, which subsequently transitions into a centrifugally-expanding ring phragmoplast. Phragmoplast expansion is achieved through microtubule depolymerization at the phragmoplast core once the cell plate no longer requires a cytoskeletal scaffold, and simultaneous microtubule polymerization at the phragmoplast outer edge. This remodeling drives centrifugal expansion of the cell plate, until it eventually fuses with the plasma membrane to form a new crosswall (Smertenko et al, 2017).

To ensure successful cytokinesis, the cell plate needs to expand within a 2D plane to form a surface large enough to partition the entire cell (new cross walls are typically several hundred µm^2^). This process depends on the precise control of two factors: (1) spatial targeting of vesicles to the midzone via the phragmoplast, and (2) temporal coordination between membrane delivery and phragmoplast remodeling. Cytoskeleton-associated molecular machinery located at the phragmoplast and cell periphery regulate phragmoplast formation, remodeling and positioning. These include kinesins of the Kinesin-12 family (Lee et al, 2007; Herrmann et al, 2018), IQ67 domain family proteins (Kumari et al, 2021), microtubule crosslinkers of the MICROTUBULE-ASSOCIATED PROTEIN 65 (MAP65) family (Müller et al, 2004; Ho et al, 2011; Kosetsu et al, 2013; Li et al, 2017), and diverse regulators of microtubule nucleation, polymerization, and severing (summarized by Smertenko et al, 2018). Microtubule depolymerization at the phragmoplast core is regulated by at least three signalling pathways involving Mitogen-Activated Protein Kinase (MAPK), Cyclin-dependent kinase (CDK), and Aurora Kinase (Nishihama et al, 2001, 2002; Soyano et al, 2003; Sasabe et al, 2006, 2011; Kosetsu et al, 2010). The widely-conserved signalling kinase TWO-IN-ONE (TIO) has also been linked to initiation of phragmoplast expansion in *Arabidopsis* microspores (Oh et al, 2005), a function that may relate to its interactions with the phragmoplast-associated Kinesin-12 proteins PAKRP1/Kinesin-12A (Kin-12A) and PAKRP1L/Kinesin-12B (Kin-12B) (Oh et al, 2012, 2014). Kin-12A/B localize to interdigitating microtubules at the phragmoplast midzone in somatic cells of *Arabidopsis thaliana* (Lee et al, 2001; Pan et al, 2004), and are redundantly required for proper phragmoplast formation during mitotic cytokinesis of *Arabidopsis* microspores (Lee et al, 2007). In a *kin12a kin12b* (*kin-12a/b*) double null mutant, the majority of *Arabidopsis* microspores fail to form organized phragmoplasts during cytokinesis, causing reduced fertility (Lee et al, 2007). However, somatic cytokinesis has been reported to occur normally in *kin-12a/b* double mutants, indicating cell-type specific functional redundancy (Lee et al, 2007).

Directional transport of TGN/EE membrane vesicles along phragmoplast microtubules to the midzone is believed to be driven by phragmoplast-associated transport kinesins. Such kinesins have not been identified in angiosperms, but recent data indicate that in the moss *Physcomitrium patens*, these include some members of the Kinesin-12 family (Yamada et al, 2025). Vesicle transport to the cell plate also requires regulators of endomembrane trafficking such as Rab GTPases, multi-subunit tethering complexes, dynamins, and SNARE components (Waizenegger et al, 2000; Assaad et al, 2001; Kang et al, 2003; Chow et al, 2008; Fendrych et al, 2010; Zhang et al, 2011; El Kasmi et al, 2013; Rybak et al, 2014; Kirchhelle et al, 2016). Vesicles are delivered to the midzone at the phragmoplast leading edge, before fusing to form a tubulo-vesicular network in the transition zone, and eventually becoming a complete cell plate in the phragmoplast lagging edge. While phragmoplast expansion is essential for spatially directing vesicle transport during cell plate formation, vesicle trafficking from the TGN/EE can conversely influence phragmoplast expansion. Pharmacological inhibition of membrane trafficking from the TGN/EE (Yasuhara and Shibaoka, 2000) as well as mutants in endomembrane regulators (Steiner et al, 2016; Lin et al, 2019) can perturb phragmoplast organization and expansion. This indicates the existence of crosstalk between mechanisms controlling cytoskeleton remodeling and endomembrane trafficking during cytokinesis to ensure coordination of both processes. However, it is not known how information about endomembrane dynamics and activity during cytokinesis is integrated into control of phragmoplast formation and dynamics.

Here, we describe the interaction between the endomembrane-associated small GTPase RAB-A2a, members of the cytoskeleton-associated Kinesin-12 family, and the Fused kinase ortholog TIO during cytokinesis in plant cells. We present evidence that together, they form a core regulatory module that simultaneously contributes to targeting molecular regulators of cytokinesis to the phragmoplast midzone, and couples cytoskeletal remodeling to endomembrane delivery during cell division. We conclude the RAB-A2a/Kin-12/TIO module provides a mechanistic basis for robust spatio-temporal control of plant cytokinesis.

## Results

### RAB-A2a interacts with Class II Kinesin-12s in vitro and *in planta*

**Figure 1:**
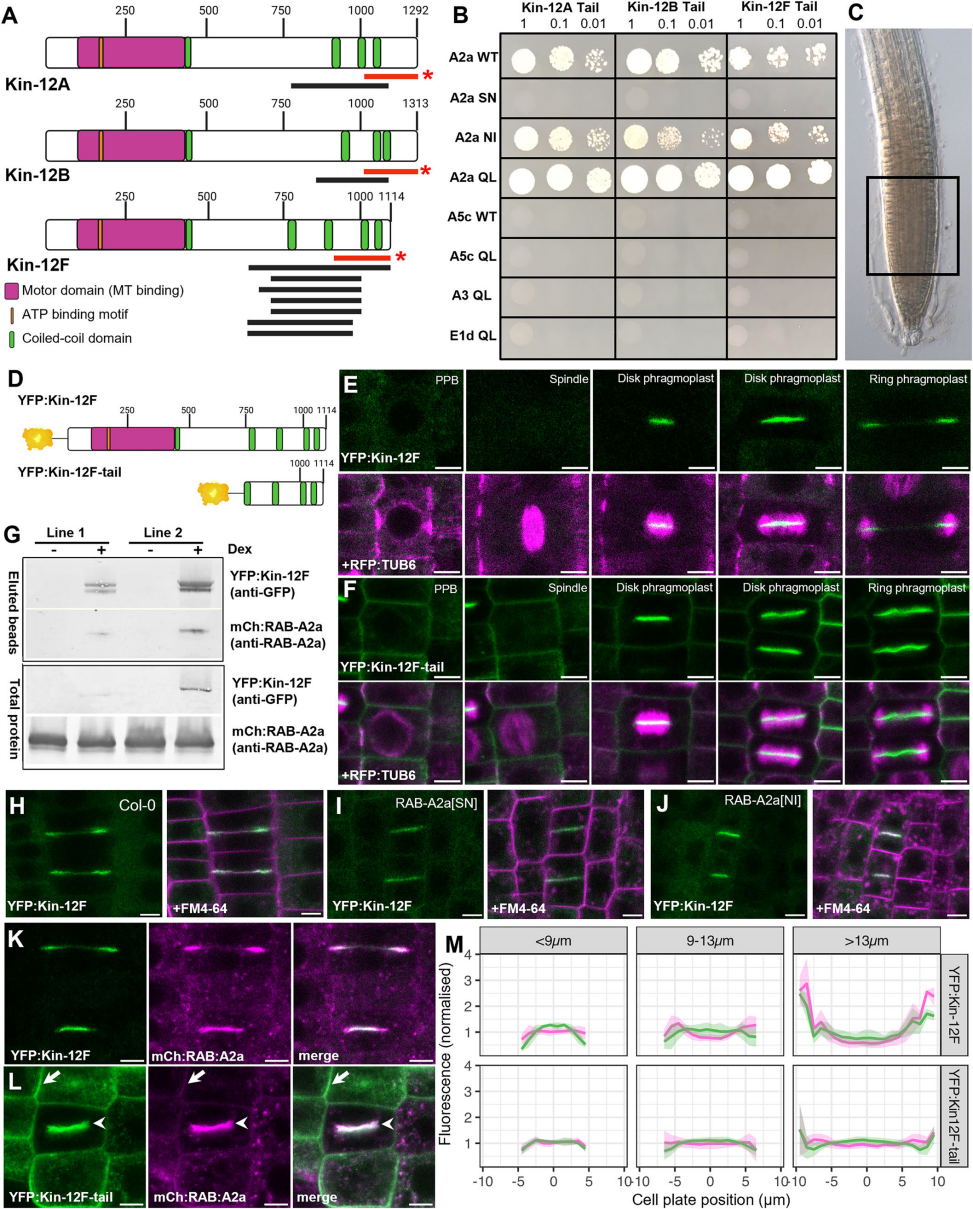
RAB-A2a interacts with Class II Kinesin-12 members in vitro and *in planta.* (**A**) Schematic depiction of Class II Kin-12 proteins. Black lines: clone region isolated as interactors of RAB-A2a in initial Y2H screen. Red lines and asterisks: independently cloned regions used in pairwise Y2H tests in (**B**). Schematic depictions are based on those in Müller and Livanos, 2019 (**B**) Pairwise Y2H tests between Kin-12 tail regions and WT versions/mutant variants of RAB-A2a, RAB-A5c, RAB-A3 and RAB-E1d on SD-Leu-Trp-Ade-His. (**C**) Brightfield image of 4 day old primary root. Confocal images of *Arabidopsis* primary root meristematic epidermal cells in this study were taken from the area indicated by the black box. (**D**) Schematic depiction of YFP:Kin-12F and YFP:Kin-12F-tail. (**E**, **F**) Confocal Laser Scanning Microscopy (CLSM) sections of primary root meristematic epidermal cells (**C**) co-expressing RFP:TUB6 and DEX»YFP:Kin-12F (**E**) or DEX»YFP:Kin-12F-tail (**F**) at different stages of cell division. (**G**) Immunoblots analysed with anti-YFP and anti-RAB-A2a showing co-immunoprecipitation between YFP:Kin-12F and mCh:RAB-A2a in two independent lines co-expressing DEX»YFP:Kin-12F and mCh:RAB-A2a, in presence (+) or absence (−) of Dex to induce expression of YFP:Kin-12F. (**H**–**J**) CLSM section of primary root meristematic epidermal cells expressing DEX»YFP:Kin-12F alone (**H**) or with DEX»RAB-A2a[SN] (**I**) or DEX»RAB-A2a[NI] (**J**) for 16 h, counterstained with FM4-64. (**K**, **L**) CLSM section of primary root meristematic epidermal cells co-expressing *mCh:RAB-A2a* and *DEX»YFP:Kin-12F* (**K**) or *DEX»YFP:Kin-12F-tail* (**L**). White arrow indicates PM, arrowhead indicates cell plate. (**M**) Fluorescence intensity of YFP:Kin-12F (green) or YFP:Kin-12F-tail (green) and mCh:RAB-A2a (magenta) along cell plates as those shown in (**K**, **L**). Cell plates were grouped by diameter into short ( < 9 µm), medium (9–13 µm) and long ( > 13 µm). Lines are mean values, shaded areas are +/− 1 SD. *N* = 4 (YFP:Kin-12F-tail short), 7 (YFP:Kin-12F long), 10 (YFP:Kin-12F short), 11 (YFP:Kin-12F-tail long), 14 (YFP:Kin-12F medium), 16 (YFP:Kin-12F-tail medium). Scale bars, 5 µm. Source data are available online for this figure.

**Figure EV1:**
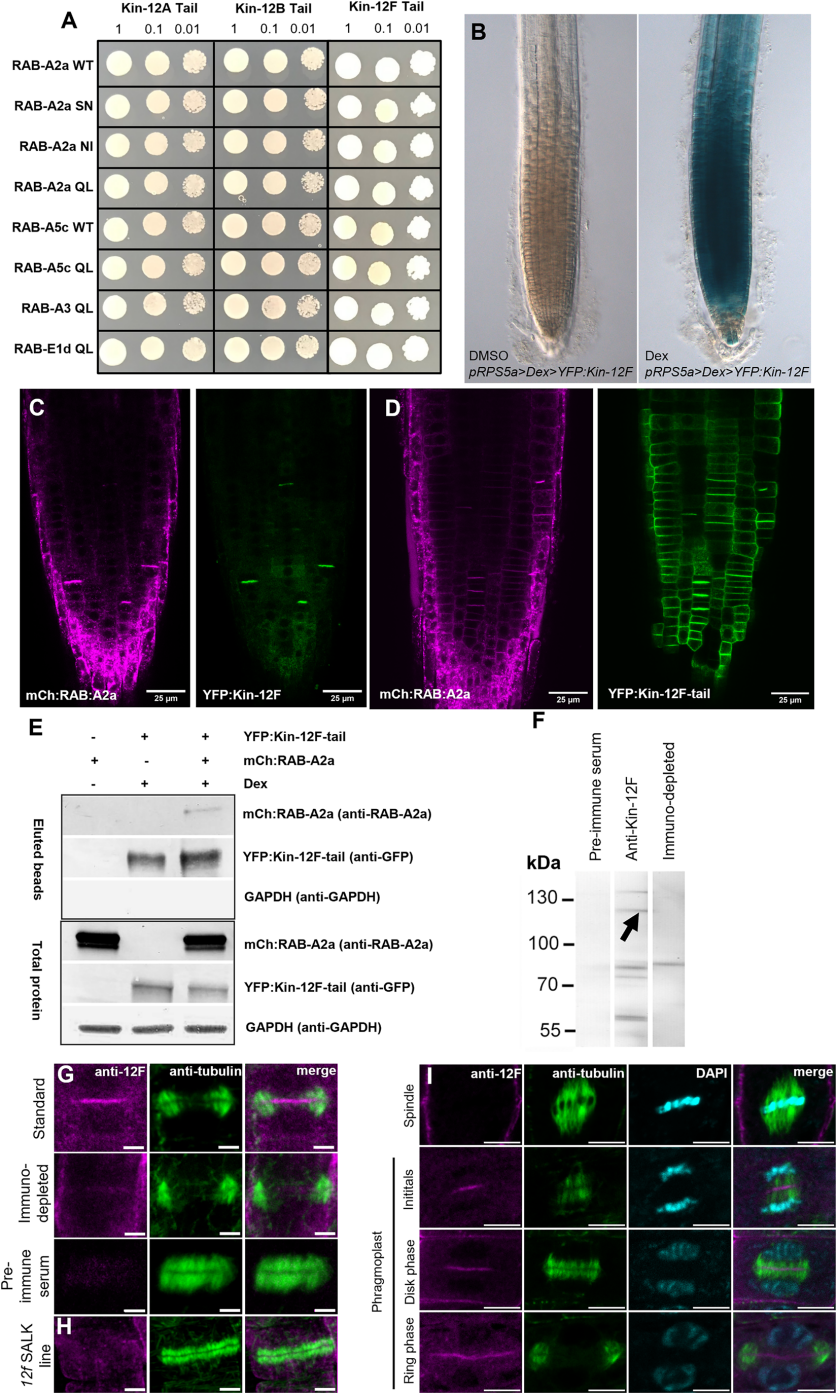
RAB-A2a interacts with Class II kinesin-12 members in vitro and *in planta.* (**A**) Pairwise Y2H tests between Kin-12 tail regions and Rab-A GTPase variants on SD-Leu-Trp. Related to Fig. 1B. (**B**) Brightfield images of GUS-stained DEX» YFP: Kin-12F primary roots after 16 h treatment with either 5 µm Dex or DMSO. (**C**, **D**) CLSM section of primary roots co-expressing mCh: RAB-A2a and DEX»YFP: Kin-12F (**C**) or DEX»YFP: Kin-12F-tail (**D**). (**E**) Immunoblots analysed with anti-YFP, anti-RAB-A2a and anti-GAPDH showing co-immunoprecipitation between YFP: Kin-12F-tail and mCh: RAB-A2a in lines co-expressing DEX»YFP: Kin-12F-tail and mCh: RAB-A2a, in presence (+) or absence (−) of Dex to induce expression of YFP: Kin-12F-tail. (**F**) Immunoblots analyses of *Arabidopsis* seedling protein extract with pre-immune serum, anti-kin12F, and immune-depleted serum. Arrowhead indicates band corresponding to Kin-12F of ~125 kDa. (**G**) CLSM sections of endogenous Kin-12F immunolocalization in primary root meristematic cells using anti-kin12F and anti-tubulin in wild-type plants with standard, immune-depleted and pre-immune conditions. (**H**) CLSM section of endogenous Kinesin-12F immunolocalization using anti-kin12F and anti-tubulin in a *kin-12f* SALK insertion line. (**I**) CLSM sections of endogenous Kin-12F immunolocalization in primary root meristematic cells using anti-kin12F and anti-tubulin in wild-type plants counterstained with DAPI. Scale bars 5 µm (**G**, **H**) and 10 µm (**I**).

In a Y2H screen for interactors of the TGN/EE and cell plate associated GTPase RAB-A2a, we isolated 11 independent clones encoding for the C-terminal (tail) regions of three Kinesin-12 proteins: Kin-12A, Kin-12B and Kin-12F (Fig. 1A). These Kin-12s represent all members of the Kin-12 Class II subgroup (Müller and Livanos, 2019). No clones corresponding to any of the three Class I Kin-12s or any other kinesins were isolated in our Y2H screen. To validate the interaction between RAB-A2a and Class II Kin-12s, we conducted pairwise Y2H tests using independently-cloned constructs of the Kin-12 tail regions. We confirmed Y2H interactions of all Class II Kin-12s with wild-type RAB-A2a as well as with two mutant variants of RAB-A2a: RAB-A2a[QL], a variant with reduced GTPase activity that is considered ‘constitutively active’ (Chow et al, 2008), and RAB-A2a[NI], a variant with reduced nucleotide affinity (Chow et al, 2008) (Figs. 1B and EV1A). By contrast, we found no evidence of a Y2H interaction between Class II Kin-12 tail regions and RAB-A2a[SN], a protein variant predicted to have increased binding affinity for GDP and thus to be arrested upstream of Rab GTPase activation (Chow et al, 2008) (Figs. 1B and EV1A). Class II Kin-12 tail regions likewise did not interact with wild-type/[QL] variants of three other Rab GTPases that localize to the cell plate during cytokinesis in Y2H (Figs. 1B and EV1A). Taken together, these data indicate that Class II Kin-12s specifically interact with RAB-A2a in its active form.

Kin-12A/B localize to the phragmoplast midzone during cytokinesis, where they are thought to play a structural role in phragmoplast organization (Lee et al, 2001, 2007; Pan et al, 2004). To investigate Kin-12F localization, we first attempted to express fluorescently tagged Kin-12F constitutively under the control of the 35S promoter. However, we recovered no stable transgenic lines with either a gDNA or cDNA amplification template. We therefore used the dexamethasone-inducible pOp6/LhGR expression system driven by the *AtRPS5a* promoter (Samalova et al, 2019) to conditionally express YFP:Kin-12F (*AtRPS5a»Dex»YFP:Kin-12F*), as well as a truncated protein variant encompassing the Kin-12F tail including the RAB-A2a interaction domain identified in Y2H, but lacking the Kin-12F motor domain (*AtRPS5a»Dex»YFP:Kin-12F-tail)* in the primary root meristem (Fig. 1C,D).

AtRPS5a-driven pOp6/LhGR was active during all stages of the cell cycle in meristematic root cells (Fig. EV1B), and both YFP:Kin-12F and YFP:Kin-12F-tail were expressed after 24 h Dex induction in primary root tips (Fig. EV1C,D). However, YFP:Kin-12F-tail was detected during all stages of the cell cycle while YFP:Kin-12F was only detectable during cytokinesis (Figs. 1E,F and EV1C,D), suggesting that Kin-12F was suppressed in non-dividing cells through a mechanism involving its N-terminus. During cytokinesis, YFP:Kin-12F localized to the phragmoplast midzone and followed the expanding phragmoplast (Fig. 1E), matching previous reports of Kin-12A/B localization (Lee et al, 2001, 2007). YFP:Kin-12F-tail was also recruited to the midzone during cytokinesis but in contrast to full-length YFP:Kin-12F, persisted at the entire cell plate during ring phase (Fig. 1F). During interphase, YFP:Kin-12F-tail localized to the plasma membrane (PM; Figs. 1F and EV1D). We also performed immunolocalization of endogenous Kin-12F in wild-type *Arabidopsis* primary roots to confirm whether YFP:Kin-12F localization to the midzone was representative of the native protein localization. Anti-Kin-12F (a polyclonal antibody we raised against a 16 amino acid peptide sequence located in the N-terminal region of Kin-12F) was detected at the phragmoplast midzone from the initials phase onwards, similar to the localization pattern of YFP:Kin-12F (Fig. EV1F–I). Additionally, localization of full-length YFP:Kin-12F to the midzone persisted upon expression of the dominant-negative protein variants RAB-A2a[NI] and RAB-A2a[SN] (Fig. 1H–J), suggesting that YFP: Kin-12F recruitment to the midzone does not depend on RAB-A2a function.

**Figure EV2:**
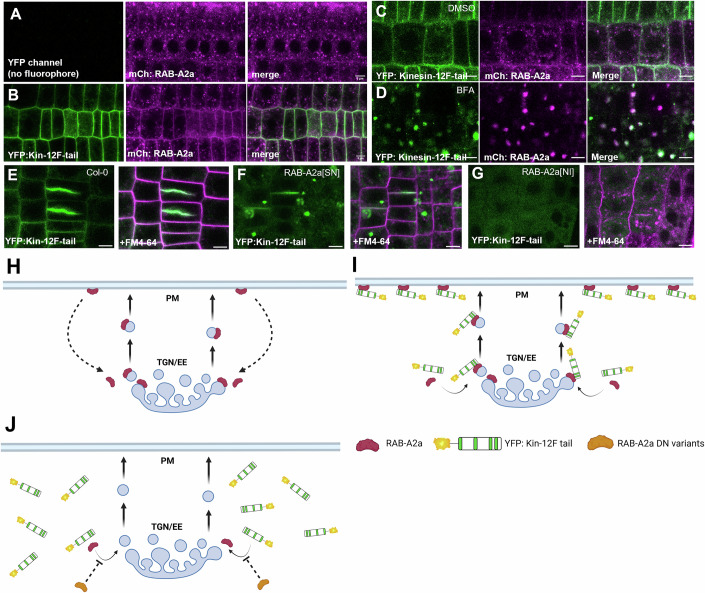
The localization pattern of Kin-12F Tail affects RAB-A2a localization but is also dependent on RAB-A2a activity. (**A**, **B**) CLSM sections of primary root epidermal meristematic cells expressing mCh: RAB-A2a without (**A**) and with (**B**) DEX»YFP: Kin-12F-tail co-expression. (**C**, **D**) CLSM sections of primary root epidermal meristematic cells co-expressing DEX»YFP:Kin-12F-tail and mCh:RAB-A2a upon 30 min treatment with 50 µM BFA (**D**) or equivalent DMSO concentration (**C**). (**E**–**G**) CLSM section of primary root meristematic epidermal cells expressing DEX»YFP:Kin-12F tail alone (**E**) or with DEX»RAB-A2a[SN] (**F**) or DEX»RAB-A2a[NI] (**G**) for 16 h, counterstained with FM4-64. Scale bars, 5 µm. (**H**–**J**) Explanatory schematics of YFP: Kin-12F tail’s effect on RAB-A2a localization. During interphase, RAB-A2a is recruited to the TGN/EE from which it defines a trafficking pathway to the PM, from which it is recycled back to the TGN/EE (**H**). When expressed, YFP: Kin-12F tail associates with RAB-A2a and it transported to the PM. YFP: Kin-12F tail at the PM sequesters RAB-A2a their, either by blocking its removal from the PM or providing a competitive point of re-recruitment to the membrane (**I**). Expression of RAB-A2a dominant-negative variants interferes with membrane recruitment/activation of RAB-A2a, blocking YFP: Kin-12F association with membranes and transport to the PM. YFP: Kin-12F therefore localises to the cytosol (**J**).

We next co-expressed YFP:Kin-12F and YFP:Kin-12F-tail with *p35S::mCherry:RAB-A2a*. YFP:Kin-12F and mCh:RAB-A2a broadly co-localized all along early cell plates, and later at the edges of expanding cell plates (Fig. 1K,L). However, YFP:Kin-12F was consistently depleted relative to mCh:RAB-A2a at the outermost leading edge, which is the major site of vesicle delivery (Fig. 1K,M). By contrast, YFP:Kin-12F-tail was distributed more uniformly across cell plates at all stages (Fig. 1F,L,M). Interestingly, we also noticed a shift in mCh:RAB-A2a localization towards a more uniform pattern across the cell plate when it was co-expressed with YFP:Kin-12F-tail (Fig. 1K–M). mCh:RAB-A2a distribution was also altered in interphase cells in the presence of YFP:Kin-12F-tail, displaying increased recruitment to the PM compared to in the absence of YFP:Kin-12F-tail (Figs. 1L, EV1C,D, and EV2A,B). The PM localization of YFP:Kin-12F-tail was abolished by treatment with the TGN/EE recycling inhibitor Brefeldin-A (Fig. EV2C,D), and by expression of the dominant-negative protein variants RAB-A2a[NI] and RAB-A2a[SN] (Fig. EV2E–G). This indicates that PM localization of YFP:Kin-12F-tail was dependent on RAB-A2a-mediated exocytic transport from the TGN/EE (Chow et al, 2008; Pang et al, 2022) (Fig. EV2H–J).

Finally, we performed co-immunoprecipitation experiments against YFP:Kin-12F/YFP:Kin-12F-tail in the presence of mCh:RAB-A2a. We found that mCh:RAB-A2a co-purified with YFP:Kin12F (Fig. 1G), as well as YFP:Kin-12F-tail (Fig. EV1E), providing further evidence that these proteins can interact *in planta*. Taken together, we conclude that Class II Kin-12 members and RAB-A2a interact *in planta* at the cell plate, and that this interaction can also occur at other subcellular locations during interphase when the RAB-A2a-interaction domain of Class II Kin-12s is expressed ectopically. Class II Kin-12s can therefore be considered to be RAB-A2a effectors.

### Class II Kinesin-12s are required for somatic cytokinesis

**Figure EV3:**
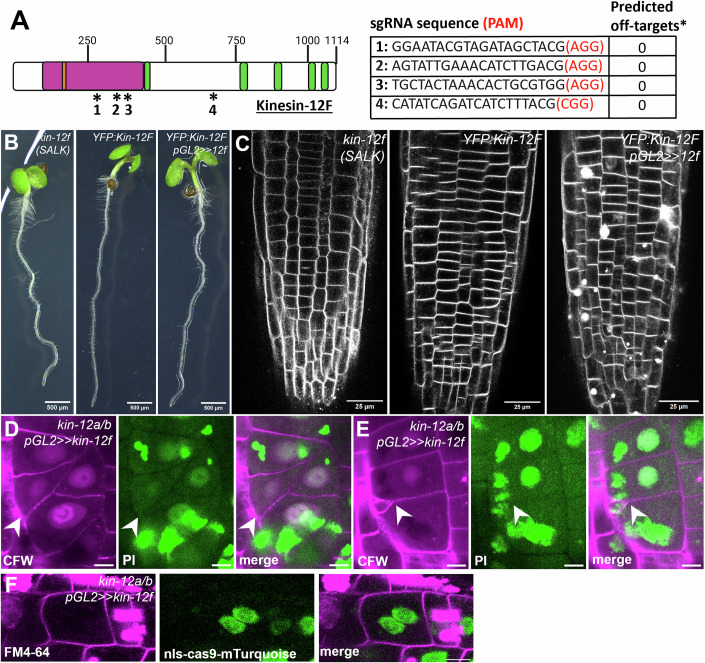
Class II Kin-12 proteins are required for somatic cytokinesis and contribute to midzone membrane targeting. (**A**) Schematic of Kinesin-12F indicating positions of sgRNAs, and table indicating sgRNA sequences with predicted off-targets. sgRNA sequences in black, adjacent PAM site in red. *Off-targets as predicted by CHOPCHOP, which searches for sequences with up to 3 mismatches in the 20 bp sequence upstream of the PAM. 0 off-targets therefore means that no sequence matches were found that have 3 or fewer mismatches with the sgRNA sequence. (**B**) Brightfield images of 5-day-old seedlings of *kin-12f SALK, YFP: Kin-12F*, and *YFP: Kin-12F pGL2»12 f* backgrounds. (**C**) CLSM sections of primary roots of *kin-12f SALK, YFP: Kin-12F*, and *YFP: Kin-12F pGL2»12 f* backgrounds counterstained with FM4-64. (**D**,** E**) CLSM section of *kin-12a/b pGL2»kin-12f* primary root meristematic epidermal cells co-stained with Calcofluor White (CFW) and Propidium Iodide (PI) showing complete but misaligned (**D**) and branched crosswalls (**E**). (**F**) CLSM section of primary roots meristematic epidermal cell in *kin-12a/b pGL2»kin-12f* background counterstained with FM4-64. Note that there is no sign of crosswall formation. Scale bars, 5 µm.

**Figure 2:**
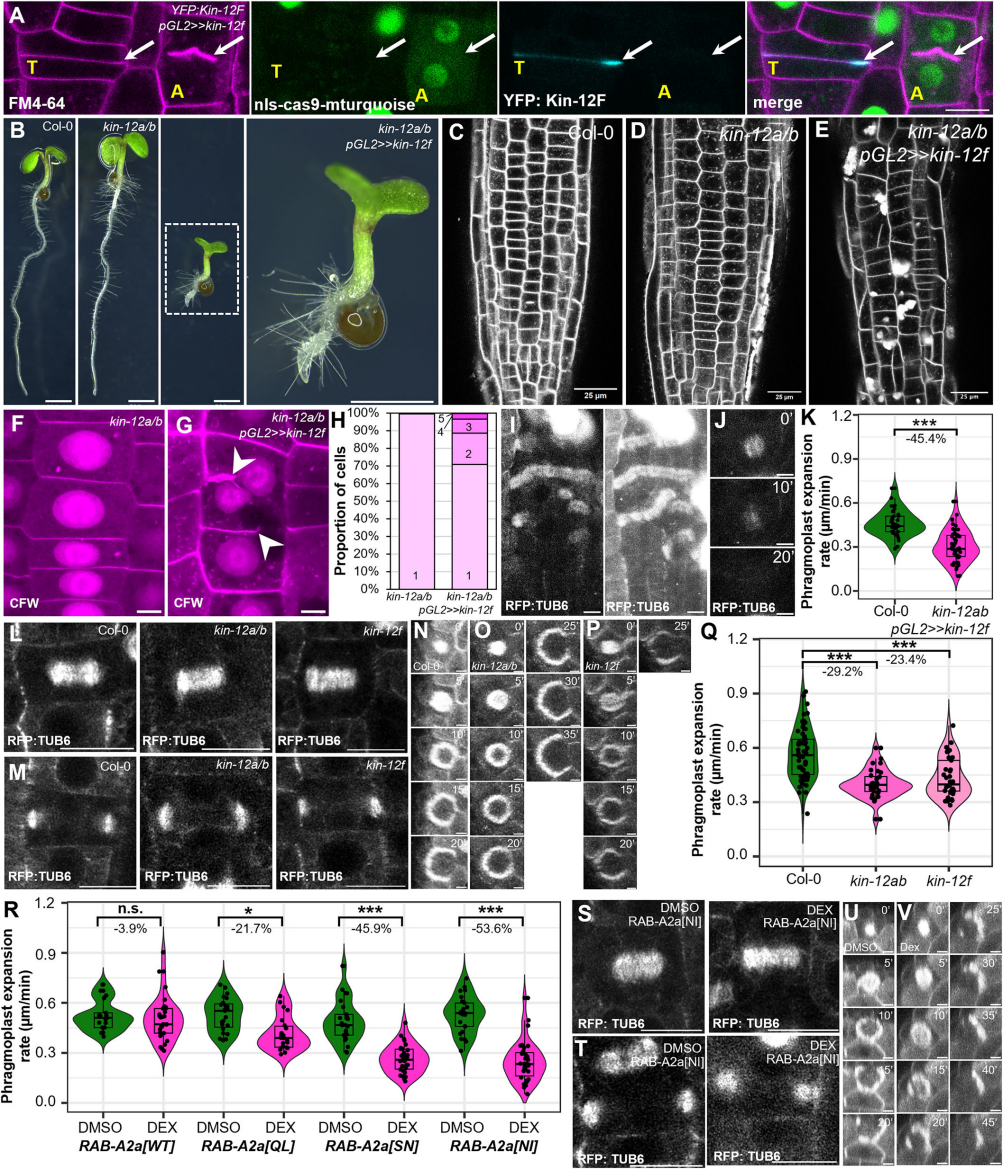
Class II Kinesin-12 members are required for successful somatic cytokinesis and with RAB-A2a are required for phragmoplast expansion. (**A**) CLSM section of primary root meristematic epidermal cells in DEX»YFP:Kin-12F *pGL2::kin-12f::cas9-nls-mturquoise (pGL2»12 f)* background counterstained with FM4-64. White arrows indicate cell plates. A atrichoblast, T trichoblast. Scale bars, 10 µm. (**B**) Brightfield images of Col-0 wild-type, *kin-12a/b* and *kin-12a/b pGL2»kin-12f* 5 day old seedlings. Scale bars, 500 µm. (**C**–**E**) CLSM section of primary roots stained with FM4-64 from plants as those shown in (**B**). (**F**, **G**) CLSM section of primary root meristematic epidermal cells stained with Calcofluor White (CFW) from *kin-12a/b* and *kin-12a/b pGL2»kin-12f* 5 day old seedlings. Arrowheads indicate incomplete crosswalls. Scale bars, 5 µm. (**H**) Quantification of nuclei per cell in primary root meristems of *kin-12a/b* (*n* = 449 cells) and *kin-12a/b pGL2»kin-12f* (*n* = 291 cells) 5 day old seedlings such as those shown in (**B**–**G**). The number of multinucleate cells is significantly increased in *kin-12a/b pGL2»kin-12f* compared to *kin-12a/b* (Chi-squared test, *P* = 1.5501e-297). (**I**) CLSM projections of misaligned phragmoplasts in primary root meristematic epidermal cells in *RFP: TUB6 kin-12a/b pGL2»kin-12f* 4-day-old seedlings. Scale bars, 5 µm. (**J**) CLSM section time series of primary root meristematic epidermal cell in *RFP: TUB6 kin-12a/b pGL2»kin-12f* seedling showing failure to form a phragmoplast after spindle dissolution. Images taken at 5 min intervals but 10 min intervals are shown. Scale bars, 5 µm. (**K**) Violin plots of phragmoplast expansion rates in primary root epidermal meristematic cells in Col-0 (*RFP: TUB6*) and *RFP: TUB6 kin-12a/b pGL2»kin-12f* backgrounds. Expansion rate was significantly reduced in the *RFP: TUB6 kin-12a/b pGL2»kin-12f* background (*P* = 5.9e-11, Student’s *T* test). *N* = 24 phragmoplasts for Col-0, 31 for *kin-12a/b pGL2»kin-12-f*. In box plots, the median is displayed as a line, lower and upper hinges correspond to the 25th and 75th percentiles, the lower and upper whiskers extend from the hinge to the smallest or largest value no further than 1.5 * IQR from the hinge. (**L**, **M**) Airyscan CLSM sections of primary root epidermal meristematic cells expressing *RFP: TUB6* in Col-0, *kin-12a/b* and *kin-12f* backgrounds during ring (**L**) or disk (**M**) phragmoplast stages. Scale bars, 10 µm. (**N**–**P**) CSLM maximum intensity projections of orthogonally resliced images of primary root meristematic epidermal cells in Col-0 (**N**), *kin-12a/b* (**O**) and *kin-12f* (**P**) backgrounds expressing *RFP:TUB6*. Images were taken at 5-min intervals. Scale bars, 5 µm. (**Q**) Violin plots of phragmoplast expansion rates in primary root epidermal and cortex meristematic cells in Col-0, *kin-12a/b* and *kin-12f* backgrounds expressing *RFP: TUB6*. Expansion rate was significantly reduced in the *kin-12a/b* (*P *= 0.00000000645) and *kin-12f *(*P* = 0.000000963) background compared to WT; ANOVA and post-hoc Tukey test. *N* = 64 phragmoplasts for Col-0, 32 for *kin-12a/b* and 37 for *kin-12f*. In box plots, the median is displayed as a line, lower and upper hinges correspond to the 25th and 75th percentiles, the lower and upper whiskers extend from the hinge to the smallest or largest value no further than 1.5 * IQR from the hinge. (**R**) Violin plots of phragmoplast expansion rates in primary root epidermal and cortex meristematic cells in *DEX»RPS5a»RAB-A2a[WT], DEX»RPS5a»RAB-A2a[QL], DEX»RPS5a»RAB-A2a[SN], and DEX»RPS5a»RAB-A2a[NI]*, backgrounds co-expressing *RFP: TUB6*. All treatments are 36 h 5 µM DEX or 36 h equivalent volume DMSO except for RAB-A2a[NI] which is 500 nM DEX. n.s. *P *= 0.998768957; *P = 0.014695577, ***P = 9.992e-15 for RAB-A2aNI, *P* = 7.3525e-11 for RAB-A2aSN; ANOVA and post-hoc Tukey test. *N* = 17 phragmoplasts for WT DMSO, 28 for WT DEX, 23 for DMSO QL, 22 for DEX QL, 25 for DMSO SN, 30 for DEX SN, 25 for DMSO NI, 34 for DEX NI. In box plots, the median is displayed as a line, lower and upper hinges correspond to the 25th and 75th percentiles, the lower and upper whiskers extend from the hinge to the smallest or largest value no further than 1.5 * IQR from the hinge. (**S**, **T**) Airyscan CLSM section of primary root meristematic epidermal cells co-expressing *RFP:TUB6* and *DEX»RAB-A2a[NI]* upon 36 h treatment with 500 nM Dex (NI) or DMSO during disk phragmoplast phase (**S**) and ring phragmoplast phase (**T**). Scale bars, 10 µm. (**U**, **V**) CSLM maximum intensity projections of orthogonally resliced images of primary root meristematic epidermal cells co-expressing *RFP: TUB6* and *DEX»RAB-A2a[NI]* upon 36 h treatment with DMSO (**U**) and 500 nM DEX treatments (**V**). Images were taken at 5-min intervals. Scale bars, 5 µm. Source data are available online for this figure.

Kin-12/B are required for phragmoplast and cell plate formation in *Arabidopsis* microspores (Lee et al, 2007). However, *kin-12a/b* mutants have been reported to have no visible defects in somatic cytokinesis. This indicates that Kin-12A/B may act redundantly with other kinesins (including Kin-12F) during cytokinesis in somatic cells (Lee et al, 2007; Livanos and Müller, 2019). To test this hypothesis, we aimed to abolish Kin-12F function in the *kin-12a/b* background. We first attempted to obtain a triple stable mutant in all three Class II Kin-12s, both via crossing *kin-12a/b* and *kin-12f* SALK mutants as well as constitutive CRISPR-Cas9 against Kin-12F in the *kin-12a/b* background. However, we failed to obtain a triple Class II Kin-12 mutant via these strategies. This is consistent with previous failure to obtain a null mutant in all *Physcomitrium* Class II Kin-12 members^21^. We therefore concluded that triple Class II Kin-12 mutants in *Arabidopsis* are likely lethal, either in the germline or early in somatic development. We therefore used an alternative cell-file-specific CRISPR-Cas9 strategy, and targeted Kin-12F via CRIPSR-Cas9 driven from the atrichoblast-specific *GLABRA2* (Masucci et al, 1996) promoter (*pGL2::kin-12f:cas9-nls-mturquoise*, hereafter *pGL2»kin-12f*, Fig. EV3A). We first confirmed its specificity through co-expression with YFP:Kin12F, which was knocked out in atrichoblast, but not trichoblast cell files in the presence of *pGL2»kin-12f* (Fig. 2A). We next expressed *pGL2»kin-12f* in the *kin-12a/b* background (*kin-12a/b pGL2»kin-12f*), and found that 14 of 19 independent transgenic T2 lines produced small, stunted plants (Figs. 2B and EV3B). Primary roots of these lines showed extensive cytokinesis defects, ranging from misaligned division planes, to incomplete cross-walls, to cells with no sign of cross-wall formation (Figs. 2C–H and EV3C–F). In *kin-12a/b pGL2»kin-12f* primary roots, 29.9% cells were multinucleate with up to 5 nuclei per cell (compared to 0.5% multinucleate cells with up to two nuclei in *kin-12a/b;* Fig. 2H; *P* < 0.001, Chi-squared test). In the remaining 70.1% of *kin-12a/b pGL2»kin-12f* cells that were mononucleate, crosswalls often showed other abnormalities such as misalignment or branching (Fig. EV3D,E).

**Figure EV4:**
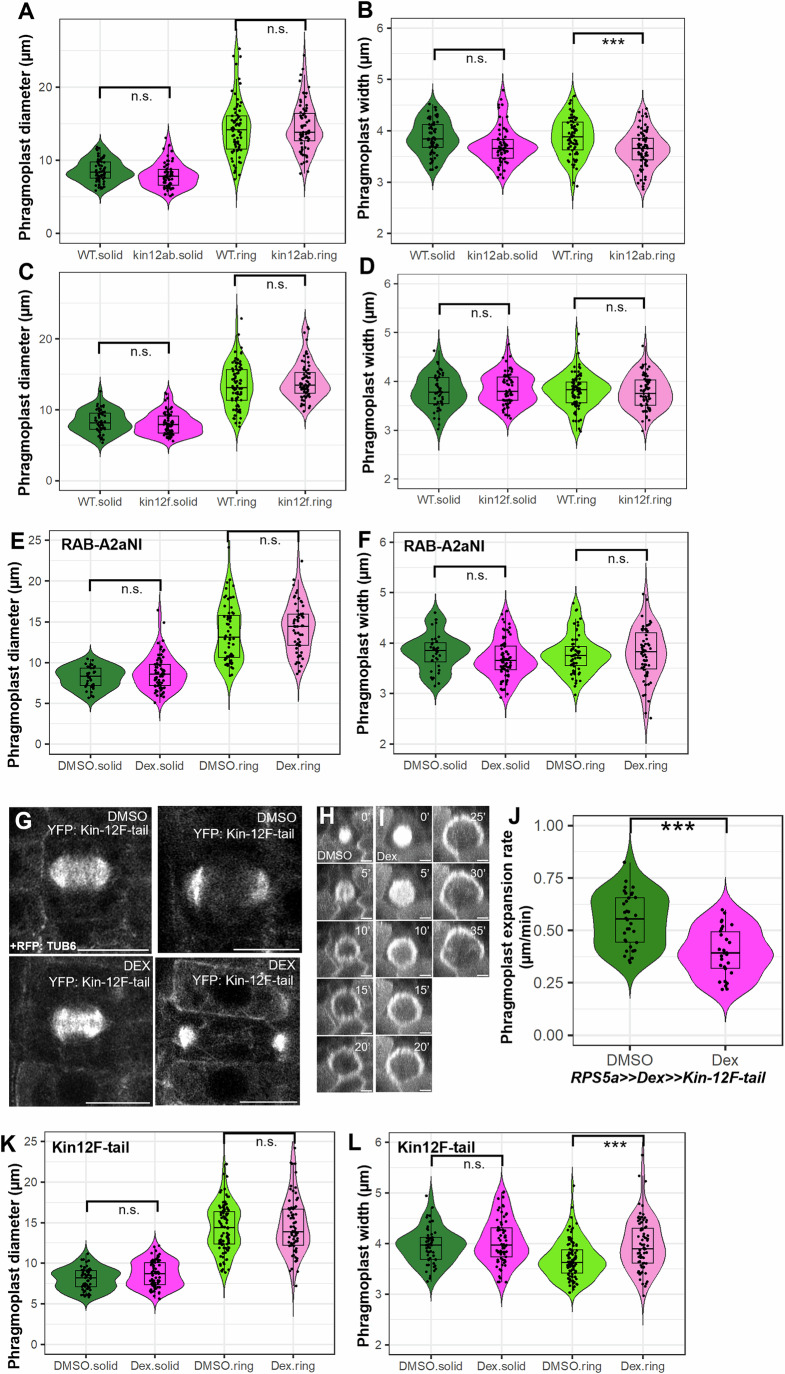
Class II Kin-12 proteins and RAB-A2a are required for phragmoplast expansion. (**A**, **B**) Violin plots of phragmoplast diameter (**A**) and width (**B**) in wild-type and *kin12a/b* backgrounds expressing *RFP:TUB6* during disk and ring phase. *N* = 66 (Col-0 disk), 67 (*kin-12a/b* disk), 73 (Col-0 ring), and 79 (*kin-12a/b* ring). There is a small but statistically significant difference in phragmoplast width between wild-type and *kin12a/b* during ring stage, otherwise phragmoplast morphology is indistinguishable (two-way ANOVA and post-hoc Tukey test, n.s. = *P* ≥ 0.05; ***P < 0.001). (**C**, **D**) Violin plots of phragmoplast diameter (**C**) and width (**D**) in wild-type and *kin-12f* backgrounds expressing RFP:TUB6 during disk and ring phase. *N* = 49 (Col-0 disk), 68 (*kin-12f* ring), 75 (*kin-12f* disk), and 83 (Col-0 ring). Phragmoplast morphology is indistinguishable between wild-type and *kin-12f* (two-way ANOVA and post-hoc Tukey test, n.s. = *P* ≥ 0.05). (**E**, **F**) Violin plots of phragmoplast diameter (**E**) and width (**F**) during disk and ring phase in presence and absence of DEX»RAB-A2aNI expression. *N* = 31 (DMSO disk), 43 (DMSO ring), 50 (Dex ring), and 57 (Dex disk). Phragmoplast morphology is indistinguishable between DMSO and Dex-treated plants (two-way ANOVA and post-hoc Tukey test, n.s. = *P* ≥ 0.05). (**G**) Airyscan CLSM section of primary root meristematic epidermal cells co-expressing RFP:TUB6 and DEX»YFP: Kin-12F-tail upon 72 h treatment with 5 µM Dex or DMSO during disk phragmoplast phase (left) and ring phragmoplast phase (right). (**H**, **I**) Resliced CSLM maximum intensity projections of primary root meristematic epidermal cells co-expressing RFP:TUB6 and DEX»YFP:Kin-12F-tail upon 72 h treatment with DMSO (**H**) or 5 µM Dex (**I**). Images were taken at 5 min intervals. (**J**) Violin plots of phragmoplast expansion rates in primary root meristematic epidermal and cortex cells co-expressing RFP:TUB6 and DEX»YFP:Kin-12F-tail upon 72 h treatment with DMSO or 5 µM Dex. Expansion rate was significantly reduced in in Dex- compared to DMSO-treated plants (*P* = 3.469e-06, Student’s *T* test). *N* = 34 (DMSO), 30 (Dex). (**K**, **L**) Violin plots of phragmoplast diameter (**K**) and width (**L**) during disk and ring phase in the presence and absence of DEX»YFP: Kin-12F-tail expression. *N* = 57 (DMSO disk), 73 (Dex disk), 78 (Dex ring), and 97 (DMSO ring). Phragmoplast morphology is indistinguishable between DMSO and Dex-treated plants apart from phragmoplast width at ring stage, which is increased in the presence of Dex (two-way ANOVA and post-hoc Tukey test, n.s. = *P* ≥ 0.05; ***P < 0.001).

Class II Kin-12s have previously been reported to play a structural role in phragmoplast organization in microspores (Lee et al,^2007^), so we tested whether defects in phragmoplast organization contributed to cytokinesis defects in Class II Kin-12 mutants. We introduced RFP:TUB6 into the *kin-12a/b pGL2»kin-12f* background, and imaged phragmoplasts in primary root epidermal cells of 4-day-old seedlings. We observed phragmoplasts at abnormal angles, consistent with the misalignment of division planes in this background (Fig. 2I), as well as occasional instances where a phragmoplast failed to form entirely after spindle dissolution (Fig. 2J). This is consistent with the presence of multinucleate cells entirely lacking a crosswall (Figs. 2H and EV3F). We quantified phragmoplast expansion rates for phragmoplasts that did form in epidermal root cells, and found that phragmoplast expansion was reduced by 45.4% compared to wild-type controls (Fig. 2K), suggesting Class II Kin-12s were required for both phragmoplast formation and expansion. To test whether similar defects were also present in *kin-12a/b* and *kin-12f* mutants, we introduced RFP:TUB6 into these backgrounds and imaged phragmoplasts in primary root cells of 4-day-old seedlings. In *kin-12a/b* as well as *kin-12f* plants, phragmoplasts were morphologically indistinguishable from those in wild-type plants, apart from a slight reduction in width during ring phase in *kin-12a/b* (Figs. 2L,M and EV4A–D). Unexpectedly, we found that phragmoplast expansion rate was 29.2% slower in *kin-12a/b* compared to wild-type plants, and 23.4% slower in *kin-12f* compared to wild-type plants (Fig. 2N–Q). Despite these reductions in phragmoplast expansion rates, primary roots of *kin-12a/b* and *kin-12f* mutants showed no signs of cytokinesis defects (Figs. 2D and EV3B). We conclude that all Class II Kin-12s collectively contribute to phragmoplast expansion during somatic cytokinesis, which is compatible with previous reports regarding Kin-12A/B function in phragmoplast organization in microspores. However, Kin-12A/B and Kin-12-F are not fully redundant in their function, considering that both *kin-12a/b* and *kin-12f* mutants alone showed significant reductions in phragmoplast expansion.

To understand whether the role of Class II Kin-12s in phragmoplast expansion was linked to their interaction with RAB-A2a, we examined phragmoplast morphology and dynamics when RAB-A2a activity or patterning was perturbed. Over-expression of RAB-A2a[WT] had no significant effect on phragmoplast expansion, while expression of constitutively active RAB-A2a[QL] mildly reduced phragmoplast expansion rate by 21.7% (Fig. 2R). Phragmoplast expansion rate was more substantially reduced in the presence of dominant-negative variants RAB-A2a[SN] (45.9%) and RAB-A2a[NI] (53.6%), comparable to the effect observed in *kin-12a/b pGL2»kin-12f* (Fig. 2Q,R). Both RAB-A2a[SN] and RAB-A2a[NI], the latter to a greater extent, cause cytokinesis defects when over-expressed Chow et al, 2008. However, the morphology of both disk and ring phase phragmoplasts upon RAB-A2a[NI] expression, as quantified by phragmoplast width and diameter, was not affected in our experimental conditions (Figs. 2S–V and EV4E,F). We also tested the effect of YFP:Kin-12F-tail expression on phragmoplast expansion, which alters mCh:RAB-A2a distribution during both interphase and cytokinesis (Figs. 1 and EV2). YFP:Kin-12F-tail expression reduced phragmoplast expansion by 28% compared to controls (Fig. EV4G–J), without substantially altering phragmoplast morphology (Fig. EV4K,L). These data indicate that active RAB-A2a at the midzone is required for normal rates of phragmoplast expansion during cytokinesis, functionally linking vesicle delivery to phragmoplast expansion.

### Class II Kin-12s contribute to membrane targeting to the cell plate

**Figure 3:**
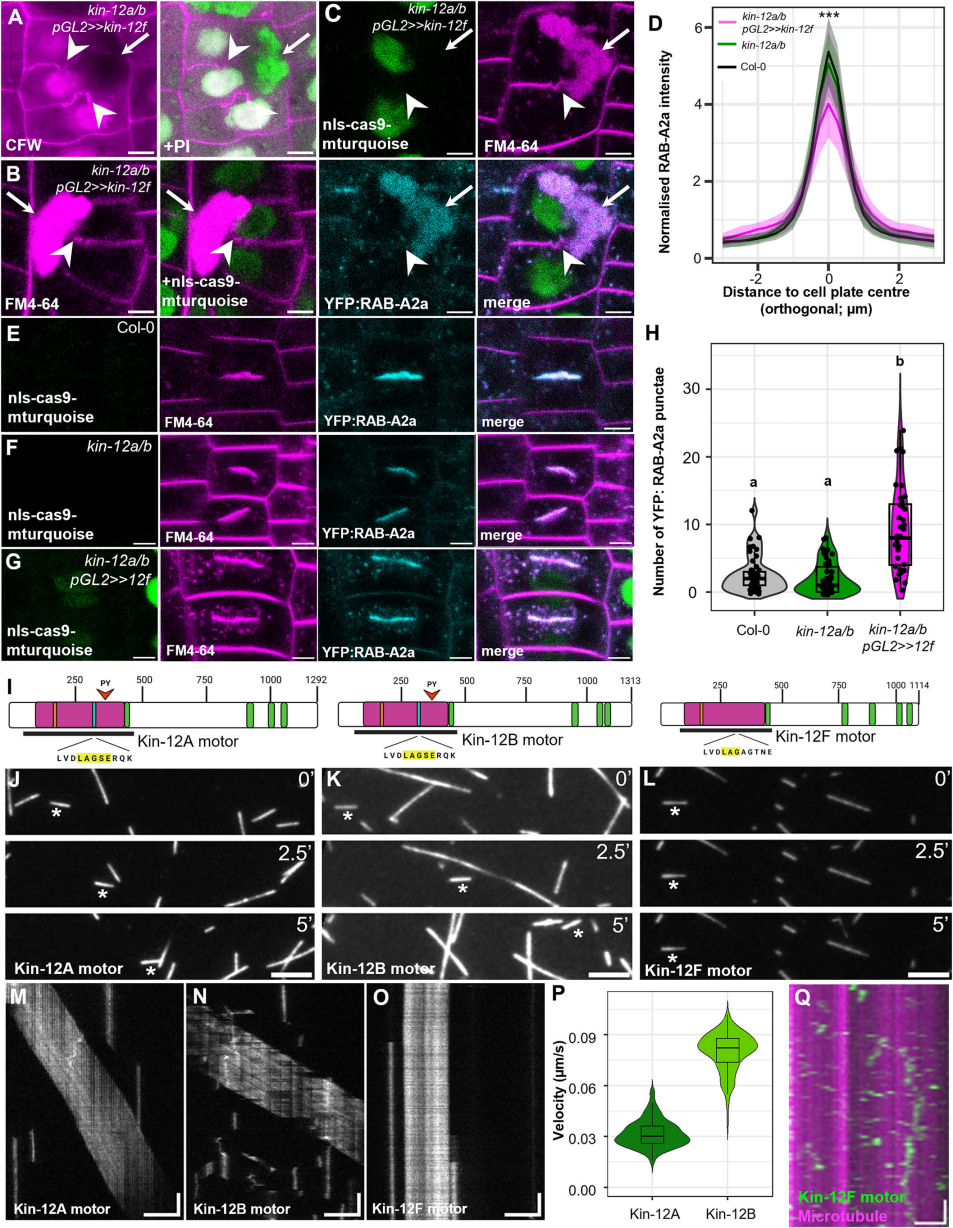
Class II Kin-12 proteins contribute to membrane targeting at the midzone. (**A**) CLSM section of primary root meristematic epidermal cells co-stained with Calcofluor White (CFW) and Propidium Iodide (PI) of *kin-12a/b pGL2»kin-12f* 4-day-old seedlings. Arrowheads indicate incomplete crosswalls, arrow indicates amorphous structure of unconfirmed identity. (**B**) CLSM section of primary root meristematic epidermal cells stained with FM4-64 from *kin-12a/b pGL2»kin-12f* 4-day-old seedlings. Arrowhead indicates incomplete crosswall, arrow indicates amorphous structure of unconfirmed identity. (**C**) CLSM section of primary root meristematic epidermal cells stained with FM4-64 from *kin-12a/b pGL2»kin-12f* 4-day-old seedlings co-expressing *pRAB-A2a::YFP:RAB-A2a*. Arrowhead indicates incomplete crosswall, arrow indicates amorphous structure of unconfirmed identity. (**D**) Mean normalized fluorescence intensity of YFP:RAB-A2a orthogonal to a cell plate in a Col-0 wild-type, *kin-12a/b*, and *kin-12a/b pGL2»kin-12f* background. *N* = 41 (*kin-12a/b pGL2»kin-12f*), 44 (Col-0) and 52 (*kin-12a/b pGL2»kin-12f*). There is a significant difference in YFP:RAB-A2a distribution in *kin-12a/b* compared to Col-0, and in *kin-12a/b pGL2»kin-12f* compared to Col-0 and *kin-12a/b* (two-way ANOVA and post-hoc Tukey test, n.s. = *P* ≥ 0.05; ***P < 0.001). (**E**–**G**) CLSM sections of primary root meristematic epidermal cells expressing YFP:RAB-A2a in Col-0 wild-type (**E**), *kin-12a/b* (**F**) and *kin-12a/b pGL2»kin-12 f* (**G**) backgrounds, counterstained with FM4-64. Scale bars (**A**–**G**), 5 µm. (**H**) Violin plots of number of YFP: RAB-A2a-containing punctae around cell plate in dividing primary root meristematic epidermal cells of Col-0, *kin-12a/*b and *kin-12a/b pGL2»kin-12 f* plants. *N* = 42 cells (Col-0), 49 cells (*12a/b*) and 34 cells (*kin-12a/b, pGL2»kin-12 f*. Same letters denote no significant differences (*P* ≥ 0.05) and different letters denote significant difference (*P* < 0.001), ANOVA and post-hoc Tukey test. In box plots, the median is displayed as a line, lower and upper hinges correspond to the 25th and 75th percentiles, the lower and upper whiskers extend from the hinge to the smallest or largest value no further than 1.5 * IQR from the hinge. (**I**) Schematic depiction of Class II Kin-12 proteins with motor domain truncations highlighted by black lines, note both a complete LASGE motif associated with mobility and a PY motif linked to control of mobility are present in the motor domains of Kin-12A/B but not Kin-12F. (**J**–**L**) TIRF images of Alexa Fluor-568-labelled GMPCPP-stabilized microtubules with Kin-12A motor domain (**J**), Kin-12B motor domain (**K**) and Kin-12F motor domain (**L**). Asterisks indicate same microtubule tracked over time. Scale bars, 10 µm. (**M**–**O**) Kymographs of microtubule gliding assays in the presence of the Kin-12A, -12B, or -12 F motor domain. Note MT gliding activity was observed for Kin-12A and -12B, but not -12F. (**P**) Violin plots of Kin-12A and -12B motor domain velocities calculated from gliding assays shown in (**J-K**). *N* = 180 (Kin-12A) and 224 (Kin-12B). N corresponds to individual microtubules in in vitro gliding assays. In box plots, the median is displayed as a line, lower and upper hinges correspond to the 25th and 75th percentiles, the lower and upper whiskers extend from the hinge to the smallest or largest value no further than 1.5 * IQR from the hinge. (**Q**) TIRF image kymograph of Alexa Fluor-568–labelled GMPCPP-stabilized microtubules (magenta) in the presence of Kin-12F-motor:GFP (green). Note Kin-12F-motor exhibits brief attachment and diffusive motion but no progressive mobility. Source data are available online for this figure.

In *kin-12a/b pGL2»kin-12f* cells, we often observed large amorphous structures positioned close to the end of incomplete cross-walls (present in 74 of 87 multinucleate cells; Fig. 3A,B). We hypothesized these structures may represent mistargeted membrane material of TGN/EE origin. To test this, we expressed *pGL2»kin-12f* in a *kin-12a/b YFP:RAB-A2a* background, and found that amorphous structures accumulated YFP:RAB-A2a (Fig. 3C). In actively dividing *kin-12a/b YFP:RAB-A2a pGL2»kin-12f* plants, we still detected YFP:RAB-A2a at the cell plate (even in cells with amorphous structures), although YFP:RAB-A2a intensity at the midzone was significantly reduced compared to *kin-12a/b* and wild-type plants (Fig. 3D–G; midzone enrichment 4.01 ± 0.87 (*kin-12a/b pGL2»kin-12f)* vs 5.06 ± 0.83 (*kin-12a/b*) vs 5.37 ± 0.87 (wild-type). Simultaneously, we also observed a greater number of YFP:RAB-A2a-positive punctae in the vicinity of the cell plate in *kin-12a/b pGL2»kin-12f* (Fig. 3E–G). We quantified the number of such punctae in dividing cells using a bespoke particle-identification pipeline, and found significantly more punctae in *kin-12a/b pGL2»kin-12f* plants than in *kin-12a/b* and Col-0 plants (Fig. 3H). We concluded that YFP:RAB-A2a-labelled membrane material of TGN/EE origin was partially mistargeted in *kin-12a/b pGL2»kin-12f* mutants, and that membrane mistargeting during cytokinesis likely contributes to the partial or complete failures of cytokinesis in the absence of Class II Kin-12s, in addition to the reduced phragmoplast expansion rate.

**Figure EV5:**
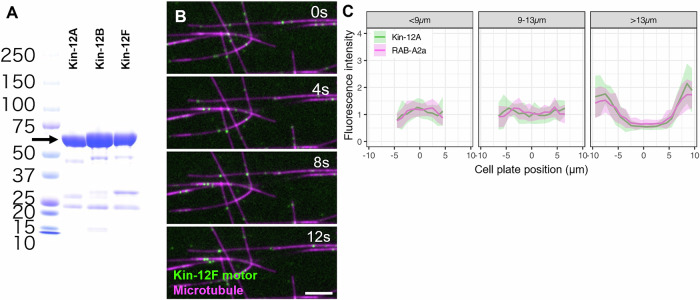
Class II Kin-12 proteins have different in vitro and *in planta* properties. (**A**) Coomassie-stained gel of Kin-12A, Kin-12B, and Kin-12F motor domains used in gliding assays as shown in (Fig 3J–L). Arrow indicates bands corresponding to kinesin motor domains. (**B**) Kymograph of Kin-12F-motor:GFP association with microtubules. Scale bars, 10 µm. (**C**) Fluorescence intensity of mCl: Kin-12A and mCh: RAB-A2a along cell plates. Cell plates were grouped by diameter into short ( < 9 µm), medium (9–13 µm) and long ( > 13 µm). Lines are mean values, shaded areas are +/- 1 SD. N = 11 (mCl:Kin-12A short, mCl: Kin-12A medium, mCl: Kin-12A long), 14 (YFP:Kin-12F medium).

Mistargeting of TGN/EE-derived membranes during cytokinesis in *kin-12a/b pGL2»kin-12f* could be explained by a previously unrecognized role of *Arabidopsis* Class II Kin-12s in vesicle transport towards the midzone. Consistent with this, homologous Class II Kin-12s proteins in *Physcomitrium patens* were recently reported to transport TGN-derived vesicles towards the midzone (Yamada et al, 2025). To examine motility of *Arabidopsis* Class II Kin-12s, we expressed and purified the motor domains of Kin-12A, -B and -F from *E.coli* cultures (Fig. EV5A), and performed in vitro gliding assays to test motor activity (Jonsson et al, 2015) (Fig. 3I–P). In these experiments, both Kin-12A/B, but not Kin-12F demonstrated motor activity (Fig. 3I–P). We quantified motor velocities from gliding assays and found Kin-12B velocities to be significantly higher than Kin-12A velocities (0.080 ± 0.011 µm/s vs 0.031 ± 0.008 µm/s, respectively; Fig. 3P). We further investigated the microtubule-binding ability of Kin-12F by performing a single-molecule motility assay. In this assay, the Kin-12F motor domain was observed to bind to microtubules, but did not exhibit detectable processive movement along the microtubules (Figs. 3Q and EV5B). These differences in motility behaviour between Kin-12A/B and Kin-12F correlate with the presence of the LAGSE motif in Kin-12A/B, which is required for ATP hydrolysis and motility of other kinesins (Parke et al, 2010; Cross and McAinsh, 2014) (Fig. 3I) and is incomplete in Kin-12F (Fig. 3I). Kin-12F also lacks the conserved PY motif found in Kin-12A/B and other kinesins, which has been linked to control of motility (Ganguly et al, 2018; Müller and Livanos, 2019) (Fig. 3I). Taken together, these findings indicate that Kin-12A/B and Kin-12F collectively contribute to membrane targeting during cell division but differ in their precise function, as only Kin-12A/B, but not Kin-12F exhibit the motility required for vesicle transport towards the cell plate.

### Class II Kin-12s exhibit functional differences

**Figure 4:**
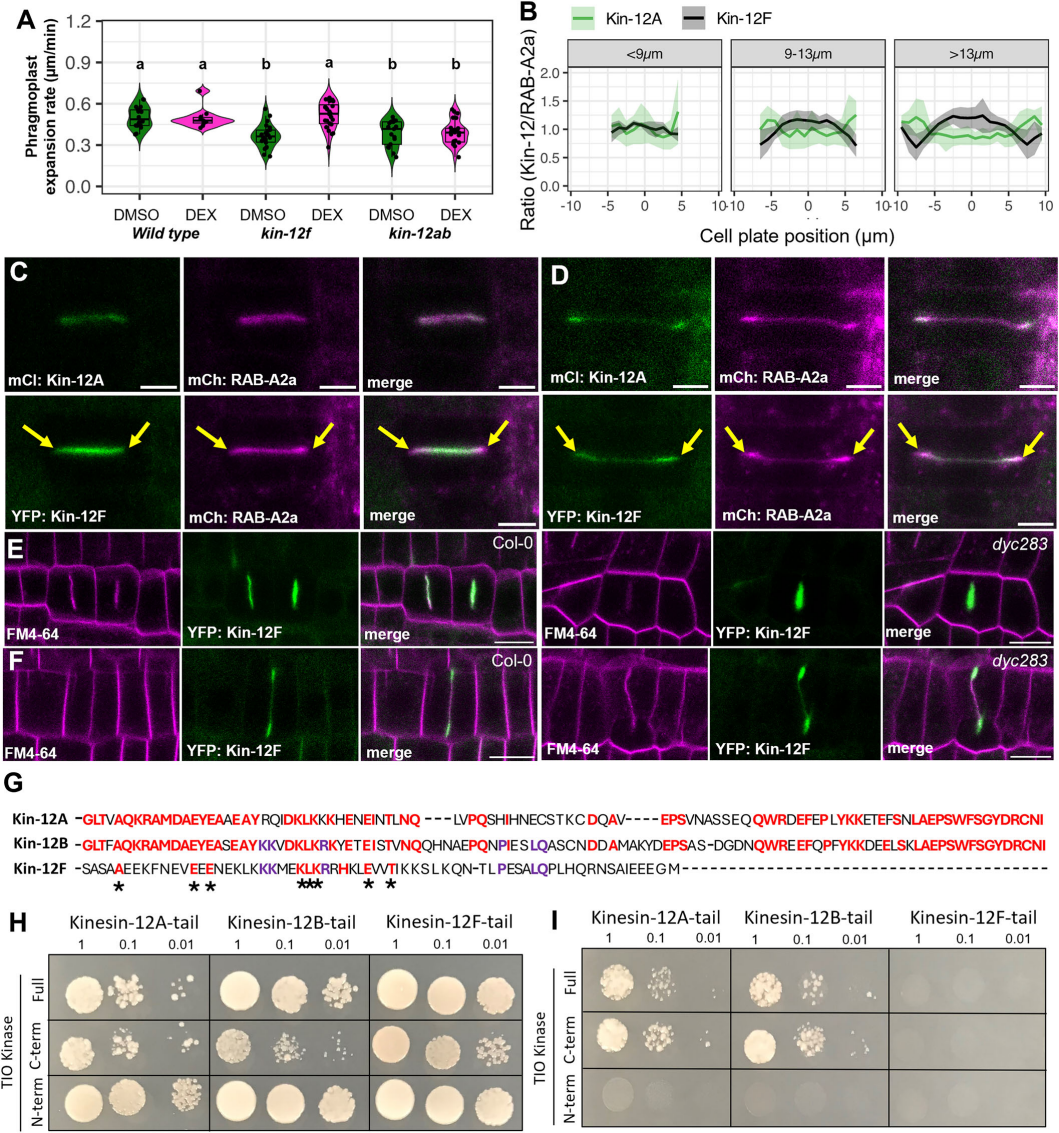
Class II Kinesin-12 members have differing localizations, upstream regulators and interactors. (**A**) Violin plots of phragmoplast expansion rates in primary root epidermal and cortex meristematic cells in Col-0 (*RFP: TUB6*) background or *kin-12f* and *kin-12a/b* backgrounds co-expressing YFP: Kin-12F. 24 h 5 µM DEX or equivalent volume DMSO. Same letters denote no significant differences (*P *≥ 0.05) and different letters denote significant difference (*P* < 0.05), ANOVA and post-hoc Tukey test. *N* = 18 phragmoplasts for Col-0 DMSO, 10 for Col-0 DEX, 22 for *kin-12f* DMSO, 24 for *kin-12f* DEX, 17 for *kin-12a/b* DMSO, 24 for *kin-12a/b* DEX. (**B**) Fluorescence intensity ratio of mCl: Kin-12A (green)/YFP: Kin-12F (grey) relative to mCh: RAB-A2a along cell plates such as that shown in (**B**, **C**). YFP:Kin-12F data quantified is the same as that presented in Fig. 1. Cell plates were grouped by diameter into short ( < 9 µm), medium (9-13 µm) and long ( > 13 µm). Lines are mean values, shaded areas are +/− 1 SD. *N* = 7 (YFP:Kin-12F long), 10 (YFP:Kin-12F short), 11 (mCl:Kin-12A short, mCl: Kin-12A medium, mCl: Kin-12A long), 14 (YFP:Kin-12F medium). (**C**, **D**) CLSM section of primary root meristematic epidermal cell co-expressing *DEX»mCl: Kin-12A/DEX»YFP: Kin-12F* and *mCh: RAB-A2a* during early/disk (**B**) and late/ring (**C**) stages of cytokinesis. Yellow arrows indicate enrichment of mCh: RAB-A2a at leading edge ahead of YFP: Kin-12F. Scale bars 5 µm. (**E**, **F**) CLSM section of primary root meristematic epidermal or cortical cells in Col-0 wild-type and *dyc283* (*map65-3*) expressing *YFP:Kin-12F* at early/disk (**E**) and late/ring (**F**) stage cytokinesis, counterstained with FM4-64. Scale bars, 10 µm. (**G**) Amino acid sequence alignments of conserved residues in known TIO interaction domain of Kin-12A/B compared to Kin-12F. Red indicates residues conserved between Kin-12A/B or Kin-12A/F, purple indicates residues conserved between Kin-12B/F, asterisks indicate residues conserved between all three kinesins. (**H**, **I**) Pairwise Y2H tests between Kin-12 tail regions and TIO kinase variants on SD-Leu-Trp (**H**) and SD-Leu-Trp-Ade-His (**I**). Source data are available online for this figure.

To further explore functional differences between Kin-12A/B and Kin-12F, we first used a complementation approach, and co-expressed *Dex»YFP:Kin-12F* and *RFP:TUB6* in *kin-12f* and *kin-12a/b* backgrounds. We then quantified phragmoplast expansion rates after induction of *YFP:Kin-12F* (Fig. 4A). In the presence of YFP:Kin-12F, phragmoplast expansion rates returned to wild-type levels in the *kin-12f*, demonstrating that the YFP:Kin-12F fusion protein was functional and sufficient to complement the loss of wild-type *kin-12f*. However, expression of YFP:Kin-12F did not increase phragmoplast expansion rates in the *kin-12a/b* mutant, supporting the notion that Kin-12F and Kin-12A/B are not functionally redundant.

Next, we conditionally expressed mClover:Kin-12A (*AtRPS5a»Dex»mCl:Kin-12A*), and examined its localization at the expanding cell plate. Like YFP:Kin-12F, mCl:Kin-12A localized to the phragmoplast midzone and was enriched at the cell plate edges during ring stage (Figs. 4B–D and EV5C). However, when co-expressed with mCh:RAB-A2a, YFP:Kin-12A was enriched at the very outermost region of the cell with mCh:RAB-A2a, while YFP:Kin-12F was depleted at this outermost edge compared to mCh:RAB-A2a (Figs. 1H,J,  4B–D, and EV5C). These results indicate that Kin-12A and Kin-12F occupy subtly distinct domains at the cell plate, with Kin-12A preferentially enriched at the outermost leading edge (where the phragmoplast expands and new membrane material is added) and Kin-12F preferentially enriched at the lagging edge (where the vesicles fuse into a continuous cell plate and the phragmoplast is disassembled). This *in planta* difference adds further support to our in vitro work indicating that Kin-12A/B are motile, as motile kinesins involved in vesicle delivery would be expected to be enriched at the outermost leading edge where vesicle deliver occurs.

We also observed differences within the Class II Kin-12 subclass with regard to their interactors. In *Arabidopsis*, stable targeting of Kin-12A to the midzone depends on the midzone-localized microtubule crosslinker MAP65-3Ho et al, 2011, which is required for microtubule interdigitation at the leading, but not the lagging edge of the expanding phragmoplast. When we expressed YFP:Kin-12F in the *map65-3* mutant *dyc283* (Caillaud et al, 2008), YFP:Kin-12F continued to localize to the phragmoplast midzone (Fig. 4E,F), demonstrating that unlike Kin-12A/B, Kin-12F could localize to the midzone independently of MAP65-3. Furthermore, Kin-12A/B have previously been shown to interact with TWO-IN-ONE (TIO), the sole *Arabidopsis* ortholog of a widely-conserved kinase known as Fused in *Drosophila* and Stk36 in humans, which in non-plant lineages is involved in control of cell proliferation, differentiation, and some microtubule organization processes (Nozawa et al, 2013; Lee et al, 2023; McCoy et al, 2023). The binding domain sequence of TIO kinase on Kin-12A/B (Oh et al, 2012) is poorly conserved in the Kin-12F sequence in comparative alignments (Fig. 4G). Consistent with this, we could find no evidence for an interaction between the Kin-12F tail region and TIO in Y2H (Fig. 4H,I). Taken together, our data demonstrates that although Class II Kin-12s are collectively required for proper somatic cytokinesis, Kin-12A/B and Kin-12F differ in their activity, subcellular localization and interaction partners.

### TIO kinase is positioned during cytokinesis by Kin-12A/B and RAB-A2a

**Figure 5:**
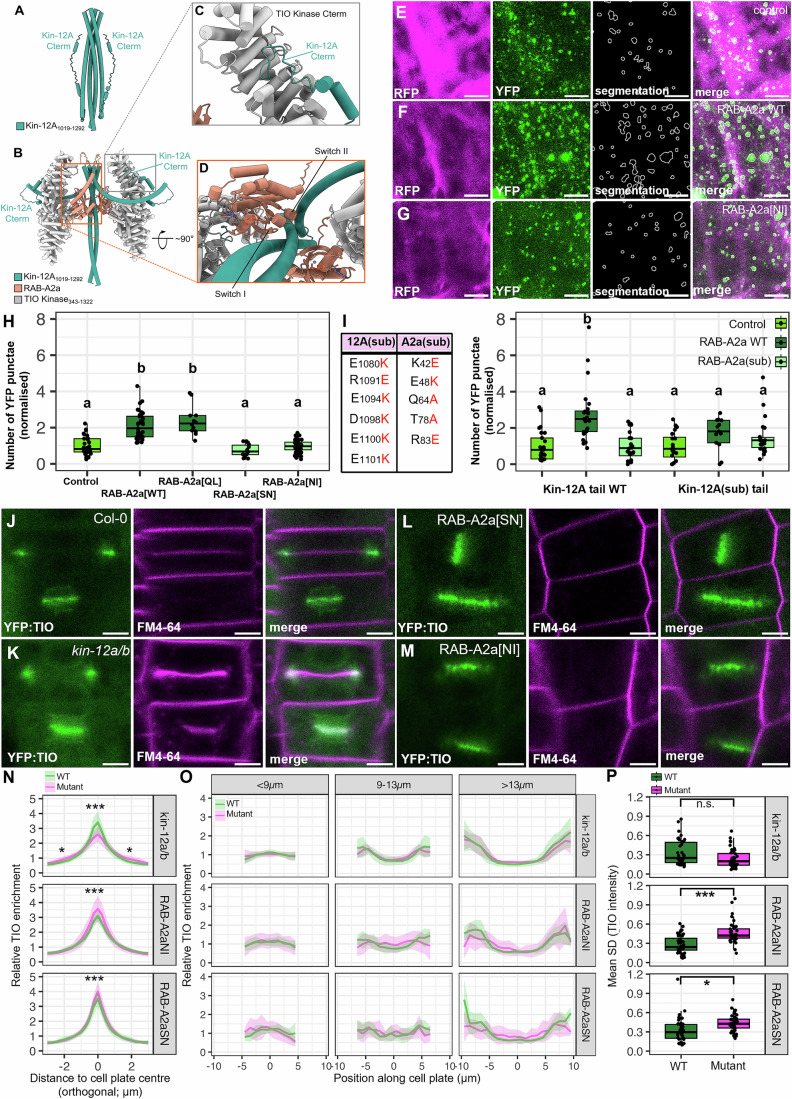
Kin-12A&B and RAB-A2a position TIO kinase during cytokinesis. (**A**) AF3 rendering showing predicted dimerization of Kin-12A tail regions, consistent with previous reports Pan et al, 2004. (**B**–**D**) AF3 renderings showing predicted interaction structure and positions of Kin-12A tail dimer, RAB-A2a and TIO kinase C-terminus. Note that the interaction interface between RAB-A2a and Kin-12A features the GTPase switch regions that undergo conformational changes upon nucleotide binding (**D**). AF3 also predicts the existence of adjacent interfaces between RAB-A2a and TIO kinase within the module (B&D). (**E**–**G**) Cropped sections of segmented *Nicotiana benthamiana* epidermal leaf cells transiently expressing *nYFP:TIO C-terminus* with *cYFP: Kin-12A tail* and *mRFP1* from a ratiometric rBiFC vector alone at O.D. 600 0.1 (**E**) or alongside wild-type RAB-A2aWT (**F**), or RAB-A2aNI (**G**) at O.D. 600 0.1 (combined O.D. 600 of 0.2). Scale bars 5 µm. (**H**) Quantification of number of YFP punctae per area normalized against mean RFP fluorescence from images as those shown in (**E**–**G**). Same letters denote no significant differences (*P* ≥ 0.05) and different letters denote significant difference (*P *< 0.05), ANOVA and post-hoc Tukey test. The large number of exact *P* values associated with this plot are included in the source data document. Note the presence of RAB-A2a[WT] and RAB-A2a[QL], but not RAB-A2a[NI] or RAB-A2a[SN] significantly increased number of YFP punctae. Cell area, mean RFP intensity, or mean intensity of punctae did not significantly differ between conditions (one-way ANOVA and post-hoc Tukey test, *P* ≥ 0.05). *N* = 31 cells (control), 39 cells (A2aWT), 12 cells (A2aQL), 40 cells (A2aSN), 14 cells (A2aNI) over 3 independent experiments. In box plots, the median is displayed as a line, lower and upper hinges correspond to the 25th and 75th percentiles, the lower and upper whiskers extend from the hinge to the smallest or largest value no further than 1.5 * IQR from the hinge. (**I**) Quantification of number of YFP punctae per area normalized against mean RFP fluorescence in cells transiently expressing *nYFP:TIO C-terminus* with *cYFP: Kin-12A tail/cYFP: Kin-12A(sub) tail* and *mRFP1* from a ratiometric rBiFC vector alone at O.D. 600 0.1, or alongside RAB-A2aWT/RAB-A2a(sub) at O.D. 600 0.1 (combined O.D. 600 of 0.2). *N* = 25 cells (12 A WT alone), 24 cells (12 A WT + A2a WT), 24 cells (12 A WT + A2A sub), 20 cells (12 A sub alone), 14 cells (12 A sub + A2a WT), 22 cells (12 A sub + A2a sub) over 3 independent experiments. Same letters denote no significant differences (*P *≥ 0.05) and different letters denote significant difference (*P* < 0.05), ANOVA and post-hoc Tukey test. The large number of exact *P* values associated with this plot are included in the source data document. In box plots, the median is displayed as a line, lower and upper hinges correspond to the 25th and 75th percentiles, the lower and upper whiskers extend from the hinge to the smallest or largest value no further than 1.5 * IQR from the hinge. (**J**–**M**) CLSM sections of primary root meristematic epidermal cells expressing Dex»YFP: TIO in wild-type (**J**), *kin-12a/b* (**K**), *DEX»RAB-A2a[SN]* (**L**) *or Dex»RAB-A2a[NI]* (**M**) backgrounds. Roots were co-stained with FM4-64. 36 h, 5 µM DEX. (**N**) YFP: TIO enrichment orthogonal to the cell plate direction in wild-type (green) or *kin-12a/b* background or backgrounds expressing RAB-A2a[NI] and RAB-A2a[SN] (pink) from images such as (**J**–**M**). Two-way ANOVA and post-hoc Tukey test; *P < 0.05 (*P* = 0.027831929), ***P < 0.001 (*P* < 10e-24 for *kin-12a/b* and RAB-A2aSN; *P *= 1.0588e-09 for RAB-A2aNI). *N* = 67 (*kin-12a/b* WT control), 70 (*kin-12a/b*), 43 (RAB-A2aNI WT control, RAB-A2a, RAB-A2aSN control), 38 (RAB-A2aSN). (**O**) YFP: TIO enrichment parallel to the cell plate direction in wild-type (green) or *kin-12a/b* background or backgrounds expressing RAB-A2a[NI] and RAB-A2a[SN] (pink) from images such as (**J**–**M**). Cell plates were grouped by diameter into short ( < 9 µm), medium (9–13 µm) and long ( > 13 µm). Lines are mean values, shaded areas are +/− 1 SD. *N* = 12 (*kin-12a/b* medium), 19 (Col-0 medium), 23 (Col-0 long), 26 (*kin-12a/b* long), 28 (Col-0 short), and 35 (*kin-12a/b* short). (**P**) Mean standard deviation of YFP: TIO signal intensity parallel to cell plate direction in wild-type (green) or *kin-12a/b* background or backgrounds expressing RAB-A2a[NI] and RAB-A2a[SN] (pink) from images such as (**J**–**M**). Two-way ANOVA and post-hoc Tukey test: n.s. = non-significant (*P* = 0.0677079); *P < 0.05 (*P* = 0.0132094); ***P < 0.001 (*P* = 0.0000031). *N* = 44 for all samples, which corresponds to the SD at each position of the cell plate in 1 µm intervals for the samples shown in 5 P. Short, medium, and long cell plates were all included. In box plots, the median is displayed as a line, lower and upper hinges correspond to the 25th and 75th percentiles, the lower and upper whiskers extend from the hinge to the smallest or largest value no further than 1.5 * IQR from the hinge. Source data are available online for this figure.

**Figure EV6:**
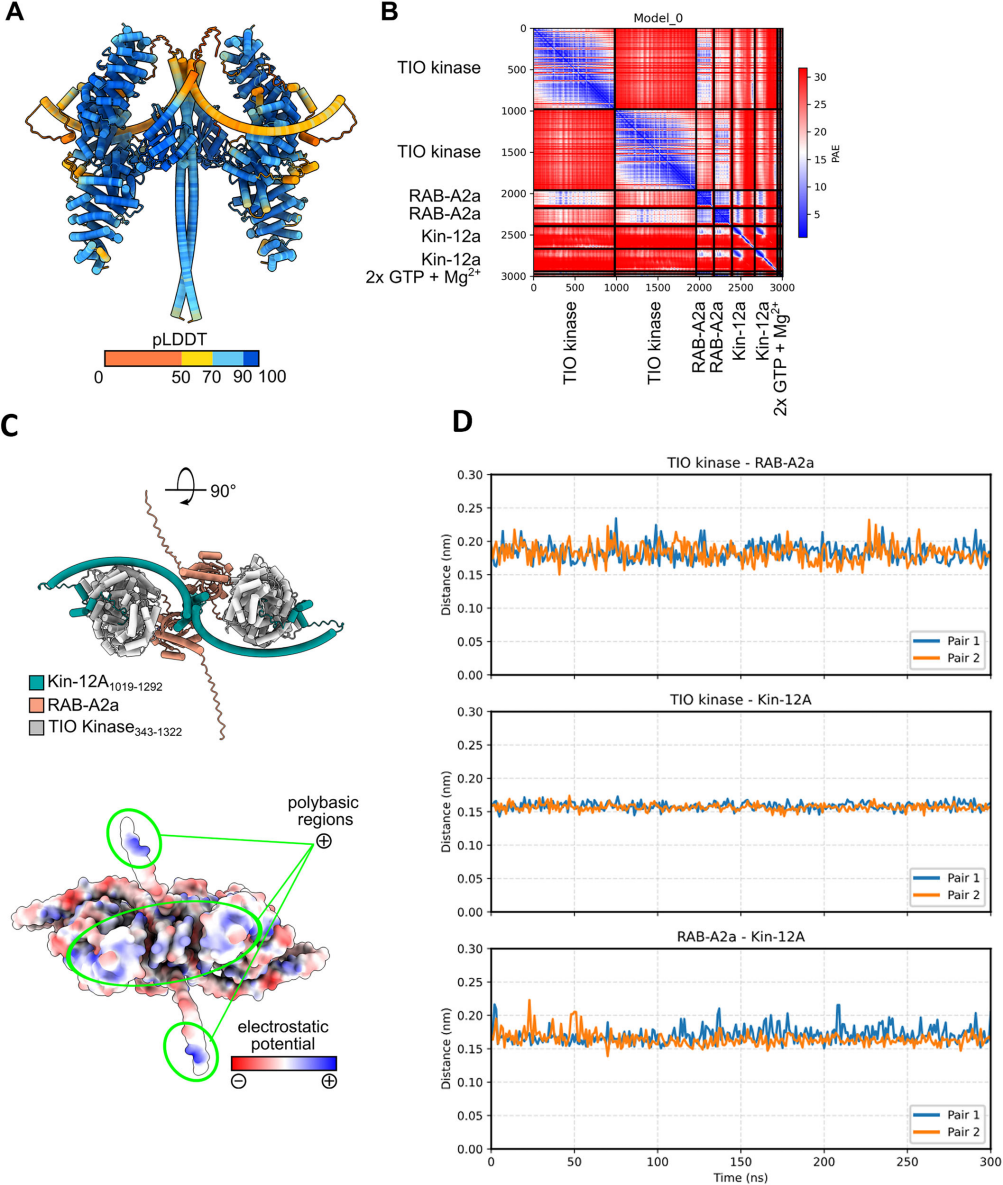
AF3 prediction parameters and molecular dynamics prediction for the RAB-A2a/Kin-12A/TIO module. (**A**) Confidence of AF3 local structure predictions as measured by predicted Local Distance Difference Test (pLDDT). Blue indicates highest possible confidence. (**B**) Predicted Alignment Error (PAE, Angstroms) measure of the confidence of relative positions of different residues within the AF3 predicted structure. (**C**) AF3 renderings showing base of RAB-A2a/Kin-12A/TIO kinase module with amino acid electrostatic potentials indicated. Coulombic electrostatic potential representation of the RAB-A2a/Kin-12A/TIO module. The base of the module features a high proportion of polybasic residues (positively charged), which align positionally with the negatively-charged anionic lipid composition of the cell plate. (**D**) Minimal distance (nm) between the interacting subunits of the RAB-A2a/Kin-12A/TIO kinase complex in all-atom molecular dynamics simulation over 300 nanoseconds. Pair 1 and Pair 2 refer to the two individual pairs in the hexamer complex (e.g. two pairs each for RAB-A2a/Kin-12A etc.). As for the AF3 work in Fig. 5A, the simulation incorporates the C-terminal regions of Kin-12A and TIO kinase, and the full sequence of RAB-A2a, in an overall hexamer complex. Movie version of simulation attached as Movie EV1.

**Figure EV7:**
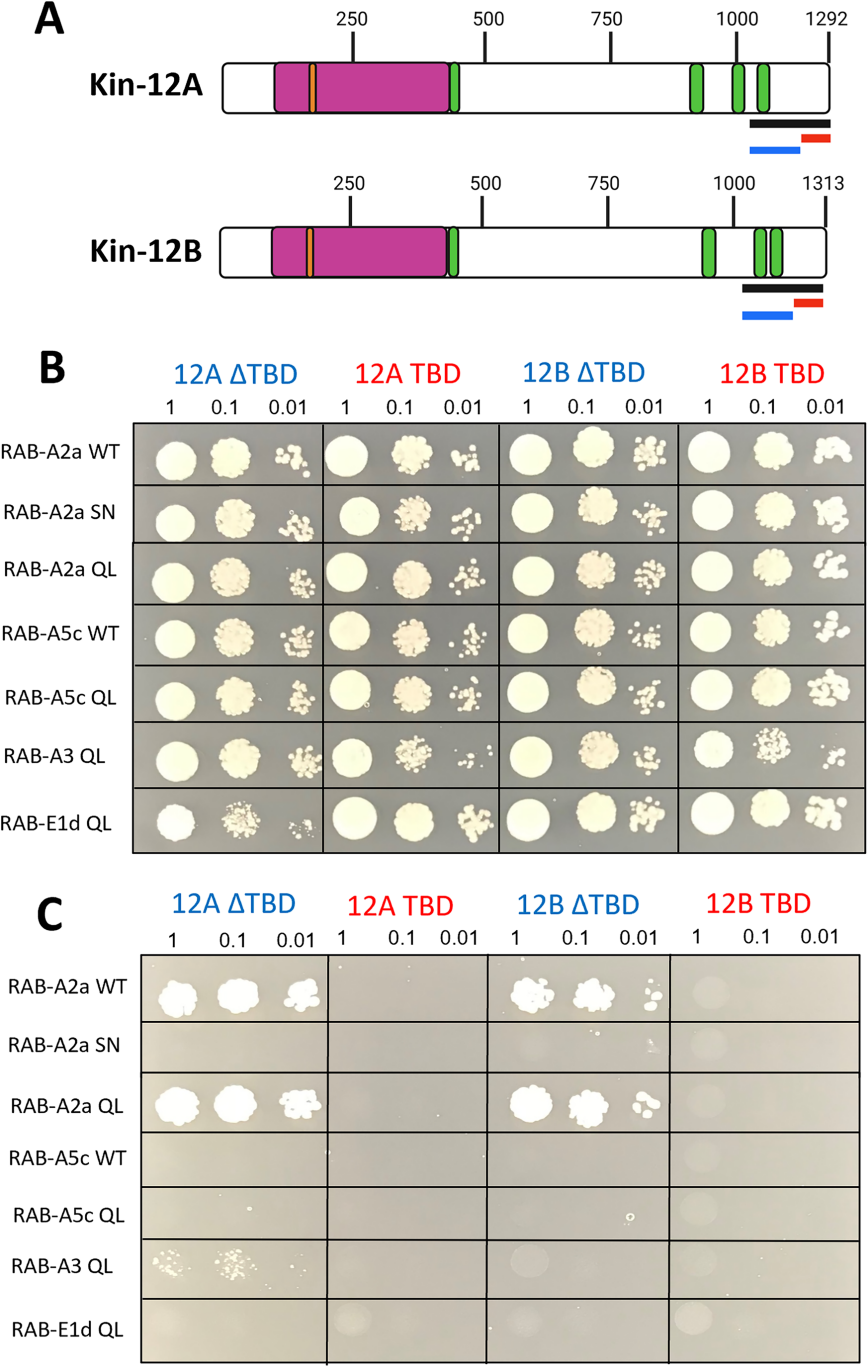
The binding domains of RAB-A2a and TIO on Kin-12A/-12B are adjacent but do not overlap. (**A**) Schematic depiction of Class II Kin-12s structure and binding domains of RAB-A2a and TIO. Black lines: clone region isolated as interactors of RAB-A2a in initial Y2H screen. Red lines: known TIO binding domains on Kin-12A/B. Blue lines: refined interaction domains of RAB-A2a (12 A/B ΔTBD) based on Y2H. (**B**, **C**) Pairwise Y2H tests between Kin-12A&B tail region truncations and Rab-A GTPase variants on SD-Leu-Trp (**B**) and SD-Leu-Trp-Ade-His (**C**).

TIO is required for cytokinesis in both reproductive and somatic cells of *Arabidopsis* (Oh et al, 2005), and has been identified as a positive regulator of phragmoplast expansion in *Arabidopsis* microspores (Oh et al, 2012). This raises the question of whether the roles of Class II Kin-12s and RAB-A2a in phragmoplast expansion are linked to TIO kinase function? We first explored this in silico. Alphafold3 (AF3) predicts with high confidence an interaction interface between the nucleotide-binding switch-I and switch-II regions of RAB-A2a and the tails of Kin-12A (approximately amino acids 1070–1130), Kin-12B (approximately amino acids 1095–1140) and Kin-12F (approximately amino acids 930–980), consistent with our Y2H results (Figs. 5A–D and 1A,B). AF3 also predicted a triple interaction between Kin-12A, RAB-A2a and TIO kinase in an overall hexamer complex, with high confidence of the relative positions of RAB-A2a and TIO kinase on the Kin-12A tail region; indicating that their respective binding domains are adjacent but separate on the kinesin tail (Figs. 5A–D and EV6A–C). AF3 predictions also indicate that within the RAB-A2a/Kin-12A/TIO module, binding interfaces also exist between RAB-A2a and TIO kinase (Fig. 5B–D), which may further stabilize the complex. Next, we performed an all-atom molecular dynamics simulation to assess whether the Kin-12A/RAB-A2a/TIO kinase hexamer complex remains stable over time. As demonstrated by the constant minimal distance between interacting subunits within the complex, the three combinations of binding interfaces between Kin-12A, RAB-A2a and TIO kinase in complex remained stable across the simulation time (Fig. EV6D; Movie EV1). To complement our in silico predictions with experimental data, we fine-mapped the interaction domains between Class II Kin-12s, RAB-A2a and TIO kinase in Y2H. We expressed truncated variants of the Kin-12A/B tail regions that encompassed either the previously described TIO-Binding Domain (TBD) (Oh et al, 2005), or the remainder of the tail region we identified as the RAB-A2a interaction domain (Fig. 1A,B) without the TIO binding domain (ΔTBD; Fig. EV7A). In Y2H, neither Kin-12A-TBD nor Kin-12B-TBD interacted with RAB-A2a, its mutant variants, or other Rab GTPases known to localize to the cell plate (Fig. EV7B,C). However, both Kin-12A-tailΔTBD and Kin-12B-tailΔTBD interacted with RAB-A2a WT and RAB-A2a[QL] in Y2H (Fig. EV7B,C). These results confirm the in silico prediction that the TIO and RAB-A2a binding domains of Kin-12A/B are adjacent and non-overlapping. AF3 predicts that upon binding to RAB-A2a and TIO kinase, the tail region of Kin-12A is stabilized into a more open conformation, raising the possibility that RAB-A2a binding to Kin-12A may promote TIO recruitment through increasing steric accessibility (Figs. 5B–D and EV6A–C).

**Figure EV8:**
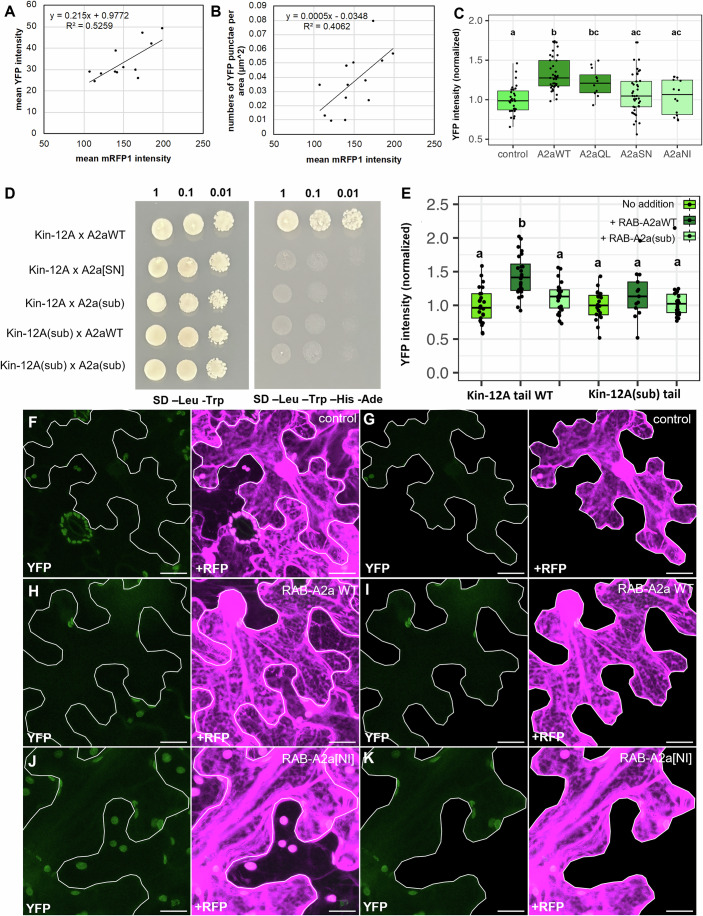
Additional data relating to rBiFC assays. (**A**, **B**) Correlation of mean YFP intensity (**A**) or mean YFP punctae per area (**B**) with mean mRFP1 intensity from *N. benthamiana* epidermal leaf cells transiently expressing nYFP:TIO and cYFP:Kin-12A and mRFP1 from a ratiometric BiFC system. (**C**) Quantification of YFP intensity normalized against mean RFP fluorescence from epidermal leaf cells transiently expressing nYFP:TIO and cYFP:Kin-12A and mRFP1 from a ratiometric BiFC system (control) or alongside RAB-A2a variants. Same letters denote no significant differences (*P* ≥ 0.05) and different letters denote significant difference (*P *< 0.05), ANOVA and post-hoc Tukey test. Note the presence of RAB-A2a[WT] and RAB-A2a[QL], but not RAB-A2a[NI] or RAB-A2a[SN] significantly increased number of YFP punctae. *N* = 31 cells (control), 39 cells (A2aWT), 12 cells (A2aQL), 40 cells (A2aSN), 14 cells (A2aNI). (**D**) Pairwise Y2H tests between WT and substituted variants of Kin-12A tail region and RAB-A2a on SD-Leu-Trp and SD-Leu-Trp-Ade-His. (**E**) Quantification of mean YFP intensity per area normalized against mean RFP fluorescence in cells transiently expressing *nYFP:TIO C-terminus* with *cYFP: Kin-12A tail/cYFP: Kin-12A(sub) tail* and *mRFP1* from a ratiometric rBiFC vector alone at O.D. 600 0.1, or alongside RAB-A2aWT/RAB-A2a(sub) at O.D. 600 0.1 (combined O.D. 600 of 0.2). *N* = 25 cells (12A WT alone), 24 cells (12A WT + A2aWT), 24 cells (12A WT + A2a sub), 20 cells (12A sub alone), 14 cells (12A sub + A2a WT), 22 cells (12A sub + A2a sub) over 3 independent experiments. Same letters denote no significant differences (*P* ≥ 0.05) and different letters denote significant difference (*P* < 0.05), ANOVA and post-hoc Tukey test. (**F**–**K**) CLSM maximum intensity projections of *N. benthamiana* epidermal leaf cells transiently expressing nYFP:TIO and cYFP:Kin-12F and mRFP1 from a ratiometric BiFC system on its own (**F**, **G**) or alongside RAB-A2aWT (**H**, **I**) or RAB-A2aNI (**J**, **K**) before (**F**, **H**, **J**) or after (**G**, **I**, **K**) cell segmentation. Note no YFP signal was detected, but chloroplast autofluorescence is visible. Scale bars, 20 µm.

To test whether RAB-A2a binding to Kin-12A can promote the interaction between TIO and Kin-12A, we first used a ratiometric bimolecular fluorescence complementation (rBiFC) vector system (Grefen and Blatt, 2012). This system allows ratiometric expression of two proteins of interest fused to the N- or C-terminus of a split eYFP, alongside mRFP1 as an expression control all from the same vector (Grefen and Blatt, 2012) (Fig. EV8A,B). We used this system to transiently express nYFP:TIO-C-term with either cYFP:Kin-12A-tail or cYFP:Kin-12F-tail (as a negative control) in *Nicotiana benthamiana* leaves. We consistently observed YFP punctae in cells co-expressing nYFP:TIO-C-term and cYFP:Kin-12A-tail (Fig. 5E–G), but never in cells co-expressing nYFP:TIO C-term and cYFP:Kin-12F-tail (Fig. EV8F–K). To test whether expression of *RAB-A2a* had an effect on the TIO/Kin-12A interaction, we co-expressed wild-type RAB-A2a and RAB-A2a mutant variants with the rBiFC construct. We found that co-expression of wild-type RAB-A2a and constitutively active RAB-A2a[QL] significantly increased both numbers of YFP punctae per cell and mean YFP fluorescence (Figs. 5E–H and EV8C). This was not the case when we co-expressed dominant-negative RAB-A2a[SN] or RAB-A2a[NI] (Figs. 5G,H and EV8C). In addition to these commonly used point-mutations that impact the nucleotide-bound status of RAB-A2a, we also targeted the binding interface between Kin-12A and RAB-A2a. We computationally identified amino acid residues within the binding interface between the Kin-12A tail and RAB-A2a that were predicted to be required for their interaction. These identified amino acids mostly feature charged polar sidechains (Fig. 5I). We then attempted to destabilize the Kin-12A/RAB-A2a interaction through site-directed mutagenesis against these amino acids, producing Kin-12A(sub) and RAB-A2a(sub) respectively (Fig. 5I). In Y2H, Kin-12A(sub) and RAB-A2a(sub) did not interact with wild-type RAB-A2a and Kin-12A respectively, nor with each other (Fig. EV8D). We then introduced Kin-12A(sub) and RAB-A2a(sub) into our rBiFC system. Unlike wild-type RAB-A2a, the presence of RAB-A2a(sub) failed to significantly increase YFP punctae per cell and mean YFP fluorescence when co-expressed with cYFP:Kin-12A-tail and nYFP:TIO-C-term (Figs. 5I and EV8E). Moreover, when co-expressed with cYFP:Kin-12A(sub)-tail and nYFP:TIO-C-term, neither wild-type RAB-A2a nor RAB-A2a(sub) produced a significant increase in mean YFP punctae per cell and fluorescence (Figs. 5I and EV8E). Collectively, these data indicate that (1) the presence of RAB-A2a could enhance the interaction between TIO and Kin-12A *in planta* and (2) this enhancement was dependent on RAB-A2a activity, and the compatibility of the binding interface between RAB-A2a and Kin-12A.

**Figure EV9:**
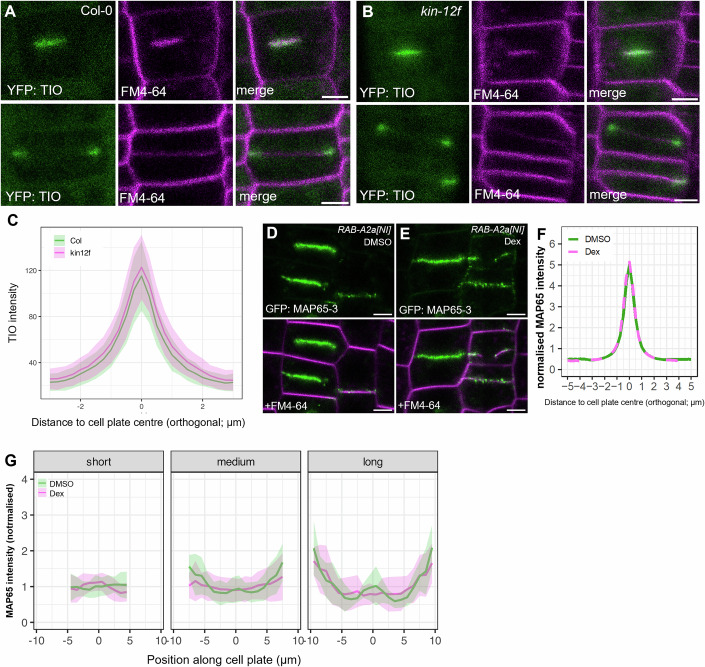
Kin-12F does not affect TIO kinase intensity at the midzone and MAP65-3 localization at the midzone is not affected by expression of RAB-A2a[NI]. (**A**, **B**) CLSM sections of primary root meristematic epidermal cells expressing Dex»YFP: TIO in wild-type (**A**) and *kin-12f* (**B**). Roots were co-stained with FM4-64. Scale bars, 5 µm. (**C**) YFP: TIO enrichment orthogonal to the cell plate direction in wild-type (green) or *kin-12f* background (pink). Two-way ANOVA and post-hoc Tukey test showed no significant difference (*P* > 0.05). *N* = 35 (WT control) and 45 (kin-12f). (**D**, **E**) CLSM sections of primary root epidermal cells expressing GFP: MAP65-3 in absence (**D**) or presence (**E**) of DEX»RAB-A2aNI expression counterstained with FM4-64. (**F**) Mean normalized intensity of GFP: MAP65-3 in axis perpendicular to cell plate in presence or absence of RAB-A2a[NI]. *N* = 79 (DMSO) and 135 (Dex). (**G**) Relative fluorescence intensity of GFP: MAP65-3 along cell plates as those shown in (**D**, **E**). Cell plates were grouped by diameter into short ( < 9 µm), medium (9–13 µm) and long ( > 13 µm). Lines are mean values, shaded areas are +/− 1 SD. *N* = 10 (DMSO long), 13 (Dex long), 30 (DMSO medium), 42 (DMSO short), 55 (Dex short), and 60 (Dex medium). Scale bars, 5 µm.

To test whether TIO kinase recruitment was also affected by Kin-12A/B and RAB-A2a activity during cytokinesis, we expressed YFP:TIO conditionally in stable *Arabidopsis* lines (*AtRPS5a»DEX»YFP:TIO*). Consistent with previous reports based on immunolocalization in cultured *Arabidopsis* cells (Oh et al, 2005), YFP:TIO localized to the phragmoplast midzone, and followed the expanding phragmoplast as cytokinesis progressed (Fig. 5J). Although YFP:TIO was still recruited to the phragmoplast midzone when Kin-12A/B or RAB-A2a function was perturbed, its pattern was significantly altered. In the *kin-12a/b* background, YFP:TIO intensity was significantly reduced at the midzone and instead was more broadly spread across the zone perpendicular to the midzone, corresponding to the area occupied by the phragmoplast (Fig. 5J,K,N). This change in TIO pattern did not occur in the *kin-12f* background (Fig. EV9A–C), consistent with the lack of evidence for interaction between Kin-12F and TIO. Additionally, YFP:TIO did not become more laterally diffuse in the presence of dominant-negative RAB-A2a[SN] or RAB-A2a[NI] (Fig. 5L–N), but instead, became patchy and significantly more variable along the length of the cell plate, and persisted at the centre of the cell plate at late stages (Fig. 5L,M,O,P). Such an increase in intensity variation did not occur for midzone-localized GFP:MAP65-3 in the presence of RAB-A2a[NI] (Fig. EV9D–G). This indicates that this change in YFP:TIO pattern does not reflect a generic midzone geometry or structure change caused by RAB-A2a inhibition, but is instead specific to TIO. Taken together, these data indicate that Kin-12A/B contribute to recruiting and confining TIO kinase to the phragmoplast midzone (probably alongside other reported TIO kinase interactors, Oh et al, 2014), while RAB-A2a contributes to TIO turnover during cell plate expansion.

### TIO kinase is required for phragmoplast formation and expansion in somatic cells

**Figure EV10:**
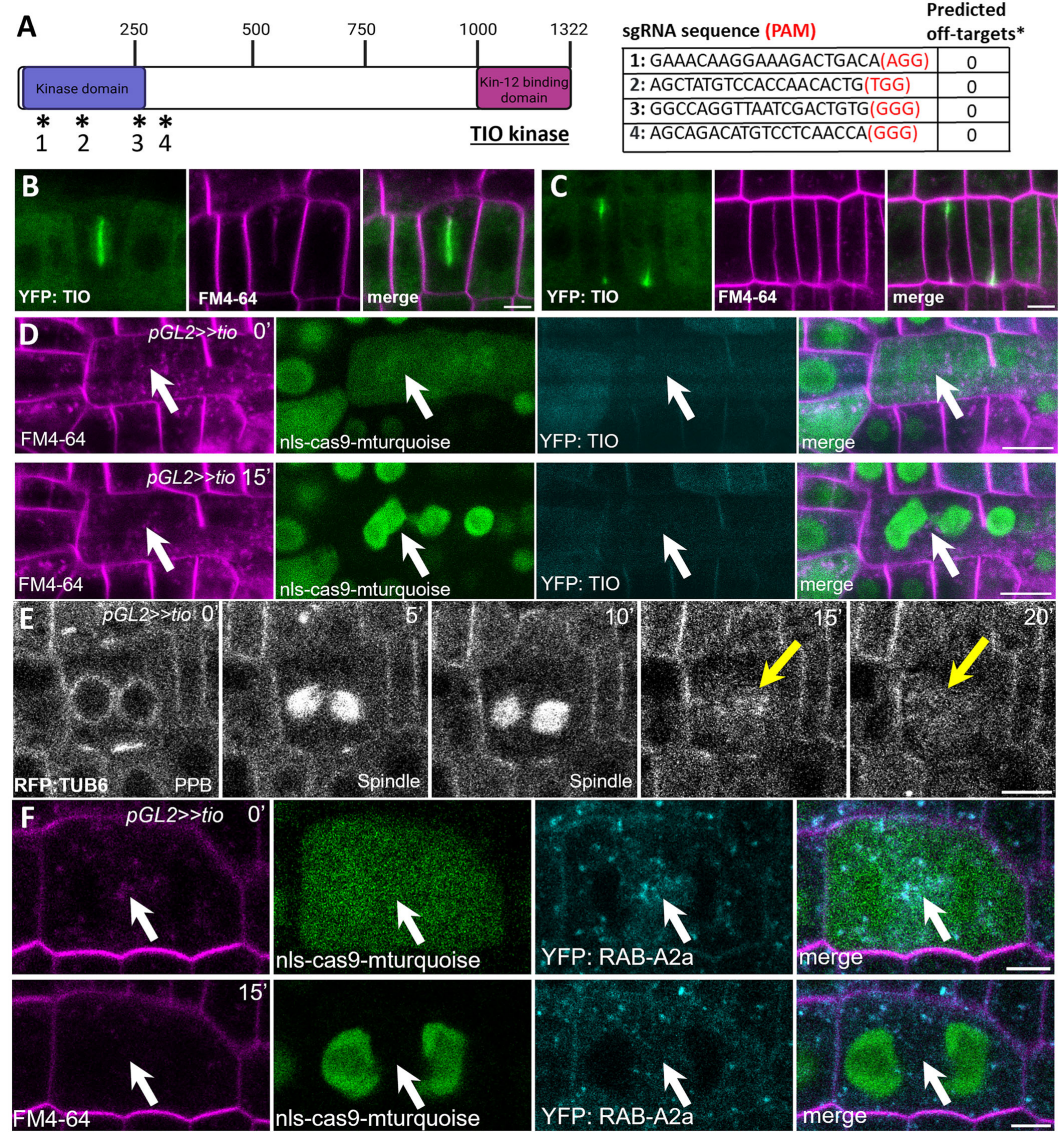
TIO is required for somatic cytokinesis during which it plays a role in phragmoplast formation. (**A**) Schematic of TIO kinase indicating positions of sgRNAs, and table indicating sgRNA sequences with predicted off-targets. sgRNA sequences in black, adjacent PAM site in red. *Off-targets as predicted by CHOPCHOP, which searches for sequences with up to 3 mismatches in the 20 bp sequence upstream of the PAM. 0 off-targets therefore means that no sequence matches were found that have 3 or fewer mismatches with the sgRNA sequence. (**B**, **C**) CLSM sections of primary root epidermal meristematic cells expressing DEX»YFP: TIO counterstained with FM4-64 during early-mid (**B**) and late (**C**) cytokinesis. (**D**) CLSM sections of primary root epidermal meristematic cells co-expressing *pGL2::tio::cas9-nls-mturquoise* (*pGL2»tio)* and DEX»YFP: TIO before (0 min) and after (15 min) nuclear reformation during cell division. White arrow indicates midzone position between future nuclei. (**E**) Sequential CLSM sections of primary root epidermal meristematic cells co-expressing *pGL2»tio* and RFP: TUB6. Yellow arrow indicates amorphous mass of microtubules at cell centre after spindle dissolution. (**F**) CLSM sections of primary root epidermal meristematic cells co-expressing *pGL2»tio* and YFP: RAB-A2a before (0 min) and after (15 min) nuclear reformation during cell division. White arrow indicates midzone position between future nuclei. Note the clustering of compartments at the midzone at 0 min but not at 15 min. Scale bar, 5 µm.

**Figure 6:**
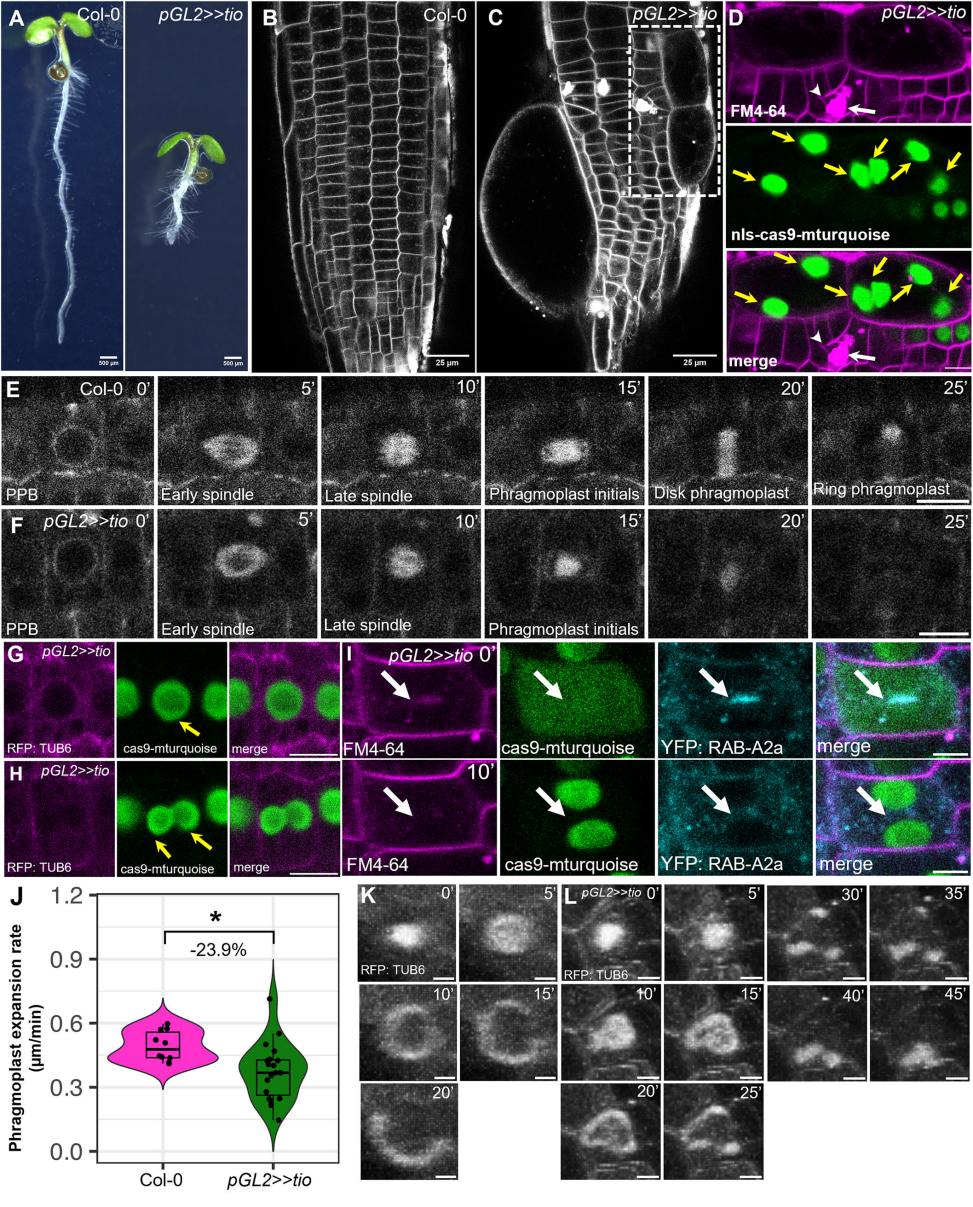
TIO kinase functions upstream of phragmoplast formation and expansion in somatic cells. (**A**) Brightfield images of Col-0 and * pGL2::tio::cas9-nls-mturquoise (pGL2»tio)* 5-day-old T1 seedlings. (**B**, **C**) CLSM section of primary roots stained with FM4-64 from plants such as those shown in (**A**). (**D**) CLSM section of primary root meristematic epidermal cells in the *pGL2»tio* background counterstained with FM4-64. White arrowhead indicates incomplete crosswalls, white arrow indicates amorphous structures of unknown identity, yellow arrows indicate nuclei (cas9 expression) in multinucleate cells. Note that this is  the section indicated in Fig. 6C. (**E**, **F**) CLSM sections of primary root meristematic epidermal cells of Col-0 background (**E**) or *pGL2»tio* background (**F**) expressing *RFP: TUB6*. Imaged at 5 min intervals. (**G**, **H**) CLSM sections of primary root meristematic epidermal cells of *pGL2»tio* background before (**G**) and after (**H**) mitosis. (**I**) CLSM sections of primary root epidermal meristematic cells co-expressing *pGL2»tio* and YFP: RAB-A2a before (0 min) and after (10 min) nuclear reformation during cytokinesis. White arrow indicates midzone position between future nuclei. Scale bar, 5 µm. (**J**) Violin plots of phragmoplast expansion rates in primary root epidermal meristematic cells in Col-0 (*RFP: TUB6*) and *RFP: TUB6 pGL2»tio* backgrounds. Expansion rate was significantly reduced in the *RFP: TUB6 pGL2»tio* background (*P* = 0.0153787, Student’s *T* test) *n* = 10 (Col-0), 19 *(pGL2»tio*). In box plots, the median is displayed as a line, lower and upper hinges correspond to the 25th and 75th percentiles, the lower and upper whiskers extend from the hinge to the smallest or largest value no further than 1.5 * IQR from the hinge. (**K**, **L**) CSLM maximum intensity projections of orthogonally resliced images of primary root meristematic epidermal cell expressing *RFP:* TUB6 in a Col-0 WT or *pGL2»tio* background. Images taken at 5 min intervals. Note that phragmoplast morphology is abnormal and fragments (**L**) prior to reaching the cell surface. Scale bar, 5 µm. Source data are available online for this figure.

We next aimed to functionally test the role of TIO in somatic cytokinesis, as previous work identifying it as a positive regulator of phragmoplast expansion was performed in microspores (Oh et al, 2012). Homozygous knockout mutants of TIO kinase have previously been shown to be unobtainable due to germline sterility (Oh et al, 2005, 2012). We therefore used CRISPR-Cas9 driven from the GL2 promoter to target TIO kinase in somatic cells (*pGL2::tio::cas9-nls-mturquoise;* hereafter *pGL2»tio*, Fig. EV10A). *pGL2»tio* was able to efficiently knock out TIO kinase when co-expressed with conditionally-induced YFP:TIO (Fig. EV10B–D). In the T1 generation of all backgrounds in which we expressed *pGL2»tio*, we recovered plants that were extremely stunted (Fig. 6A). These plants had numerous cytokinesis defects in their primary roots, including the presence of incomplete cross-walls in some cells, as well as large, multinucleate cells that showed no signs of having undergone cytokinesis (Fig. 6B–D). As we had previously observed in *pGL2»kin-12f* plants, cytokinetic failure in *pGL2»tio* plants was also, albeit less frequently, accompanied by the appearance of amorphous structures (Fig. 6C,D).

We next used *pGL2»tio* in the RFP:TUB6 background to monitor the effect of TIO suppression on phragmoplast dynamics. Surprisingly, we observed that of 45 nuclear divisions imaged in RFP:TUB6 *pGL2»tio* primary root epidermal cells, 20 ended with complete failure to form a recognisable phragmoplast following spindle dissolution (Fig. 6E,F). In these cells, we observed phragmoplast initials, but these failed to form into a disk phragmoplast, instead often dissolving into amorphous tubulin networks at the cell centre, resulting in bi- or multinucleate cells (Fig. 6G,H and EV10E; Movie EV2). Correspondingly, we only infrequently observed YFP:RAB-A2a and FM4-64 accumulation at the midzone in such cells, which was limited to clustering of compartments around the midzone or very early stage cell plates that failed to progress into a late stage cell plate or crosswall (Figs. 6I and EV10F). Taken together this suggests that the earliest stage of phragmoplast formation and vesicle transport to the midzone (phragmoplast initials) is retained in cells expressing *pGL2»tio*, but frequently fails to progress beyond this (Fig. 6F).

TIO kinase has previously been described as an upstream regulator of phragmoplast expansion of *Arabidopsis* microspores (Oh et al, 2012). To test whether phragmoplast expansion was also affected in somatic cells, we quantified the expansion rates of the ~55% of phragmoplasts that did successfully form in primary root epidermal cells of *pGL2»tio RFP:TUB6* lines. We found that the expansion rate of these phragmoplasts was significantly reduced compared to those in wild-type controls (Fig. 6J), and phragmoplasts often had unusual shapes and fragmentary expansion patterns (Fig. 6K,L), corresponding to the incomplete divisions we observed in *pGL2»tio* lines. The most parsimonious explanation for our observations and previously published data (Oh et al, 2012) is that TIO kinase acts in a fundamental process in microtubule organization that is required both in phragmoplast formation and expansion.

Taken together, our experimental and in silico observations are consistent with a scenario in which RAB-A2a binding aids TIO recruitment to Kin-12A/B at the midzone, possibly through inducing a conformational change in the kinesin tail region, and/or through promoting recruitment of TIO kinase via forming a direct binding interface with it. We propose that this triple interaction acts to couple vesicle delivery and proper formation and expansion of the phragmoplast during cytokinesis.

## Discussion

**Figure 7:**
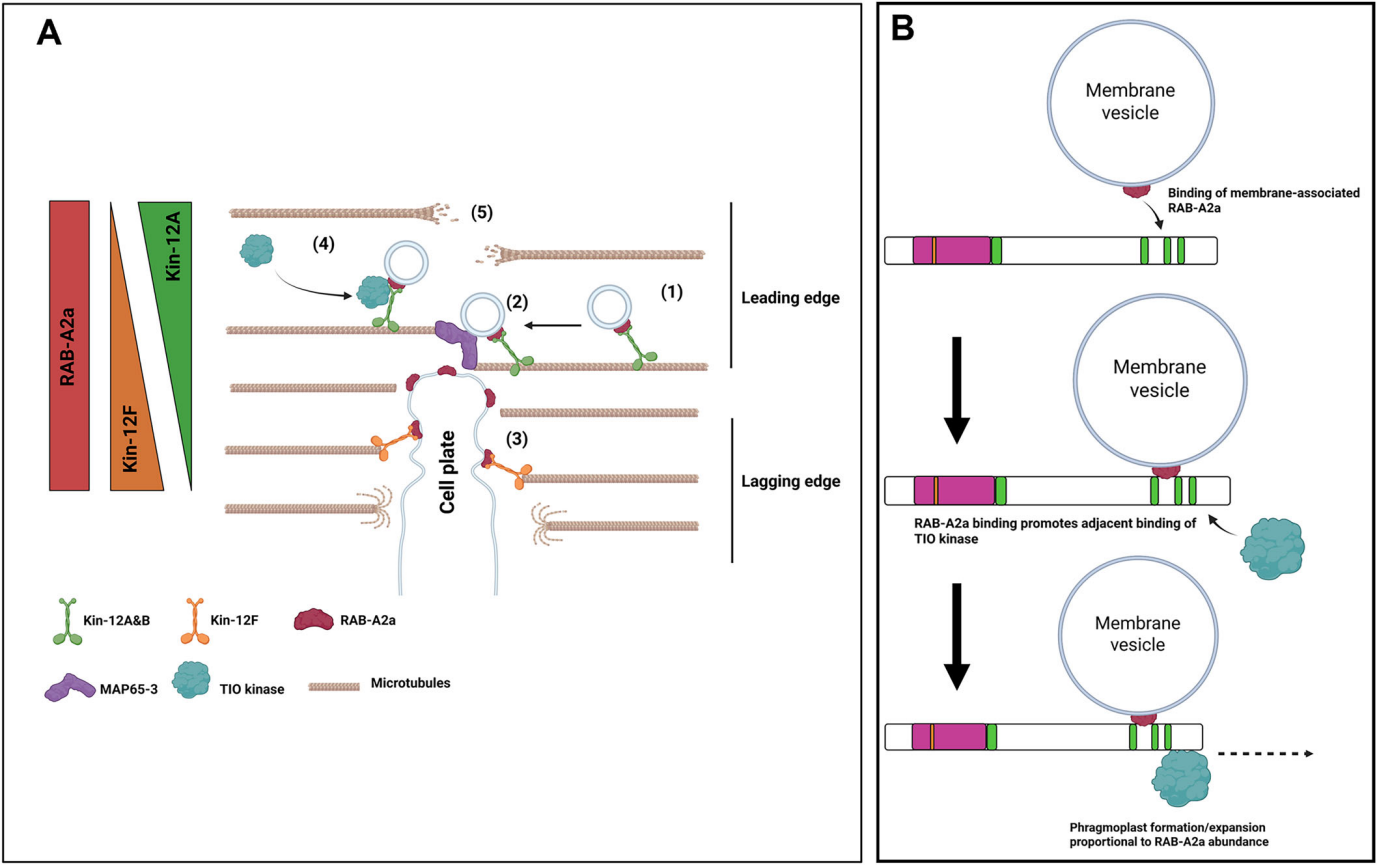
Model for Class II RAB-A2a/Kin-12/TIO module function during cytokinesis. (**A**) (1) Transport of RAB-A2a-bearing vesicles to the midzone is driven by kinesins that include Kin-12A&B. (2) Kin-12A (and likely Kin-12B) arrives at the leading edge midzone where it is stabilized by the microtubule crosslinker MAP65-3, positioning RAB-A2a-bearing vesicles for fusion. (3) Non-motile Kin-12F localizes to the lagging edge independently of MAP65-3 function and provides an additional platform to support the formation of the tubulo-vesicular cell plate (4) RAB-A2a activity and Kin-12A&B dictate proper TIO kinase localization at the phragmoplast midzone, which (5) couples phragmoplast formation and expansion to membrane presence at the midzone. (**B**) Model for recruitment of RAB-A2a and TIO kinase to the midzone. Binding of membrane-associated RAB-A2a to the Kin-12A&B tail regions promotes binding of TIO kinase to the adjacent part of the tail regions, possibly through inducing a steric change in the kinesin tail region. TIO kinase activity at the midzone then promotes phragmoplast formation and expansion proportional to RAB-A2a abundance at the midzone.

In this study, we investigate the function of RAB-A2a, Class II Kin-12s, and TIO Kinase during cytokinesis. With the exception of Kin-12F, all these proteins were already known to play a role in cytokinesis. However, due to our new in silico, in vitro, and in vivo data, we are now able to propose a mechanistic model for how these proteins collectively function to couple membrane targeting and phragmoplast expansion during cytokinesis (Fig. 7).

**(1)** RAB-A2a-bearing vesicles are brought to the midzone along phragmoplast microtubules by transport kinesins, which include Kin-12A/B. In the absence of all three Class II Kin-12s, we observed membrane targeting defects during somatic cell cytokinesis in the form of incomplete or absent crosswalls, large amorphous structures and reduced RAB-A2a abundance at the cell plate. Our data indicate that these defects likely arise from combined loss of different roles played by Class II Kin-12s. Both Kin-12A/B were motile in in vitro gliding assays, indicating that they may play a role in vesicle transport to the midzone. This is consistent with observations for homologous kinesins in *Physcomitrium* (Yamada et al, 2025). However, we did not observe such motility for Kin-12F, which lacks key amino acid motifs associated with kinesin motility (Parke et al, 2010; Cross and McAinsh, 2014). Furthermore, Kin-12A, but not Kin-12F accumulated at the phragmoplast leading edge, the main site of vesicle delivery. **(2)** Kin-12A/B, downstream of the microtubule-crosslinker MAP65-3, platform vesicles at the leading edge, where vesicle tethering and fusion is initiated. Kin-12A localization was previously shown to depend on the interdigitating microtubule crosslinker MAP65-3, which participates in anchoring of microtubules at the leading edge. **(3)** Kin-12F participates in microtubule-membrane interactions at the cell plate lagging edge to promote formation of a tubulo-vesicular network and a stable cell plate. In contrast to Kin-12A, Kin-12F was present at the lagging edge where the cell plate becomes a tubulo-vesicular network and the majority of microtubules attach to the cell plate without the participation of the interdigitating microtubule crosslinker MAP65-3. Consistent with this, we found that localization of Kin-12F to the midzone was not dependent on MAP65-3. This localization is also consistent with the apparent lack of motile behaviour in Kin-12F, and we propose that it may primarily act to stabilize membrane-microtubule interactions during the formation of the cell plate tubulo-vesicular network, after the initial vesicle delivery to the phragmoplast leading edge. In this scenario, Kin-12F may attach from lagging edge zone microtubule plus ends to RAB-A2a positive membranes that have already transitioned from delivered vesicles into larger compartments, keeping these in position while they fuse into a tubulo-vesicular network. This would imply that cytokinesis defects in the absence of all Class II Kin-12s arise from combined effect of defects in vesicle transport to the midzone, and subsequent defects in membrane-microtubule interactions at the midzone after vesicle delivery, resulting in a situation in which mistargeted vesicles fuse to form membrane structures away from the midzone, preventing complete cytokinesis. Notably, although YFP:RAB-A2a is partially mistargeted and its abundance at the cell plate is reduced upon loss of all three Class II Kin-12s, it is not entirely abolished from the cell plate. One explanation for this is that RAB-A2a may be transported to the cell plate by other kinesins in addition to Class II Kin-12s, either through direct interactions, or passively on TGN/EE membranes that associate with other kinesins by different means. Indeed, as total failure to form a cell plate (corresponding to a complete lack of a visible crosswall) was only occasionally seen in our Class II Kin-12 mutants, it is likely that other kinesins do function in vesicle targeting to the midzone during plant cell cytokinesis. Additionally, RAB-A2a may also be directly recruited from the cytosol to cell plate membranes independently of kinesin-based transport from the TGN/EE. Indeed, upstream regulators of RAB-A2a such as the TRAPPII complex are known to localize to the cell plate (Rybak et al, 2014; Kalde et al, 2019) and could provide a kinesin-independent means of RAB-A2a recruitment. **(4)** The interaction of RAB-A2a with the tail regions of Kin-12A/B promotes recruitment of TIO kinase. Our in silico and experimental data demonstrate that RAB-A2a and TIO kinase interact with Kin-12A/B in adjacent, but non-overlapping domains. AF3 predictions suggest that RAB-A2a binding to Kin-12A stabilises a more open conformation of the kinesin tail region, which may enhance TIO kinase binding. This mechanism could then explain our experimental data demonstrating that functional RAB-A2a can enhance the TIO/Kin-12A interaction in vivo. During cytokinesis, loss of either Kin-12A/B or RAB-A2a caused defects in TIO patterning, although these were distinct from each other and neither completely abolished TIO from the phragmoplast midzone. In *kin-12a/b*, TIO was more diffuse and less well focused on the midzone, consistent with the loss of direct binding partners patterning it to this domain. By contrast, when we overexpressed dominant-negative RAB-A2a variants, TIO patterning on the cell plate became more variable and TIO was not efficiently removed from the centre of the expanding cell plate, suggesting defects in recruitment and turnover. These observations are consistent with a model where RAB-A2a presence at the midzone enhances regulatory interactions between TIO and Kin-12A/B, which are essential for cytokinesis. **(5)** TIO activity promotes microtubule organization, allowing first phragmoplast formation, and later phragmoplast expansion. When we knocked out TIO kinase in atrichoblast cells via CRISPR-Cas9, ~45% of dividing cells completely failed to form disk phragmoplasts from phragmoplast initials. In the remaining cases of division where phragmoplast formation was successful, we found that phragmoplast expansion rates were reduced, consistent with previous observations in microspores (Oh et al, 2005). The most direct explanation for this would be that TIO activity is required in somatic cells for a fundamental process in phragmoplast microtubule organization that is required for the microtubule re-arrangement that underpins both phragmoplast formation and expansion beyond the initials phase. It is therefore possible that failure to form phragmoplasts also occurs in *tio* microspores but has not been detected without time-lapse imaging of cytokinesis, or alternatively, that the role of TIO kinase differs between cytokinesis in different cell types. As substrates of TIO kinase have not been identified, it is not known exactly how it drives phragmoplast formation and expansion. However, both the predicted active kinase residues of TIO, as well as residues required for TIO binding to Kin-12A/B, are necessary to rescue the pollen phenotype of *tio* hemizygous mutants (Oh et al, 2014). Moreover, it has recently been shown that the chemical compound PP2 blocks phragmoplast formation in a manner strikingly similar to *pGL2»tio* mutants. The authors identified that PP2 treatment disrupted phosphorylation of Kin-12A, and proposed that PP2 may block phragmoplast formation by inhibiting phosphorylation of Class II Kin-12s (Kimata et al, 2023). Combining these observations with those presented here, it is tempting to speculate that PP2 treatment may block TIO kinase activity and that Class II Kin-12s may themselves be substrates of TIO kinase phosphorylation activity. Indeed, recent data suggest that a Kin-12A variant with reduced phosphorylation is excessively stabilized on microtubules (Kimata et al, 2023), which could be inhibitory to microtubule turnover required for phragmoplast growth. Additionally, Kin-12A/B are first recruited to the midzone during the phragmoplast initials stage (Pan et al, 2004), the same stage at which phragmoplast defects first occurred in *pGL2»tio* plants. However, the fact that we observed total failure to form phragmoplasts far more frequently upon loss of TIO kinase than upon loss of Class II Kin-12s supports a scenario in which other targets of TIO kinase exist at the initials stage that contribute to phragmoplast formation and expansion. At least one other potential interactor of TIO kinase, the kinesin TETRASPORE, has been reported previously (Oh et al, 2014). While we could find no evidence that Kin-12F interacts with or positions TIO kinase, we also cannot formally exclude the possibility that is either a transient direct target or an indirect target of TIO kinase. This, or Kin-12F playing a TIO-independent role in microtubule organization at the midzone, could explain our observation that the *kin-12f* mutant itself has a mild reduction in phragmoplast expansion rates.

Our model, while not yet definitive, provides a mechanistic explanation for previous observations regarding the coupling of vesicle delivery and phragmoplast growth (Yasuhara and Shibaoka, 2000; Steiner et al, 2016; Lin et al, 2019), and could explain how plants ensure uniform cell plate morphology during cytokinesis.

### Remaining open questions

- Can enhanced interaction of TIO kinase with Kin-12A/B upon binding of RAB-A2a be demonstrated in vitro*?*
- What are the molecular substrates of TIO kinase activity?
- How does TIO-dependent phosphorylation of its substrates promote phragmoplast formation and expansion?
- In addition to Class II Kin-12s, what other kinesins transport vesicle to the midzone during cytokinesis in plant cells?
- What underpins the different molecular requirements of cytokinesis in somatic and germline cells of plants?

## Methods

**Table:** Reagents and tools table

Reagent/resource	Reference or source	Identifier or catalog number
**Experimental models**		
*Arabidopsis thaliana*		
*Nicotiana benthamiana*		
*Saccharomyces cerevisiae*		
**Recombinant DNA**		
pBiFCt-2in1-NN	Grefen and Blatt, 2012. BioTechniques.	Addgene #105111
pDONR221-P1P4	Thermofisher	
pDONR221-P3P2	Thermofisher	
pDONR207	Thermofisher	
pOpIN2-RPS5A	NASC. Samalova et al, 2019 Current Protocols in Plant Biology	NASC ID N2109492
pUB-DEST	Grefen et al, 2010	
pET-23c	Novagen	
Numerous Golden Gate vectors (see “Methods”)	Ordon et al, 2017; Weber et al, 2011; Castel et al, 2019	
**Antibodies**		
Anti-Kin-12F polyclonal	This study, Biosynth Laboratories	
Anti-RAB-A2a polyclonal	Chow et al, 2008	
anti-tubulin YL-1/2 monoclonal	Sigma/Merck	MAB1864-I
CY-3 AffiniPure polyclonal	Jackson Immunoresearch	AB_2338000
Anti-GFP rabbit polyclonal	Abcam	Abcam 290
Anti-rabbit AP-coupled secondary	Sigma/Merck	AP132A
**Oligonucleotides and other sequence-based reagents**		
PCR primers	This study (IDT Technologies)	Table EV1
Synthesized Kin-12A and RAB-A2a substituted DNA templates	IDT Technologies	
**Chemicals, enzymes and other reagents**		
Diverse restriction enzymes (see “Methods”)	New England Biolabs	
T4 DNA ligase	Thermofisher	EL0011
Dexamethasone	Sigma/Merck	D4902-25MG
Brefeldin-A	Sigma/Merck	B7651-5MG
Perfluorodecalin	Sigma/Merck	P9900-25G
**Software**		
FIJI	Fiji Downloads	
AlphaFold3	AlphaFold Server	
PISA	Krissinel and Henrick, 2007	
GROMACS	Abraham et al, 2015	
Mol* Viewer	Sehnal et al, 2021	
CHOPCHOP	https://chopchop.cbu.uib.no/	
**Other**		
µMACS anti-GFP magnetic beads	Miltenyi Biotec	130-094-252
µMACS columns	Miltenyi Biotec	130-042-701
DNA sequencing	Microsynth France	

### Plant materials, growth and treatments

The *Arabidopsis thaliana* ecotype Columbia was used throughout with the exception of the *dyc283* mutant of MAP65-3, which is in Wassilewskija ecotype (Caillaud et al, 2008). *YFP: Kin-12F dyc283* plants described in Fig. 4 are therefore Columbia - Wassilewskija hybrids. Kin-12A is denoted as At4g14150 in the *Arabidopsis* genome, Kin-12B as At3g23670, and Kin-12F as At3g20150. The transgenic lines *pRAB-A2a::YFP:RAB-A2a* (Chow et al, 2008), p*35S»DEX»RAB-A2a[SN]* (Chow et al, 2008), *p35s::mCherry:RAB-A2*a (Kirchhelle et al, 2016), *kinesin12a/b* (Lee et al, 2007), *pUBQ10::RFP:TUB6* (Ambrose et al, 2011) and *pMAP65-3::GFP:MAP65-3* (Caillaud et al, 2008) have all been described before. The *kinesin-12f* SALK insertion line described here is SALK_039654C (NASC ID N655459). All plants were grown at 20 °C in a 16 h:8 h day:night cycle. Primary roots were imaged 3–5 days after germination on half-strength Murashige and Skoog medium (MS, Sigma-Aldrich) plates with 1% w/v sucrose and 0.8% Difco-agar (Appleton Woods) at pH 5.7. For constructs whose expression was induced conditionally via dexamethasone (Dex), plants were grown for 2.5 days from germination on half-strength solid MS medium before transfer to liquid half-strength MS medium (1% w/v sucrose, pH 5.7) for the indicated time period containing either Dex (Sigma-Aldrich—diluted from a 20 mM stock in DMSO) at the indicated concentration, or the equivalent concentration of DMSO. Plants in liquid half-strength MS were gently agitated on an orbital shaker at 50 rpm throughout the induction period. Treatment with Brefeldin-A (Sigma-Aldrich) was performed in water for the indicated time period with 50 µM Brefeldin-A (diluted from a 50 mM stock in DMSO) or the equivalent concentration of DMSO. For GUS staining, plants were incubated in a solution of 1 mM X-Gluc, 0.05% Triton X-100, 5 mM EDTA, 50 mM sodium phosphate, 0.5 mM potassium ferrocyanide and 0.5 mM potassium ferricyanide for 1 h at 37 °C. Staining of primary roots was then photographed directly. For staining with FM4-64 (Thermo Fisher Scientific), plants were incubated for 15 min in a solution of 0.1 µg/mL prior to imaging (diluted from a 1 mg/mL stock in water). Fixation and co-staining of primary roots with calcofluor white (Fluorescent Brightener 28 disodium salt solution, Sigma-Aldrich) and propidium iodide (Sigma-Aldrich) was performed as previously described (Chow et al, 2008). Transformation of the novel transgenes described in this study into plants was performed *via Agrobacterium*-mediated floral dip (Clough and Bent, 1998).

### Molecular cloning

All genes were amplified by PCR with Phusion^TM^ High-Fidelity DNA Polymerase (Thermo Fisher Scientific) from cDNA of *Arabidopsis* ecotype Columbia. *DEX»RPS5a»YFP: Kinesin-12F, DEX»YFP: Kinesin-12F Tail* and *DEX»RPS5a»YFP-TIO* were all generated by cloning of the relevant cDNA region into a YFP-containing pENTRY vector via digestion with the restriction endonuclease AscI (New England Biolabs). The resulting YFP-fusion transgenes were then transferred into *pOpIN2-RPS5a* (Samalova et al, 2019) using Gateway^TM^ LR Clonase II Enzyme Mix (Thermo Fisher Scientific). For *DEX»RPS5a»mClover: Kinesin-12A*, mClover: Kinesin-12A was amplified from a pre-existing vector (Kimata et al, 2023), cloned into the Gateway entry vector pDONR207 using Gateway^TM^ LR BP Enzyme Mix (Thermo Fisher Scientific), and then into *pOpIN2-RPS5a* (Samalova et al, 2019) using Gateway^TM^ LR Clonase II Enzyme Mix (Thermo Fisher Scientific). For *DEX»RPS5a»RAB-A2a* and its mutant variants, the RAB-A2a wild-type or mutant variants were amplified from pre-existing templates using gateway primers and cloned in the same way as *mClover: Kinesin-12A*. Tail regions of Kinesin-12A, Kinesin-12B and Kinesin-12F (and truncations of them) used in pairwise Y2H tests were amplified from cDNA with primers containing EcoRI and XhoI restriction endonuclease digest sites and cloned into the vector pJET1.2/blunt (CloneJET, Thermo Fischer Scientific). In the cases of Kinesin-12B and Kinesin-12F, an internal EcoRI site in each was first removed by PCR. The tail regions were excised from pJET1.2/blunt via digestion with EcoRI and XhoI (New England Biolabs) and ligated into the activation-domain vector pAD-Gal4 (Stratagene) using T4 DNA ligase (Thermo Fisher Scientific). Rab GTPases and mutant variants of them used in pairwise Y2H tests were amplified from cDNA or pre-existing templates with primers containing KpnI and SmaI restriction endonuclease digest sites, and cloned into the binding-domain vector pLexA-C (Clonetech), also via the intermediate vector pJET1.2/blunt (CloneJET, Thermo Fischer Scientific). All cloned Rab GTPase variants used for Y2H lack the DNA sequence for the final 6 amino acids so as to prevent geranylgeranylation in yeast. For pairwise Y2H tests between Kinesin-12 tail regions and TIO kinase, pGADT7 and pGBKT7 (Clonetech) containing Kinesin-12A&B tail regions and TIO kinase respectively have been described before (Oh et al, 2012). For cloning of Kinesin-12F tail regions into pGADT7, Kinesin-12F tail region truncations were amplified from cDNA with primers containing XmaI and XhoI digest sites and introduced into pGEM®-T Easy (Promega). These were then removed via digestion with XmaI and XhoI (New England Biolabs) and ligated into XhoI/XmaI-digested pGADT7 using T4 DNA ligase (Thermo Fisher Scientific).

For CRISPR-Cas9 targeting of Kinesin-12F and TIO, four target sites for each gene were identified using ChopChop (https://chopchop.cbu.uib.no/). Target sites were incorporated into two complementary oligonucleotides prefaced with the sequences *attg* and *aaac*, respectively. Each oligonucleotide pair was then hybridized via denaturing at 98 °C for 5 min and subsequent incubation at room temperature for 5 min. Hybridized oligonucleotides were then incorporated into plasmids pDGE5, pDGE7, pDGE9 and pDGE11, respectively (Ordon et al, 2017) via a Golden Gate reaction with the type-IIS restriction endonuclease BpiI (New England Biolabs). These were then simultaneously transferred into a single vector via a Golden Gate level 1 reaction with BsaI (New England Biolabs) and a modified version of pICH47742 (Weber et al, 2011). For tissue-specific CRISPR from the *GLABRA2* promoter, the final constructs were assembled via a Golden Gate level 2 reaction between pICH47732-FAST-Red (Position 1 (Castel et al, 2019)), pICH47742-oligonucleotides (Position 2), pICH47751-pGL2::zCas9iNLs-2AmturquoiseN7-rnsCE9t (Position 3), pICH41766 (Position 4 (Weber et al, 2011)) and pAGM4673 (Weber et al, 2011) using BpiI.

For rBiFC vectors containing split-YFP variants of Kin-12A tail, -12F tail and the TIO C-terminus, these regions were amplified from *Arabidopsis* cDNA and introduced into the Gateway vectors pDONR221-P3P2 (-12A tail, -12F tail) and pDONR221-P1P4 (TIO C-terminus) using BP Clonase II enzyme mix (Invitrogen/Thermo Fischer Scientific). These were subsequently simultaneously introduced into pBiFCt-2in1-NNGrefen and Blatt, 2012 using LR clonase enzyme mix (Thermo Fischer Scientific). RAB-A2a variants co-expressed in *Nicotiana* with rBiFC constructs were cloned from pre-existing templates into the Gateway vector pDONR207 (Invitrogen/Thermo Fischer Scientific) with BP clonase II enzyme mix, and subsequently into pUB-DEST (Grefen et al, 2010) with LR clonase enzyme mix. The Kin-12A and RAB-A2a variants with binding interface substitutions were cloned in this manner from synthesized templates (Integrated DNA Technologies).

All primer sequences used in this study can be found in Table EV1.

All constructs described in this study were validated via Sanger sequencing (Microsynth) and restriction digests. *Escherichia coli* strains DH5α and DB3.1 were used for molecular cloning. Constructs were introduced into the *Agrobacterium tumefaciens* strain GV3101::pMP90 by electroporation prior to transformation of *Arabidopsis*.

### Yeast two-hybrid

For the initial Y2H screen and subsequent pairwise Y2H tests between Kinesin-12 members and Rab GTPases, the haploid NYM51 and NMY61 (Dualsystems Biotech) strains of *Saccharomyces cerevisiae* were used. For the screen, NYM61 cells were transformed with a Gal4-AD-fused *Arabidopsis* cDNA library via lithium acetate-mediated transformation (Gietz and Woods, 2001), and subsequently mated with NMY51-expressing pLexA-C RAB-A2aQL. Subsequent selection conditions for positive interactions were as previously described (Camacho et al, 2009). For later confirmation via independent pairwise Y2H tests, NYM51 was transformed with pLexA-C containing Rab GTPase variants, NMY51 was transformed with pAD-Gal4 containing Kinesin-12 tail regions (see above) and transformants selected on SD-Leu or SD-Tryp medium. Diploids were obtained via mating on YPDA medium and subsequent selection on SD-Leu-Tryp medium. For pairwise Y2H tests between Kinesin-12 members and TIO kinase, the diploid strain AH109 (Clonetech) was used to mimic the original conditions identifying this interaction (Oh et al, 2012). AH109 was simultaneously transformed with pGADT7-containing Kinesin-12 tail truncations and pGBKT7-containing TIO kinase truncations via lithium acetate-mediated transformation (Gietz and Woods 2001), and dual transformants selected on SD-Leu-Tryp medium. For all interaction tests displayed, diploids were grown in liquid SD-Leu-Tryp medium overnight at 30 °C. Cultures were diluted to O.D_600_ of 1, 0.1 and 0.01 in sterile MilliQ water before plating onto SD-Leu-Tryp and SD-Leu-Tryp-His-Ade plates with a multichannel pipette. Plates were then incubated between 2 and 4 days at 30 °C. All pairwise Y2H tests were performed independently at least three times, and a representative result is shown.

### Co-IPs and immunoblots

For testing co-immunoprecipitation between YFP: Kinesin-12F/YFP: Kinesin-12F-tail and mCh:RAB-A2a, a method adapted from that described in (Kalde et al, 2019) was used. Briefly, plants were grown in liquid ⅓-strength MS (1% w/v sucrose, pH 5.7) for 7 days with gentle agitation of 50 rpm on an orbital shaker. At 7 days, 20 µm Dex was added for 16 h. Root and shoot tissue were then harvested and homogenized in an ice-cold isolation buffer with 2% w/v CHAPS (Sigma-Aldrich). The homogenate was then centrifuged at 14,000 rpm for 5 min at 4 °C. The supernatant was removed and 1 mM DTSSP added, then incubated on ice for 15 min. DTSSP activity was then quenched by addition of 50 mM Tris-HCl pH 7.5, and a further 5 min incubation on ice. The mixture was then centrifuged at 14,000 rpm for 10 min at 4 °C. In total, 50 µl of µMACS anti-GFP magnetic beads (Miltenyi Biotec) were added to the supernatant, and this was then incubated for a further 30 min on ice. Bead complexes were then washed and isolated using µMACS columns (Miltenyi Biotec) as previously described (Kalde et al, 2019). Immunoblots against YFP: Kinesin-12F and RAB-A2a used the primary antibodies anti-GFP ab290 (Abcam) at 1:5000 dilution, and anti-RAB-A2a (Chow et al, 2008) (at 1:1000 dilution, respectively. Alkaline-phosphatase-coupled goat anti-rabbit secondary antibody (Sigma-Aldrich) and Western Blue stabilized substrate (Promega) were used to visualize blotted proteins. For immunoblots against endogenous Kin-12F, crude protein extracts from 7-day-old *Arabidopsis* seedlings were used. Extracts were probed on western blots with anti-kin-12F polyclonal antibody at 1:1000 concentration (or equivalent concentration of pre-immune serum or immune-depleted serum). Blotted proteins were then visualized using goat anti-rabbit HRP secondary antibody (Thermo Fischer Scientific) at dilution 1:900 and ECL Western HRP substrate (Merck Millipore).

### Generation of anti-Kin-12F antibody

The polyclonal antibody was raised against the peptide sequence APPQNPNIHNPRNQSV, which is specific to the N-terminus of Kin-12F. This peptide was synthesized as [C]-APPQNPNIHNPRNQSV-amide, and subsequently used for immunization of two rabbits and ELISA tests (Biosynth Laboratories). Antisera were derived from 50 mL harvest bleeds. The crude antisera was purified by affinity chromatography on Thiopropyl Sepharose 6B coupled with the peptide antigen (Biosynth Laboratories). Bound antibodies were eluted in Glycine buffer (100 mM, pH 2.5).

### Immunofluorescence

Roots were fixed and stained with antibodies and DAPI following a previously published procedure (Smertenko et al, 2008). The immuno-depleted anti-Kin12F was prepared by incubating the serum diluted 1:500 with the recombinant Kin-12F antigen at final concentration 0.2 μg/ml for 30 min at room temperature. For staining, the anti-Kin-12F antibody was diluted in 1× PBS supplemented with 0.5% (w/v) BSA at 1:100 dilution. Roots were then incubated with this antibody solution or immunodepleted solution overnight at 4 °C, washed three times for 1 h each with 1× PBS at room temperature, and subsequently incubated with rat monoclonal anti-tubulin YL-1/2 antibody (Sigma-Aldrich) at 1:100 dilution. The anti-tubulin was washed out as above, and the roots were incubated overnight at 4 °C with a mixture of secondary anti-rabbit conjugated with CY3 and anti-rat conjugated with CY2 (Jackson ImmunoResearch) each diluted to final concentration 1:500. The secondary antibodies were washed out as above except that the second wash contained 50 ng/ml of DAPI to stain the DNA.

### Microtubule gliding and single molecule motility assays

The motor domains of Kin-12A, -12B and -12F were cloned from the *Arabidopsis* cDNA and integrated into a vector carrying sfGFP and 6×His sequences (pET-23c backbone). Expression of the proteins was induced in SoluBL21 E. coli with 0.2 mM IPTG for 20 h at 18 °C. Harvested cells were lysed using the Advanced Digital Sonifier D450 (Branson) in lysis buffer (25 mM MOPS [pH 7.0], 250 mM KCl, 2 mM MgCl2, 1 mM EGTA, 20 mM imidazole, 0.1 mM ATP) supplemented with 5 mM β-mercaptoethanol, 500 U benzonase and protease inhibitors (1 mM PMSF and peptide inhibitor cocktail: 5 mg/mL aprotinin, 5 mg/mL chymostatin, 5 mg/mL leupeptin, 5 mg/mL pepstatin A). After centrifugation, the clarified lysate was incubated with nickel-NTA coated beads for 1.5 h at 4 °C. Following three washes with the lysis buffer, proteins were eluted using 500 µl elution buffer (25 mM MOPS [pH 7.0], 75 mM KCl, 2 mM MgCl2, 1 mM EGTA, 200 mM imidazole, 0.2 mM ATP) supplemented with 5 mM β-mercaptoethanol. The elution was aliquoted after the addition of 20% (w/v) sucrose.

Microtubule polymerization was performed by preparing a tubulin mixture. This contained 70% pig tubulin and 30% Alexa Fluor 568-labeled pig tubulin in a total concentration of 100 µM with 0.5 mM GMPCPP, which was prepared in MRB80 (80 mM Pipes-KOH, pH6.8, 1 mM EGTA, 4 mM MgCl_2_). The tubulin mixture was incubated for 35 min at 37 °C to induce polymerization. For the gliding assay with purified kinesins, the method followed that previously described (Jonsson et al, 2015), and 1×Standard Assay Buffer (SAB: 25 mM MOPS [pH 7.0], 75 mM KCl, 2 mM MgCl_2_, 1 mM EGTA) was utilised. Briefly, 7 µL of the purified recombinant protein was introduced into a flow chamber and incubated at room temperature for 2 min in the dark. Subsequently, a 10 µL reaction mixture (1×SAB, 0.1% methylcellulose, 50 mM glucose, 0.5 µg/µL κ-casein, GMPCPP-stabilized microtubule seeds, oxygen scavenger system, 1 mM ATP) was introduced into the flow chamber, and it was sealed using candle wax. TIRF imaging of Alexa Fluor-568 labelled GMPCPP-stabilized microtubule seeds was performed every 3 s for 10 min at 23–25 °C. For the microtubule-binding and single molecule motility assay of the Kin-12F motor domain, a silanized coverslip was coated with an anti-biotin antibody, and 1×SAB buffer containing 1% pluronic acid was introduced into the chamber. After washing once with 1×SAB, GMPCPP-stabilized microtubules were loaded in the presence of 20 µM Taxol and incubated for 5 min. After washing once with 1× SAB supplemented with 20 µM Taxol, 10 µL of reaction -mixture (1×SAB, 0.1% methylcellulose, 50 mM glucose, 0.5 µg/µL κ-casein, 0.4 nM Kin-12F motor domain:GFP, 75 mM KCl, oxygen scavenger system, 1 mM ATP, 20 µM Taxol) was introduced into the flow chamber, and it was sealed using candle wax. TIRF imaging was performed every 1 s for 2 min at 23–25 °C.

### rBiFC

For rBiFC assays, *Agrobacterium* cultures were grown overnight, washed and then resuspended in a solution of 100 mM MgCl_2_, 10 µM acetosyringone. Cultures were then infiltrated into the abaxial side of *Nictotiana benthamiana* leaves at an O.D 600 of 0.1 for single infiltrations and of 0.2 for co-infiltrations (0.1 for each construct). Plants were imaged 48 h after infiltration. We manually segmented cells co-expressing nYFP:TIO-C-term and cYFP:Kin-12A-tail and quantified mean YFP fluorescence, YFP punctae per area, and RFP fluorescence. We found that there was a correlation between both mean YFP fluorescence and RFP fluorescence (*R*² = 0.5259, Fig. EV8A), as well as between YFP punctae per area and RFP fluorescence (*R*² = 0.4062, Fig. EV8B). We therefore normalized mean YFP fluorescence and YFP punctae per area by mean RFP fluorescence per cell to account for differences in rBiFC expression levels (Figs. 5 and EV8).

### Microscopy and image analysis

Confocal microscopy was performed using a Zeiss 980 Inverted CLSM with Airyscan 2 using a C-Apochromat ×40/1.20 W Corr M27 objective, and a Zeiss 980 Vertical CLSM using a C-Plan-Apochromat 40x/1.3 Oil DIC UV-Vis-IR objective. Experiments monitoring phragmoplast expansion over extended time periods used imaging chambers as described before (Kirchhelle and Moore, 2017). Imaging of CFW, GFP, YFP, RFP, FM4-64 and PI was as previously described (Chow et al, 2008). Brightfield images of GUS-stained primary roots were acquired using a Zeiss Axio Imager.M2 upright microscope. Brightfield images of whole *Arabidopsis* seedlings in Figs. 3, EV3, and 5 were acquired using a Leica MZ12 microscope with Axiocam. Image processing and analyses (sectioning, reslicing, maximum intensity projections, image assembly, and quantification) were performed using Fiji (Schindelin et al, 2012).

To quantify phragmoplast expansion rates, 3D confocal stacks acquired at 5-min intervals were resliced with an output spacing of 0.6 µm from the top and then from the left. If necessary, images were straightened to align with the vertical plane between top and left reslicing. Oval sections were then drawn around the phragmoplast(s) at successive time intervals until an oval could no longer reliably be drawn around the phragmoplast (because of fusion of one or more sections with the cell periphery). From these oval sections, the area and Feret diameters were calculated. Phragmoplast expansion rates were calculated as the change in Feret diameter between the start and end of the observation period, relative to the time elapsed.

For the quantification of fluorescence intensity at cell plates in longitudinal direction, CLSM stacks or single midplane sections of primary roots co-expressing fluorescently tagged proteins of interest were collected at Nyquist resolution (voxel size 99.5 nm × 99.5 nm × 550 nm). Midplane sections of meristematic cells were imported into Fiji, and cell plates were manually traced using a freehand line. A plot profile with a width 7 pixels was generated and fluorescence intensity was measured along the profile. Average signal intensity was calculated for 1 µm wide intervals along cell plates, which were grouped by diameter into diameter into short ( < 9 µm), medium (9–13 µm) and long ( > 13 µm). Based on our morphological quantifications of phragmoplasts, these categories correspond to disk stage (short), ring stage (long) and transitioning phragmoplasts (medium). Average intensity +/−SD was plotted using the ggplot2 function in R (Wickham, 2009). Fluorescence intensity orthogonal to cell plates was quantified from the same type of midplane sections described above, plot profiles were generated from straight lines positioned orthogonal to the cell plate with a width of 30 pixels. These were positioned at the centre of the cell plate for disk stage divisions, and at one of the leading edges for ring-stage divisions. Average signal intensity was calculated for 0.25 µm intervals, and average intensity +/−SD was plotted using the ggplot2 function in R (Wickham, 2009).

To quantify YFP:RAB-A2a punctae in the vicinity of the cell plate, single midplane sections of primary roots from WT, *kin-12a/b*, and *kin-12a/b pGL2»12 f* plants expressing YFP:RAB-A2a were collected at Nyquist resolution (voxel size 99.5 nm × 99.5 nm × 550 nm). Midplane sections of meristematic cells were imported into Fiji, and a mask was applied to isolate an area with a lateral extension of ~3 µm orthogonal to the cell plate in either direction. To enhance punctae against background signal, a white top hat filter in disk shape with a radius of 10 from the MorphoLibJ library (Legland et al, 2016) was applied. Particles were subsequently detected using the Analyze Particles command. Box plots were generated using the ggplot2 function in R (Wickham, 2009).

For quantification of phragmoplast morphology, CLSM stacks of primary roots expressing fluorescently RFP:TUB6 were collected at Nyquist resolution (voxel size 99.5 nm × 99.5 nm × 550 nm). Midplane sections of meristematic cells were generated in Fiji, and a freehand line was traced around the phragmoplast. Minimum and maximum Feret diameters were calculated for each phragmoplast in Fiji, which correspond to phragmoplast width (minimum Feret diameter) and diameter (maximum Feret diameter). Double box plots and violin plots for disk and ring-stage phragmoplasts were generated using the ggplot2 function in R (Wickham, 2009).

For quantification of rBIFC signals, CLSM stacks of *Nicotiana benthamiana* leaves co-expressing spit-YFP and mRFP1 were imported into Fiji, and maximum intensity projections of epidermal cells were made. Cells were manually segmented from maximum intensity projections using the polygon tool in Fiji (Schindelin et al, 2012), and mean intensity of YFP and mRFP1 were measured. To count the number of punctae per cell, the area outside each cell wall was cleared in Fiji, and particles were detected using the “Analyse Particles” tool with an intensity threshold of 85, a size filter of 0.20–50.00, and a circularity of 0.00–2.00. Punctae per area were calculated based on the particle number divided by cell surface area, and both mean YFP signal and punctae per area were normalized by mRFP1 intensity to account for differences in expression level in each cell.

### Statistical data analysis and plotting

Two-way ANOVA (analysis of variance) was performed in R using the aov function from the stats package (R Core Team, 2013). Tukey’s test was performed in R using the TukeyHSD function from the stats package, Student’s *t* test were performed in R using the t.test function from the stats package. Box-, Ribbon-, and Violin-plots were generated in R using the ggplot2 function (Wickham, 2009). In box plots, the median is displayed as a line, lower and upper hinges correspond to the 25th and 75th percentiles, the lower and upper whiskers extend from the hinge to the smallest or largest value no further than 1.5 * IQR from the hinge. Data beyond the end of the whiskers were plotted individually. Violin plots show the same information as box plots, with the addition of the kernel probability density of the data at different values. Ribbon blots show the data mean +/− standard deviation (shaded areas).

### Protein structure predictions

Protein structure predictions were performed using the AlphaFold3 (AF3) server. UCSF ChimeraX was used to visualize all protein structures. The coulombic electrostatic potential of the RAB-A2a/Kin-12A/TIO  complex was calculated using the coulombic command within UCSF ChimeraX. Protein-protein interfaces within the Kin-12A/RAB-A2a complex were analysed and amino acid residues for site-directed mutagenesis were identified using the PISA program (Krissinel and Henrick, 2007).

### Molecular dynamics simulation

The all-atom molecular dynamics simulation was performed using CHARMM36m force-field (Huang et al, 2017) in the GROMACS software (Abraham et al, 2015). The AF3 model of the RAB-A2a/Kin-12A/TIO complex represented an initial protein structure. Using the CHARMM-GUI web server (Jo et al, 2008), the complex was placed in a simulation box (23  × 23 × 23 nm), solvated with 0.15 M KCl in water, and neutralised. The system was energy-minimized using the steepest descent algorithm for up to 5000 steps, followed by equilibration for 125 ps. During energy minimization and equilibration, position restraints were introduced on the protein backbone and side chains. The 300-ns production run was performed with a 2 fs time step in the NPT ensemble. Pressure was set to 1 bar and maintained with the Parrinello-Rahman barostat, with a coupling constant of 5.0 ps and a compressibility of 4.5e-4. The temperature was set to 303.15 K and maintained using the Nose-Hoover thermostat with a coupling constant of 1.0 ps. The analysis was performed using standard GROMACS tools. The movie of the molecular dynamics trajectory was prepared using the Mol* viewer (Sehnal et al, 2021). Three independent simulations were performed, and the result for the longest is presented (300 ns).

### Graphics

Graphics in the paper and synopsis were created with Biorender.com.

## Supplementary information

Table EV1

Peer Review File

Movie EV1

Movie EV2

Source data Fig. 1

Source data Fig. 2

Source data Fig. 3

Source data Fig. 4

Source data Fig. 5

Source data Fig. 6

Expanded View Figures

## Data Availability

Raw microscopy data associated with this study has been deposited in BioImage Archive as accession number S-BIAD3262. The source data of this paper are collected in the following database record: biostudies:S-SCDT-10_1038-S44318-026-00804-1.
