# Supplementary material for: A Rab/Kinesin-12/kinase module couples vesicle delivery and phragmoplast dynamics during plant cell cytokinesis
Source: EMBO J. 2026 May 15;45(13):4694–732. doi: 10.1038/s44318-026-00804-1 (PMC13323771; doi:10.1038/s44318-026-00804-1)
Supplement: Supplementary file 1 — Table EV1 [file 44318_2026_804_MOESM1_ESM.docx]

**Table EV1: Primer sequences used in the study.**

| **Primer Name** | **Sequence** | **Source** |
| --- | --- | --- |
| Kin-12A-tail EcoRI-F | TTCAGAATTCCACAGTCTTGAGAAGACATTAGAACAGGAAAG | This study |
| Kin-12A-tail XhoI-R | ACAACTCGAGTTATTATATGTTGCACCTATCATAACCCG | This study |
| Kin-12B-tail EcoRI-F | AAAGAATTCGGCACGAGAGAACAGGAAAGACTTCGTTGG | This study |
| Kin-12B-tail XhoI-R | ACAACTCGAGTTATTATATGTTGCATCGGTCATACCC | This study |
| Kin-12F-tail EcoRI-F | TTCAGAATTCCACAAAGAAATTGAGGATCTGAAAAAAAAATTGC | This study |
| Kin-12F-tail XhoI-F | ACAACTCGAGCTACTACATTCCTTCTTCCTCAATAGCCGA | This study |
| Kin-12F-AscI-F | AAAGGCGCGCCTGGAGCAGGAATGGCAGACAATCGCATC | This study |
| Kin-12F-AscI-R | AAAGGCGCGCCTTGCTCCCTACATACCTTCTTCCTC | This study |
| Kin-12F-tail-AscI-F | TTTGGCGCGCCCACCATGGGAGCAAGTAATGGAGA | This study |
| TIO-AscI-F | AAAGGCGCGCCTGGAGCAGGAATGGGTGTCGAGGATTATCATGTGATAG | This study |
| TIO-AscI-R | AAAGGCGCGCCTTGCTCCTTAAGACTCACCACTGACTTTGGC | This study |
| Kin12f-CRISPR-1-F | ATTGGGAATACGTAGATAGCTACG | This study |
| Kin12f-CRISPR-1-R | AAACCGTAGCTATCTACGTATTCC | This study |
| Kin12f-CRISPR-2-F | ATTGAGTATTGAAACATCTTGACG | This study |
| Kin12f-CRISPR-2-R | AAACCGTCAAGATGTTTCAATACT | This study |
| Kin12f-CRISPR-3-F | ATTGTGCTACTAAACACTGCGTGG | This study |
| Kin12f-CRISPR-3-R | AAACCCACGCAGTGTTTAGTAGCA | This study |
| Kin12f-CRISPR-4-F | ATTGCATATCAGATCATCTTTACG | This study |
| Kin12f-CRISPR-4-R | AAACCGTAAAGATGATCTGATATG | This study |
| TIO-CRISPR-1-F | ATTGGAAACAAGGAAAGACTGACA | This study |
| TIO-CRISPR-1-R | AAACTGTCAGTCTTTCCTTGTTTC | This study |
| TIO-CRISPR-2-F | ATTGAGCTATGTCCACCAACACTG | This study |
| TIO-CRISPR-2-R | AAACCAGTGTTGGTGGACATAGCT | This study |
| TIO-CRISPR-3-F | ATTGGGCCAGGTTAATCGACTGTG | This study |
| TIO-CRISPR-3-R | AAACCACAGTCGATTAACCTGGCC | This study |
| TIO-CRISPR-4-F | ATTGAGCAGACATGTCCTCAACCA | This study |
| TIO-CRISPR-4-R | AAACTGGTTGAGGACATGTCTGCT | This study |
| Kin-12A-TBD-EcoRI-F | AAAGAATTCGGCACGAGAGAGGGATTAACAGTTGCACAGAAAC | This study |
| Kin-12B-TBD- EcoRI-F | AAAGAATTCGGCACGAGAGAAGGATTGACATTTGCACAG | This study |
| Kin-12F-TBD- EcoRI-F | AAAGAATTCGGCACGAGATCAGCATCAGCTGCTGAGGAAAAG | This study |
| Kin-12F-tail XmaI-R | TTCACCCGGGTAAAGAAATTGAGGATCTGAAAAAAAAATTGC | This study |
| Kin-12A-∆TBD-R | ACAACTCGAGTTATTCGGCTTCTTTAAGCCGAAC | This study |
| Kin-12B-∆TBD-R | ACAACTCGAGTTATTCAGCTTCTTTAAATCGAACAAG | This study |
| 12a-LP2 | GCAGGATGCACTAAAGAACTGAC | Lee et al., 2007 |
| 12a-RP2 | CAACAGCCTTATTCTGTATTGCC | Lee et al., 2007 |
| 12b-LP | CTATGGGATTTTGTGGCTCTGC | Lee et al., 2007 |
| 12b-RP | TTAGAAGTTTATTGAATCAATGCAGAT | Lee et al., 2007 |
| SALK-LBa1 | TGGTTCACGTAGTGGCCATCG |  |
| 12f-LP | ATGCTTGTCATTCTGTTGGAA | This study |
| 12f-RP | GAACCCCTTCCATATGATATC | This study |
| RAB-A2a-GW-F | GGGGACAAGTTTGTACAAAAAAGCAGGCTTCACCATGGCGAGAAGACCGGACGAAGA | This study |
| RAB-A2a-GW-R | GGGGACCACTTTGTACAAGAAAGCTGGGTCTCAAGACGATGAGCAACAAGGCTTCTT | This study |
| 12A-tail-F-P3P2 | GGGGACAACTTTGTATAATAAAGTTGTAATGTATCAGAATCACCCTGAAGTG | This study |
| 12A-tail-R-P3P2 | GGGGACCACTTTGTACAAGAAAGCTGGGTTTTATATGTTGCACCTATCATAACCCGAA | This study |
| 12F-tail-F-P3P2 | GGGGACAACTTTGTATAATAAAGTTGTAATGGGAGCAAGTAATGGAGAAC | This study |
| 12F-tail-R-P3P2 | GGGGACCACTTTGTACAAGAAAGCTGGGTTCTACATTCCTTCCTCAATAGCCGA | This study |
| TIO-tail-F-P1P4 | GGGGACAAGTTTGTACAAAAAAGCAGGCTTAGTATGCATGGAAGATAGAGATTTGCT | This study |
| TIO-tail-R-P1P4 | GGGGACAACTTTGTATAGAAAAGTTGGGTGTTAAGACTCACCACTGACTTTGG | This study |
| pGL2-section1-F | TCTCGAAGACAAGGAGTTAAGTTTGCCAACACGGGTAATA | This study |
| pGL2-section1-R | TGAGGAAGACATCGTAAATGGACGCCGACGTA | This study |
| pGL2-section2-F | TCTCGAAGACAATACGTACGTATTATACGGACGG | This study |
| pGL2-section2-R | TGAGGAAGACATGAGAAAGTTTAGCCGACGTA | This study |
| pGL2-section3-F | TCTCGAAGACAATCTCTATCCGAATTCTTTTT | This study |
| pGL2-section3-R | TGAGGAAGACAACATTATTGACATACAAATCCTGTCCCT | This study |
| Cas9-nls-F | TGA GGAAGACAACGAATTGACTTTACGCTTCTTCTTTGG | This study |
| Cas9-nls-R | TCTCGAAGACAAAATGGACAAGAAGTACAGCATTGGACT | This study |
| mturquoise-F | TTGAAGACAATTCGCAGCTGTTGAATTTTGACCTTC | This study |
| mturquoise-R | TTGAAGACAAAAGCTCATGACTCTTCTTCTTGATCAGC | This study |
| rbcs-F | TCTCGAAGACAAGCTTTTGCTTAGAGCTTTCGTTCGTAT | This study |
| rbcs-R | TGAGGAAGACAAAGCGTTTTGTCAATCAATTGGCAAGTCAT | This study |
| Kin-12A-motor-F | GAAGGAGATATACATATGGCGGAGACTGCGACGGAATC | This study |
| Kin-12A-motor-R | TTGCTCATCACAGGCACTGGATTATTTCCATCATTC | This study |
| Kin-12B-motor-F | GAAGGAGATATACATATGGAAGCTACGGCGGATAATG | This study |
| Kin-12B-motor-R | TTGCTCATCACAGGCACTGGGTTGTTTCCTTTATCAT | This study |
| Kin-12F-motor-F | GAAGGAGATATACATATGTTGAAAACTCGAAATGAAG | This study |
| Kin-12F-motor-R | TTGCTCATCACAGGCACACAAGCATCGGCTTTGACTTTT | This study |
| mClover-Kin-12A-GW-F | GGGGACAAGTTTGTACAAAAAAGCAGGCTTCACCatggtgagcaagggcgaggagctg | This study |
| mClover-Kin-12A-GW-R | GGGGACCACTTTGTACAAGAAAGCTGGGTCttatatgttgcacctatcataacccgaaaaccaagaaggc | This study |
