# Supplementary material for: A Rab/Kinesin-12/kinase module couples vesicle delivery and phragmoplast dynamics during plant cell cytokinesis
Source: EMBO J. 2026 May 15;45(13):4694–732. doi: 10.1038/s44318-026-00804-1 (PMC13323771; doi:10.1038/s44318-026-00804-1)
Supplement: Supplementary file 3 — Movie EV1 [file 44318_2026_804_MOESM3_ESM.zip › Movie EV1/Movie EV1 legend.docx]

**Movie EV1: All-atom molecular dynamics simulation of Kin-12A tail, RAB-A2a and TIO C-terminus hexamer complex.** Green & purple: RAB-A2a. Orange & yellow: Kin-12A_1019-1292_. Red and blue: TIO_343-1322._ Time period = 300 nanoseconds.
