# Supplementary material for: A Rab/Kinesin-12/kinase module couples vesicle delivery and phragmoplast dynamics during plant cell cytokinesis
Source: EMBO J. 2026 May 15;45(13):4694–732. doi: 10.1038/s44318-026-00804-1 (PMC13323771; doi:10.1038/s44318-026-00804-1)
Supplement: Supplementary file 4 — Movie EV2 [file 44318_2026_804_MOESM4_ESM.zip › Movie EV2/Movie EV2 legend.docx]

**Movie EV2: time-lapse series of multinucleate primary root epidermal cell of RFP: TUB6 in *pGL2>>tio.*** Yellow arrows = position of phragmoplast initials midzone. 5 minute intervals.
