# Supplementary material for: A Rab/Kinesin-12/kinase module couples vesicle delivery and phragmoplast dynamics during plant cell cytokinesis
Source: EMBO J. 2026 May 15;45(13):4694–732. doi: 10.1038/s44318-026-00804-1 (PMC13323771; doi:10.1038/s44318-026-00804-1)
Supplement: Supplementary file 5 — Source data Fig. 1 [file 44318_2026_804_MOESM5_ESM.zip › Fig 1/Fig 1G/Raw blot photos.pptx]

## Slide 1
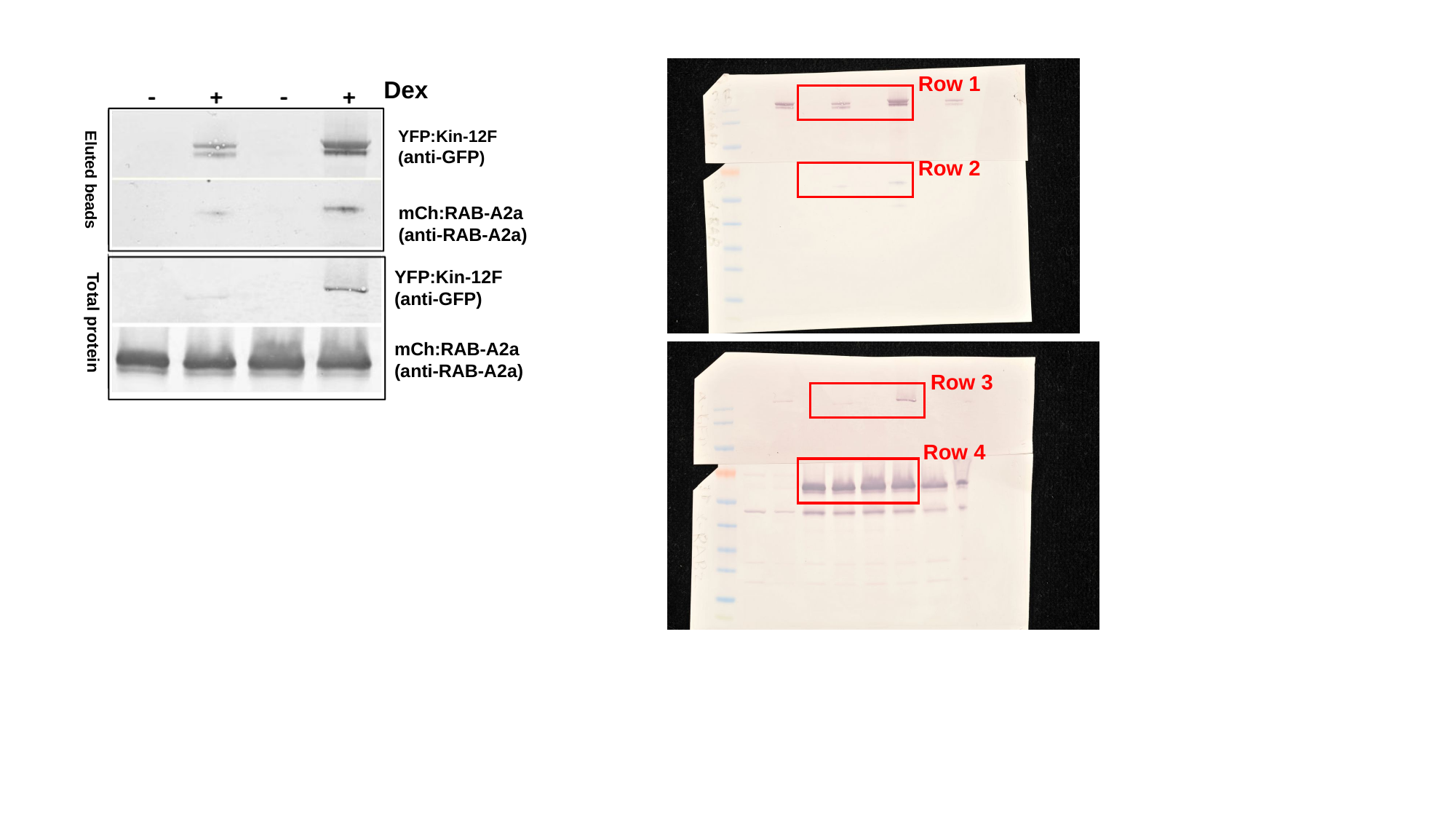

Row 1
Dex
YFP:Kin-12F
(anti-GFP)
Row 2
Eluted beads
mCh:RAB-A2a
(anti-RAB-A2a)
YFP:Kin-12F
(anti-GFP)
Total protein
mCh:RAB-A2a
(anti-RAB-A2a)
Row 3
Row 4
