# Supplementary material for: A Rab/Kinesin-12/kinase module couples vesicle delivery and phragmoplast dynamics during plant cell cytokinesis
Source: EMBO J. 2026 May 15;45(13):4694–732. doi: 10.1038/s44318-026-00804-1 (PMC13323771; doi:10.1038/s44318-026-00804-1)
Supplement: Supplementary file 5 — Source data Fig. 1 [file 44318_2026_804_MOESM5_ESM.zip › Fig 1/Fig 1B/Y2H raw photos.pptx]

## Slide 1
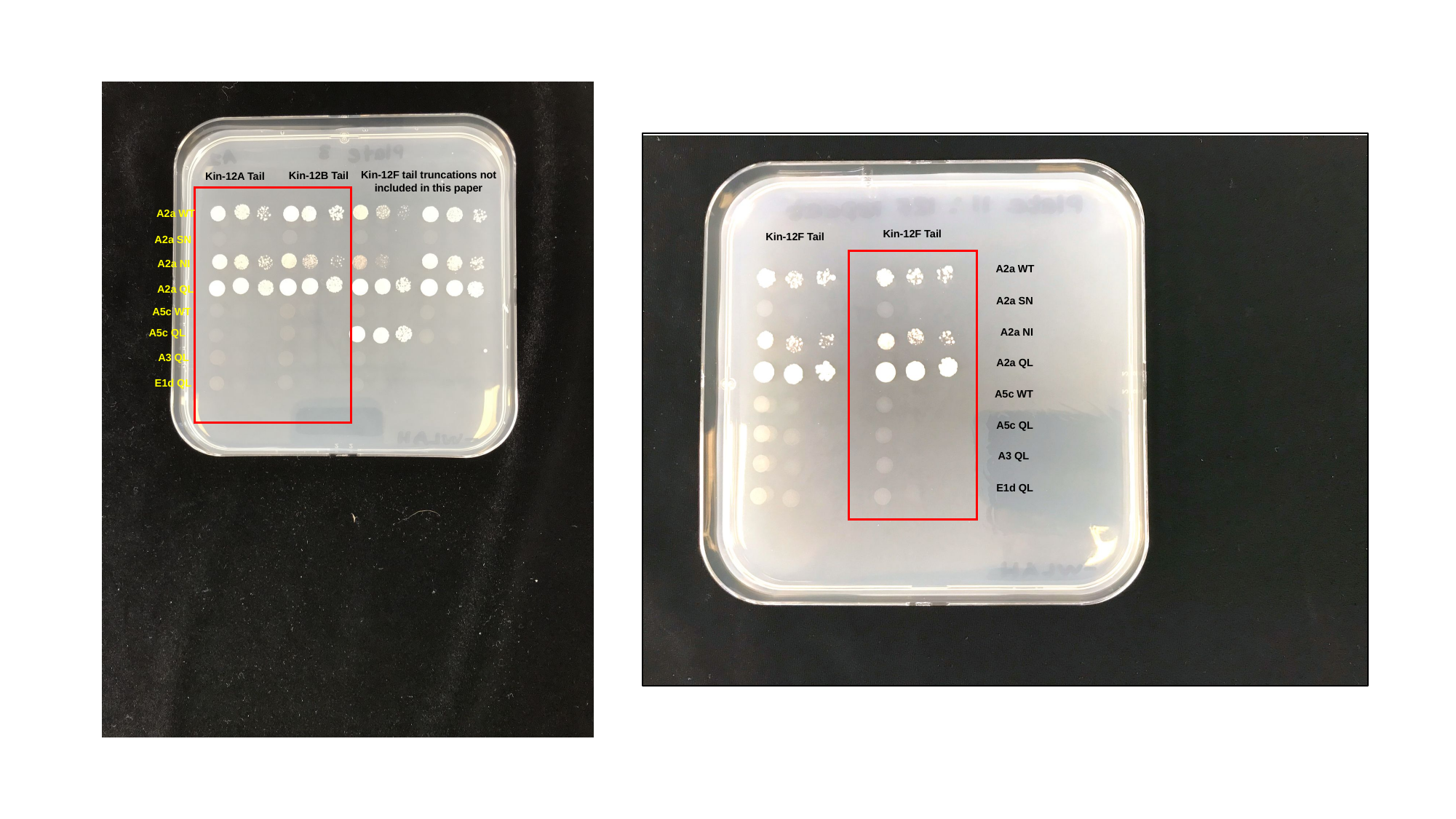

Kin-12F tail truncations not included in this paper
Kin-12B Tail
Kin-12A Tail
A2a WT
Kin-12F Tail
Kin-12F Tail
A2a SN
A2a NI
A2a WT
A2a QL
A2a SN
A5c WT
A2a NI
A5c QL
A3 QL
A2a QL
E1d QL
A5c WT
A5c QL
A3 QL
E1d QL
