# Supplementary figures and images for: A Rab/Kinesin-12/kinase module couples vesicle delivery and phragmoplast dynamics during plant cell cytokinesis
Source: EMBO J. 2026 May 15;45(13):4694–732. doi: 10.1038/s44318-026-00804-1 (PMC13323771; doi:10.1038/s44318-026-00804-1)

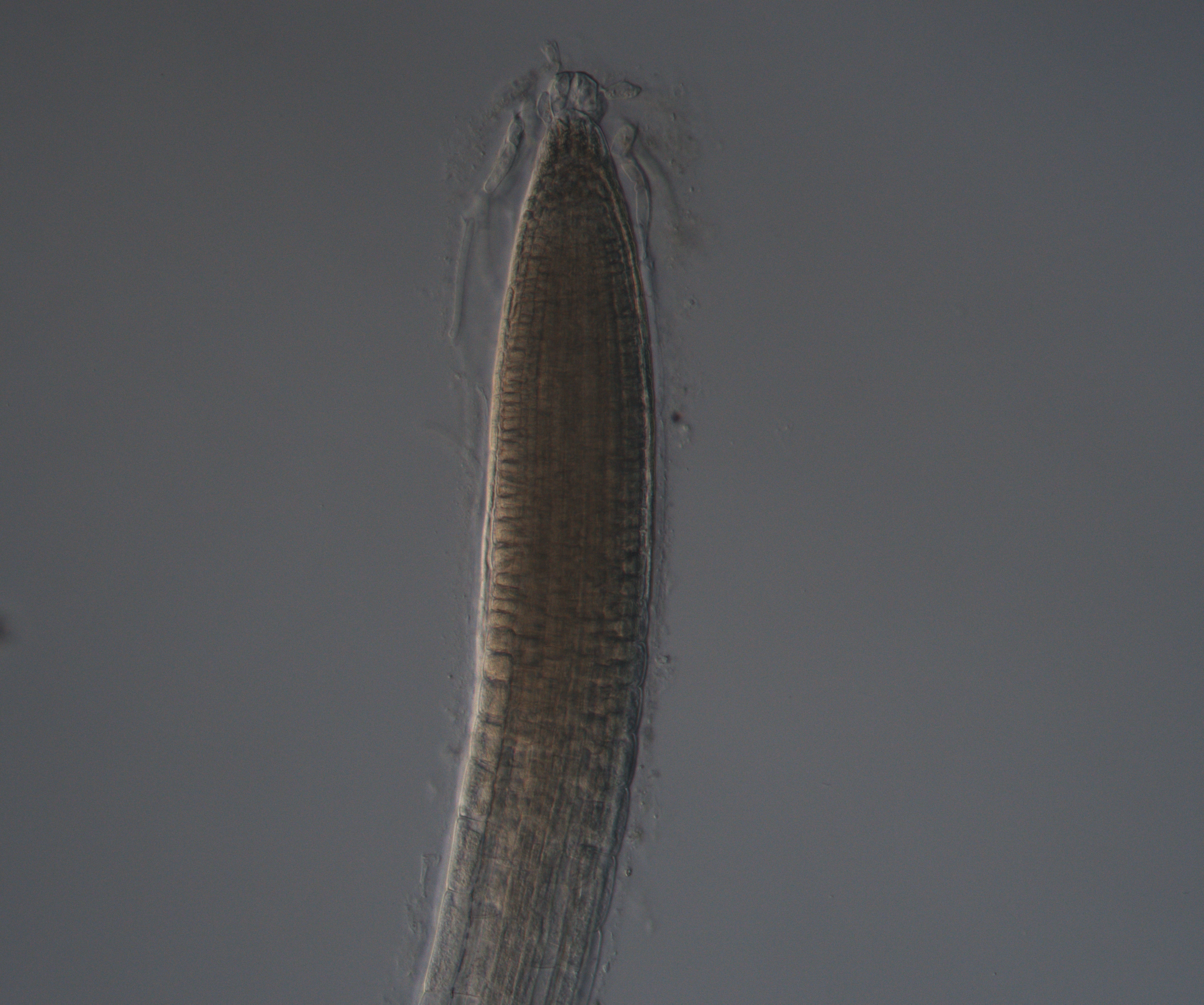

Supplement: Supplementary file 5 — Source data Fig. 1 [file 44318_2026_804_MOESM5_ESM.zip › Fig 1/Fig 1C/RAW PHOTO 3 Y12F x TUB6-R sd3 16hrs DMSO.tiff]
