# Supplementary material for: A Rab/Kinesin-12/kinase module couples vesicle delivery and phragmoplast dynamics during plant cell cytokinesis
Source: EMBO J. 2026 May 15;45(13):4694–732. doi: 10.1038/s44318-026-00804-1 (PMC13323771; doi:10.1038/s44318-026-00804-1)
Supplement: Supplementary file 8 — Source data Fig. 4 [file 44318_2026_804_MOESM8_ESM.zip › Fig 4/Fig 4H/Y2H plate photos.pptx]

## Slide 1
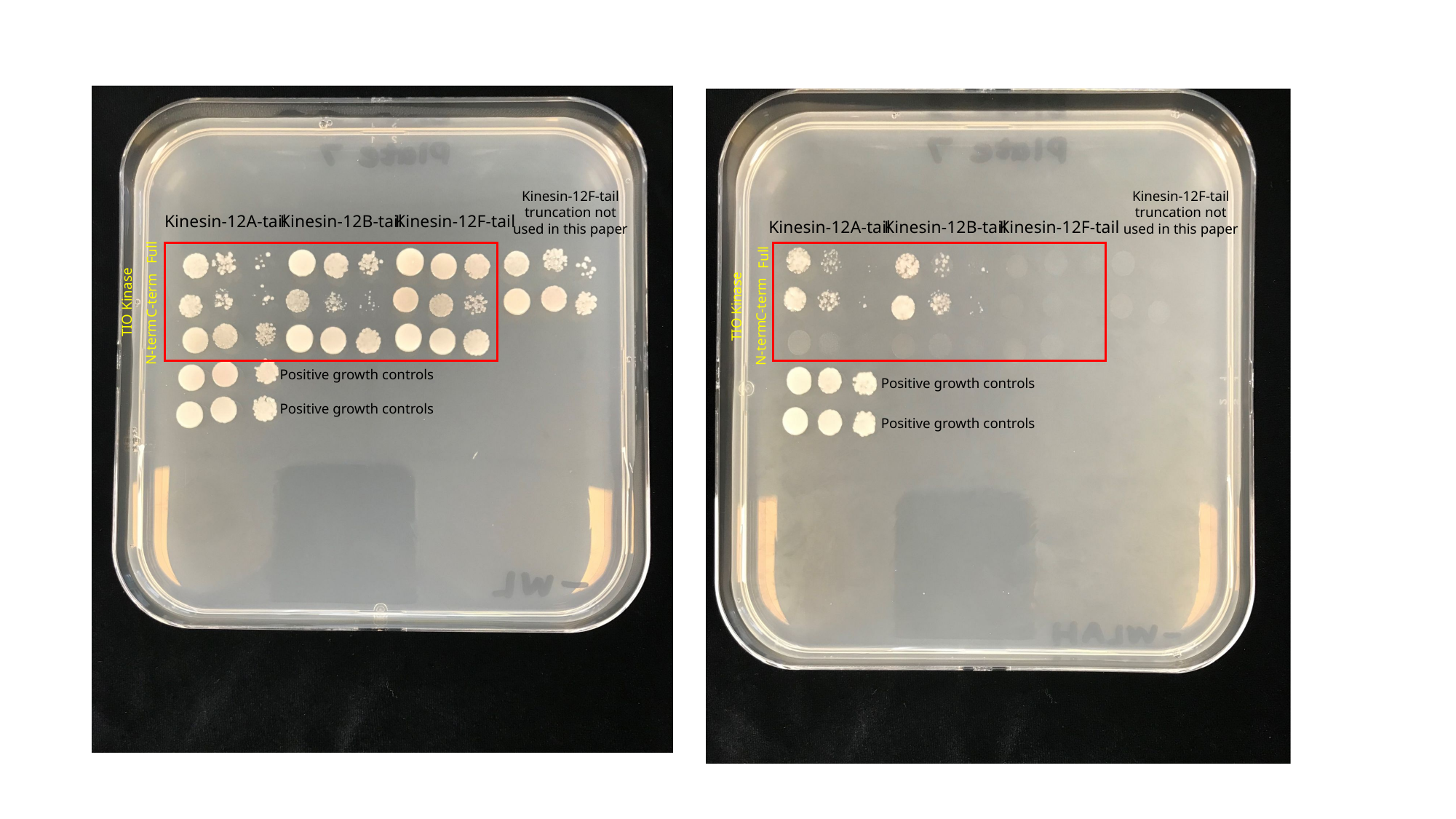

Kinesin-12F-tail truncation not used in this paper
Kinesin-12F-tail truncation not used in this paper
Kinesin-12A-tail
Kinesin-12B-tail
Kinesin-12F-tail
Kinesin-12A-tail
Kinesin-12B-tail
Kinesin-12F-tail
Full
Full
C-term
C-term
TIO Kinase
TIO Kinase
N-term
N-term
Positive growth controls
Positive growth controls
Positive growth controls
Positive growth controls
