# Supplementary material for: A Rab/Kinesin-12/kinase module couples vesicle delivery and phragmoplast dynamics during plant cell cytokinesis
Source: EMBO J. 2026 May 15;45(13):4694–732. doi: 10.1038/s44318-026-00804-1 (PMC13323771; doi:10.1038/s44318-026-00804-1)
Supplement: Supplementary file 11 — Expanded View Figures [file 44318_2026_804_MOESM11_ESM.pdf]

Expanded View Figures

Figure EV1. RAB-A2a interacts with Class II kinesin-12 members in vitro and *in planta.*

(**A**) Pairwise Y2H tests between Kin-12 tail regions and Rab-A GTPase variants on SD-Leu-Trp. Related to Fig. 1B. (**B**) Brightfield images of GUS-stained DEX» YFP: Kin-12F primary roots after 16 h treatment with either 5 µm Dex or DMSO. (**C**, **D**) CLSM section of primary roots co-expressing mCh: RAB-A2a and DEX»YFP: Kin-12F (**C**) or DEX»YFP: Kin-12F-tail (**D**). (**E**) Immunoblots analysed with anti-YFP, anti-RAB-A2a and anti-GAPDH showing co-immunoprecipitation between YFP: Kin-12F-tail and mCh: RAB-A2a in lines co-expressing DEX»YFP: Kin-12F-tail and mCh: RAB-A2a, in presence (+) or absence (−) of Dex to induce expression of YFP: Kin-12F-tail. (**F**) Immunoblots analyses of *Arabidopsis* seedling protein extract with pre-immune serum, anti-kin12F, and immune-depleted serum. Arrowhead indicates band corresponding to Kin-12F of ~125 kDa. (**G**) CLSM sections of endogenous Kin-12F immunolocalization in primary root meristematic cells using anti-kin12F and anti-tubulin in wild-type plants with standard, immune-depleted and pre-immune conditions. (**H**) CLSM section of endogenous Kinesin-12F immunolocalization using anti-kin12F and anti-tubulin in a *kin-12f* SALK insertion line. (**I**) CLSM sections of endogenous Kin-12F immunolocalization in primary root meristematic cells using anti-kin12F and anti-tubulin in wild-type plants counterstained with DAPI. Scale bars 5 µm (**G**, **H**) and 10 µm (**I**).

 © The Author(s)

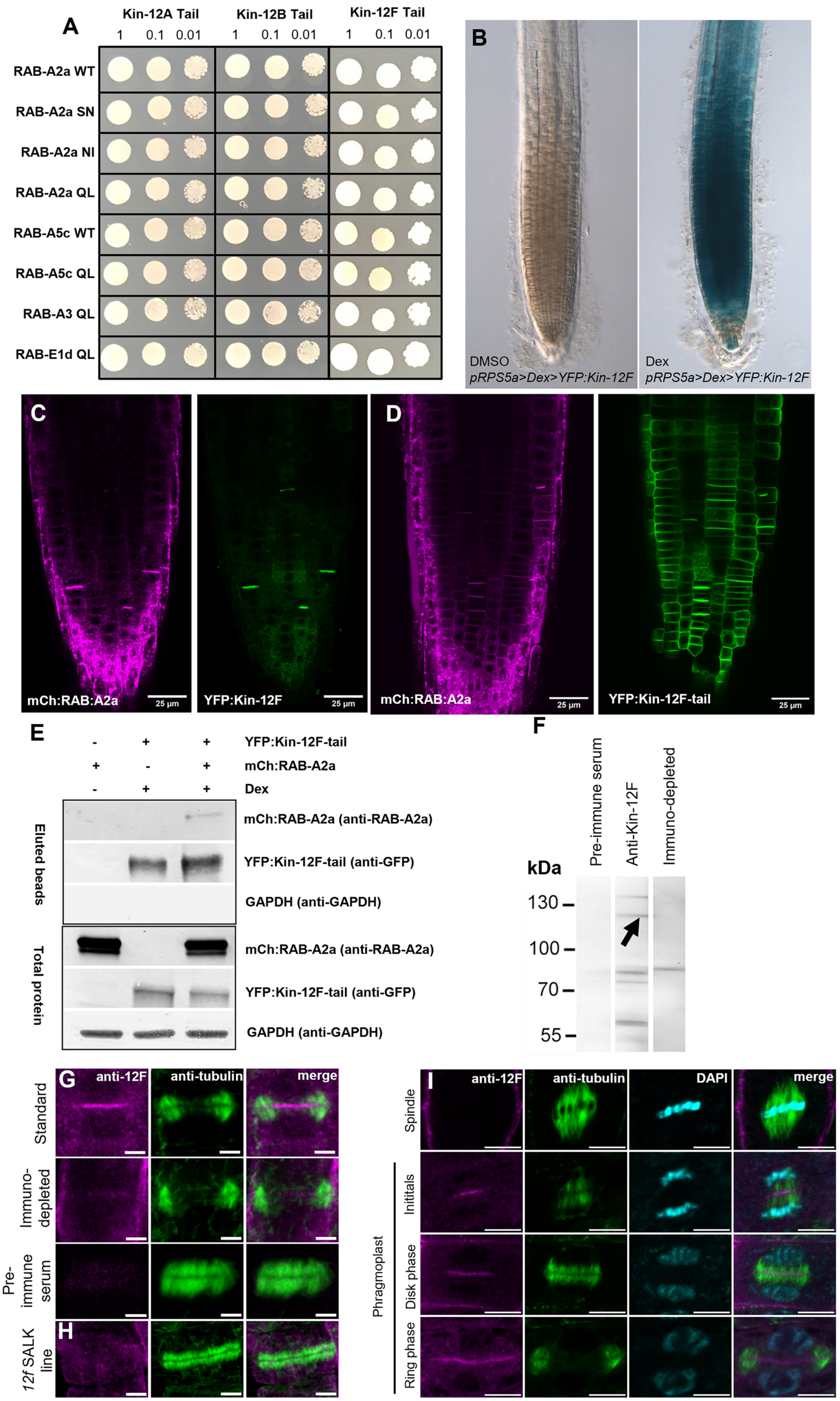
A
Kin-12A Tail
Kin-12B Tail
Kin-12F Tail
1 0.1 0.01
RAB-A2a WT
RAB-A2a SN
RAB-A2a NI
RAB-A2a QL
RAB-A5c WT
RAB-A5c QL
RAB-A3 QL
RAB-E1d QL
B
DMSO
pRPS5a>Dex>YFP:Kin-12F
Dex
pRPS5a>Dex>YFP:Kin-12F
C
mCh:RAB:A2a
YFP:Kin-12F
25 µm
D
mCh:RAB:A2a
YFP:Kin-12F-tail
25 µm
E
YFP:Kin-12F-tail
mCh:RAB-A2a
Dex
Eluted beads
Total protein
mCh:RAB-A2a (anti-RAB-A2a)
YFP:Kin-12F-tail (anti-GFP)
GAPDH (anti-GAPDH)
F
Pre-immune serum
Anti-Kin-12F
Immuno-depleted
kDa
130
100
70
55
G
anti-12F
anti-tubulin
merge
Standard
Immuno-depleted
Pre-immune serum
H
12f SALK line
I
anti-12F
anti-tubulin
DAPI
merge
Spindle
Phragmoplast
Initials
Disk phase
Ring phase

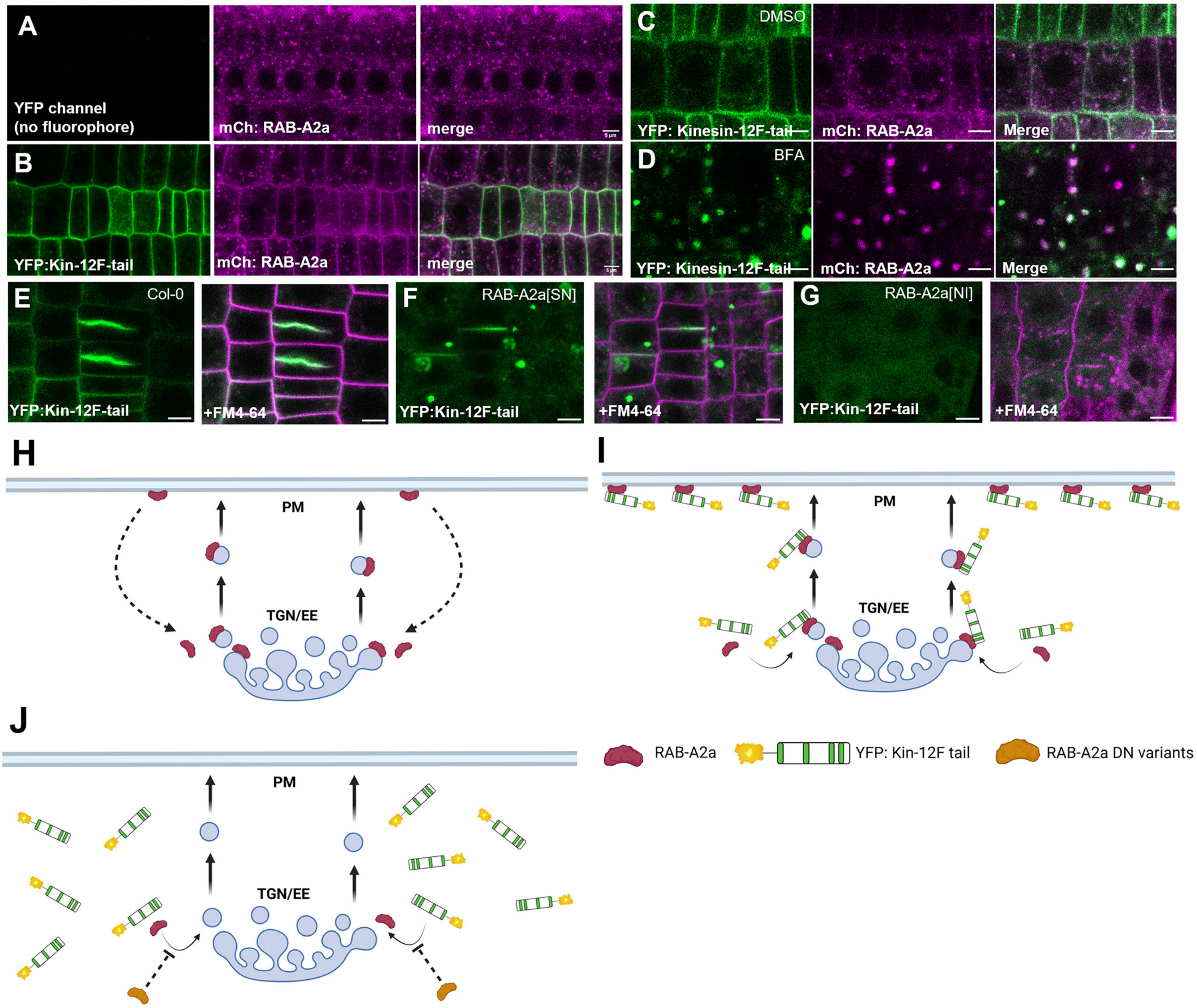

Figure EV2. The localization pattern of Kin-12F Tail affects RAB-A2a localization but is also dependent on RAB-A2a activity.

(**A**, **B**) CLSM sections of primary root epidermal meristematic cells expressing mCh: RAB-A2a without (**A**) and with (**B**) DEX»YFP: Kin-12F-tail co-expression. (**C**, **D**) CLSM sections of primary root epidermal meristematic cells co-expressing DEX»YFP:Kin-12F-tail and mCh:RAB-A2a upon 30 min treatment with 50 μM BFA (**D**) or equivalent DMSO concentration (**C**). (**E–G**) CLSM section of primary root meristematic epidermal cells expressing DEX»YFP:Kin-12F tail alone (**E**) or with DEX»RAB-A2a[SN] (**F**) or DEX»RAB-A2a[NI] (**G**) for 16 h, counterstained with FM4-64. Scale bars, 5 μm. (**H–J**) Explanatory schematics of YFP: Kin-12F tail's effect on RAB-A2a localization. During interphase, RAB-A2a is recruited to the TGN/EE from which it defines a trafficking pathway to the PM, from which it is recycled back to the TGN/EE (**H**). When expressed, YFP: Kin-12F tail associates with RAB-A2a and it transported to the PM. YFP: Kin-12F tail at the PM sequesters RAB-A2a their, either by blocking its removal from the PM or providing a competitive point of re-recruitment to the membrane (**I**). Expression of RAB-A2a dominant-negative variants interferes with membrane recruitment/ activation of RAB-A2a, blocking YFP: Kin-12F association with membranes and transport to the PM. YFP: Kin-12F therefore localises to the cytosol (**J**).

 © The Author(s)

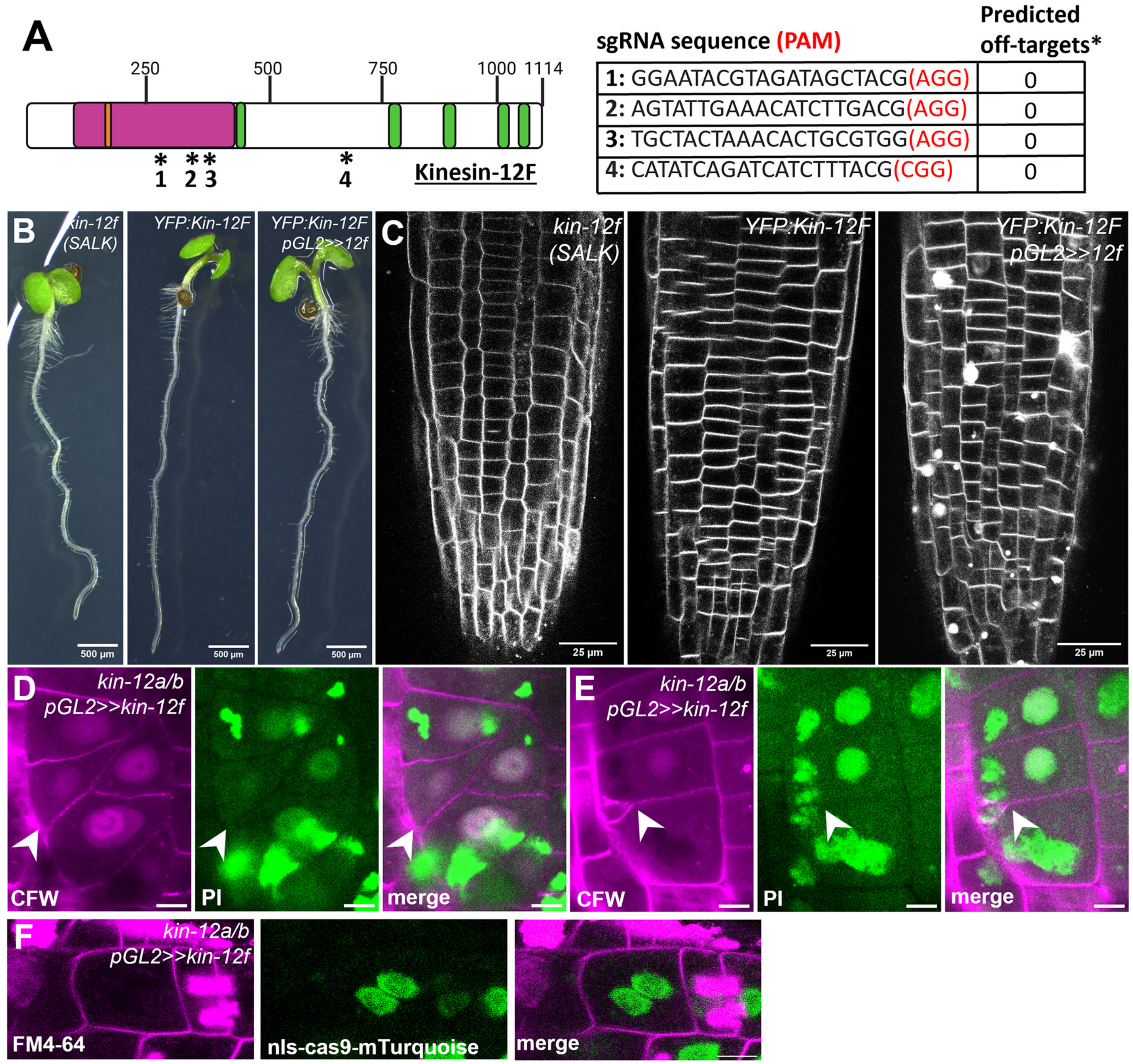

Figure EV3. Class II Kin-12 proteins are required for somatic cytokinesis and contribute to midzone membrane targeting.

(**A**) Schematic of Kinesin-12F indicating positions of sgRNAs, and table indicating sgRNA sequences with predicted off-targets. sgRNA sequences in black, adjacent PAM site in red. *Off-targets as predicted by CHOPCHOP, which searches for sequences with up to 3 mismatches in the 20 bp sequence upstream of the PAM. 0 off-targets therefore means that no sequence matches were found that have 3 or fewer mismatches with the sgRNA sequence. (**B**) Brightfield images of 5-day-old seedlings of *kin-12f SALK*, *YFP: Kin-12F*, and *YFP: Kin-12F pGL2»12 f* backgrounds. (**C**) CLSM sections of primary roots of *kin-12f SALK*, *YFP: Kin-12F*, and *YFP: Kin-12F pGL2»12 f* backgrounds counterstained with FM4-64. (**D**, **E**) CLSM section of *kin-12a/b pGL2»kin-12f* primary root meristematic epidermal cells co-stained with Calcofluor White (CFW) and Propidium Iodide (PI) showing complete but misaligned (**D**) and branched crosswalls (**E**). (**F**) CLSM section of primary roots meristematic epidermal cell in *kin-12a/b pGL2»kin-12f* background counterstained with FM4-64. Note that there is no sign of crosswall formation. Scale bars, 5 µm.

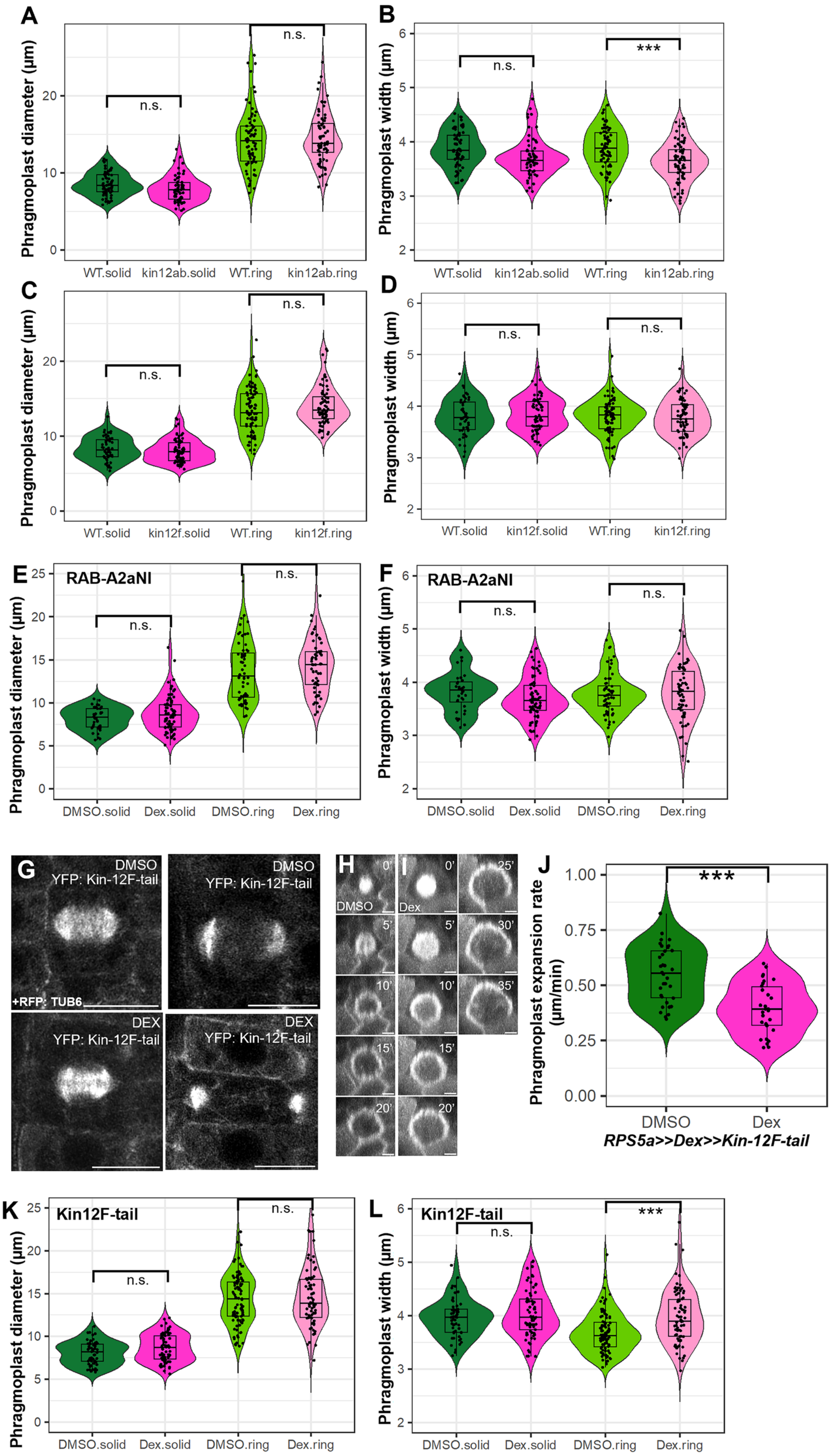
A
Phragmoplast diameter (µm)
n.s.
n.s.
WT.solid kin12ab.solid WT.ring kin12ab.ring
B
Phragmoplast width (µm)
n.s.
***
WT.solid kin12ab.solid WT.ring kin12ab.ring
C
Phragmoplast diameter (µm)
n.s.
n.s.
WT.solid kin12f.solid WT.ring kin12f.ring
D
Phragmoplast width (µm)
n.s.
n.s.
WT.solid kin12f.solid WT.ring kin12f.ring
E
RAB-A2aNI
Phragmoplast diameter (µm)
n.s.
n.s.
DMSO.solid Dex.solid DMSO.ring Dex.ring
F
RAB-A2aNI
Phragmoplast width (µm)
n.s.
n.s.
DMSO.solid Dex.solid DMSO.ring Dex.ring
G
DMSO
YFP: Kin-12F-tail
+RFP: TUB6
DEX
YFP: Kin-12F-tail
H
I
DMSO
Dex
J
Phragmoplast expansion rate (µm/min)
***
DMSO Dex
RPS5a>>Dex>>Kin-12F-tail
K
Kin12F-tail
Phragmoplast diameter (µm)
n.s.
n.s.
DMSO.solid Dex.solid DMSO.ring Dex.ring
L
Kin12F-tail
Phragmoplast width (µm)
n.s.
***
DMSO.solid Dex.solid DMSO.ring Dex.ring

 © The Author(s)

Figure EV4. Class II Kin-12 proteins and RAB-A2a are required for phragmoplast expansion.

(**A**, **B**) Violin plots of phragmoplast diameter (**A**) and width (**B**) in wild-type and *kin12a/b* backgrounds expressing *RFP:TUB6* during disk and ring phase. $N = 66$ (Col-0 disk), 67 (*kin-12a/b* disk), 73 (Col-0 ring), and 79 (*kin-12a/b* ring). There is a small but statistically significant difference in phragmoplast width between wild-type and *kin12a/b* during ring stage, otherwise phragmoplast morphology is indistinguishable (two-way ANOVA and post-hoc Tukey test, n.s. = $P \geq 0.05$; ***$P < 0.001$). (**C**, **D**) Violin plots of phragmoplast diameter (**C**) and width (**D**) in wild-type and *kin-12f* backgrounds expressing RFP:TUB6 during disk and ring phase. $N = 49$ (Col-0 disk), 68 (*kin-12f* ring), 75 (*kin-12f* disk), and 83 (Col-0 ring). Phragmoplast morphology is indistinguishable between wild-type and *kin-12f* (two-way ANOVA and post-hoc Tukey test, n.s. = $P \geq 0.05$). (**E**, **F**) Violin plots of phragmoplast diameter (**E**) and width (**F**) during disk and ring phase in presence and absence of DEX»RAB-A2aNI expression. $N = 31$ (DMSO disk), 43 (DMSO ring), 50 (Dex ring), and 57 (Dex disk). Phragmoplast morphology is indistinguishable between DMSO and Dex-treated plants (two-way ANOVA and post-hoc Tukey test, n.s. = $P \geq 0.05$). (**G**) Airyscan CLSM section of primary root meristematic epidermal cells co-expressing RFP:TUB6 and DEX»YFP: Kin-12F-tail upon 72 h treatment with 5 μM Dex or DMSO during disk phragmoplast phase (left) and ring phragmoplast phase (right). (**H**, **I**) Resliced CSLM maximum intensity projections of primary root meristematic epidermal cells co-expressing RFP:TUB6 and DEX»YFP:Kin-12F-tail upon 72 h treatment with DMSO (**H**) or 5 μM Dex (**I**). Images were taken at 5 min intervals. (**J**) Violin plots of phragmoplast expansion rates in primary root meristematic epidermal and cortex cells co-expressing RFP:TUB6 and DEX»YFP:Kin-12F-tail upon 72 h treatment with DMSO or 5 μM Dex. Expansion rate was significantly reduced in in Dex- compared to DMSO-treated plants ($P = 3.469e\text{-}06$, Student's *T* test). $N = 34$ (DMSO), 30 (Dex). (**K**, **L**) Violin plots of phragmoplast diameter (**K**) and width (**L**) during disk and ring phase in the presence and absence of DEX»YFP: Kin-12F-tail expression. $N = 57$ (DMSO disk), 73 (Dex disk), 78 (Dex ring), and 97 (DMSO ring). Phragmoplast morphology is indistinguishable between DMSO and Dex-treated plants apart from phragmoplast width at ring stage, which is increased in the presence of Dex (two-way ANOVA and post-hoc Tukey test, n.s. = $P \geq 0.05$; ***$P < 0.001$).

© The Author(s)

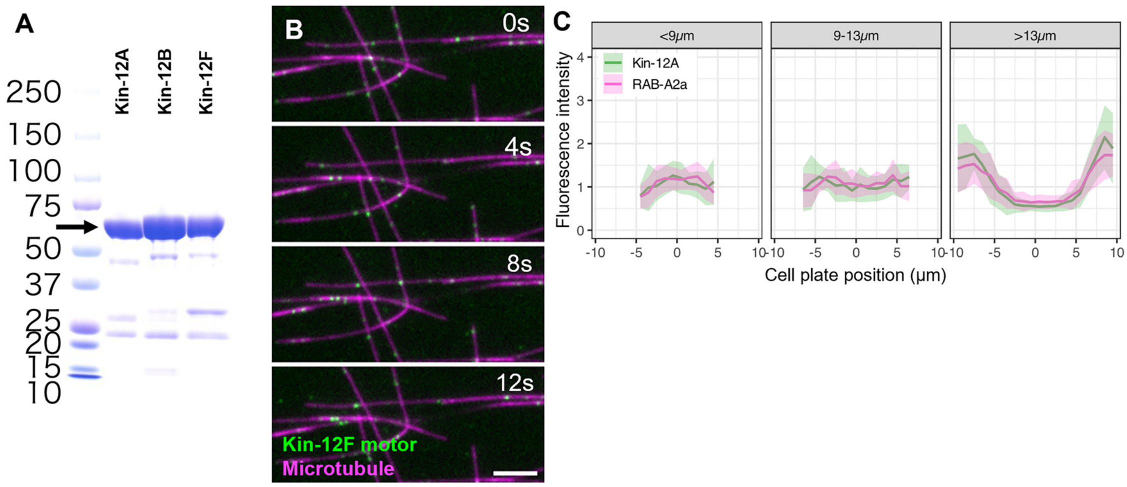

Figure EV5. Class II Kin-12 proteins have different in vitro and *in planta* properties.

(**A**) Coomassie-stained gel of Kin-12A, Kin-12B, and Kin-12F motor domains used in gliding assays as shown in (Fig 3J-L). Arrow indicates bands corresponding to kinesin motor domains. (**B**) Kymograph of Kin-12F-motor:GFP association with microtubules. Scale bars, 10 μm. (**C**) Fluorescence intensity of mCl: Kin-12A and mCh: RAB-A2a along cell plates. Cell plates were grouped by diameter into short (< 9 μm), medium (9–13 μm) and long (> 13 μm). Lines are mean values, shaded areas are +/- 1 SD. N = 11 (mCl:Kin-12A short, mCl: Kin-12A medium, mCl: Kin-12A long), 14 (YFP:Kin-12F medium).

 © The Author(s)

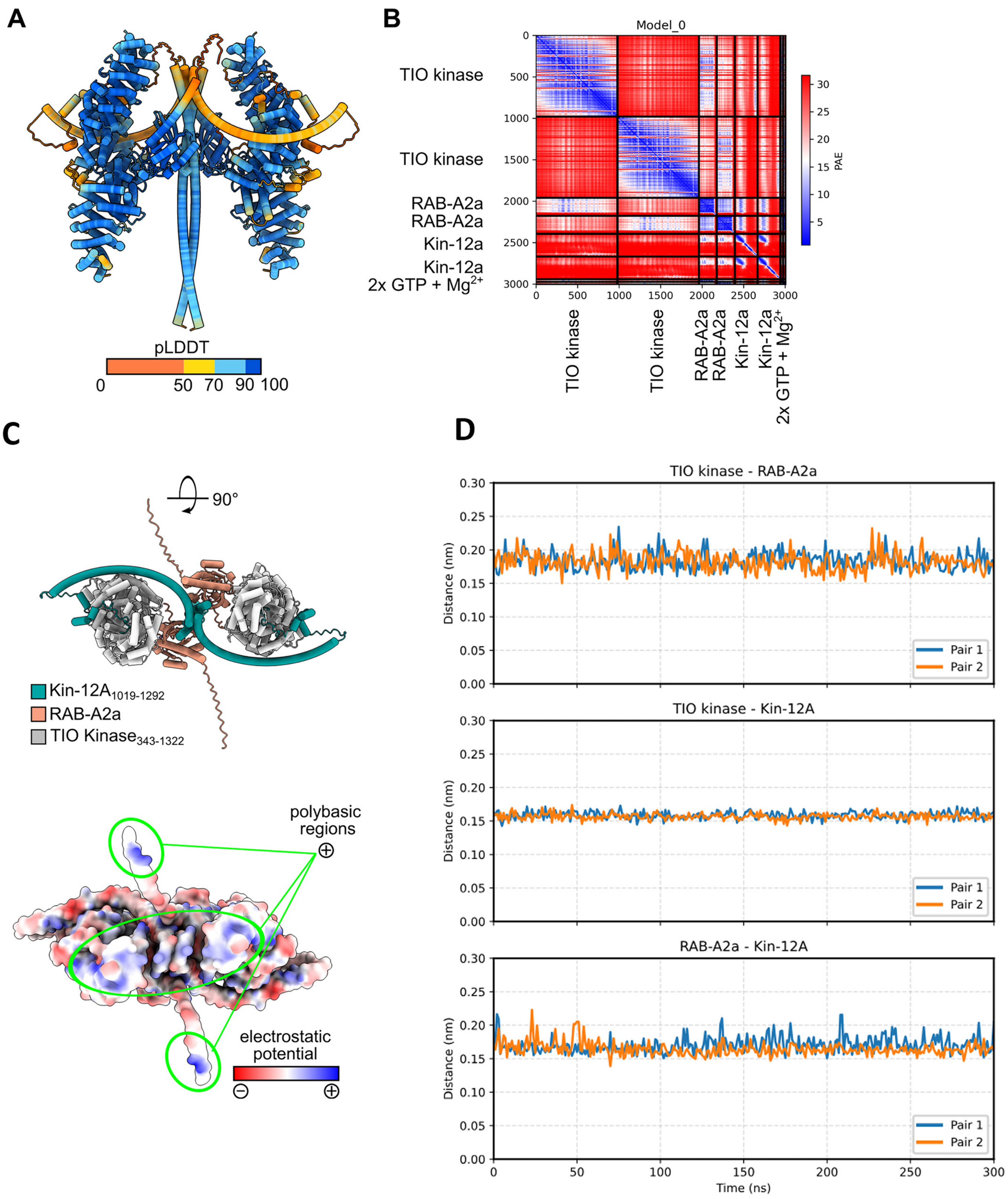
A
pLDDT
0
50
70
90
100
B
Model_0
TIO kinase
TIO kinase
RAB-A2a
RAB-A2a
Kin-12a
Kin-12a
2x GTP + Mg2+
PAE
C
90°
Kin-12A1019-1292
RAB-A2a
TIO Kinase343-1322
polybasic regions
electrostatic potential
D
TIO kinase - RAB-A2a
TIO kinase - Kin-12A
RAB-A2a - Kin-12A
Distance (nm)
Time (ns)
Pair 1
Pair 2

Figure EV6. AF3 prediction parameters and molecular dynamics prediction for the RAB-A2a/Kin-12A/TIO module.

(**A**) Confidence of AF3 local structure predictions as measured by predicted Local Distance Difference Test (pLDDT). Blue indicates highest possible confidence. (**B**) Predicted Alignment Error (PAE, Angstroms) measure of the confidence of relative positions of different residues within the AF3 predicted structure. (**C**) AF3 renderings showing base of RAB-A2a/Kin-12A/TIO kinase module with amino acid electrostatic potentials indicated. Coulombic electrostatic potential representation of the RAB-A2a/Kin-12A/TIO module. The base of the module features a high proportion of polybasic residues (positively charged), which align positionally with the negatively-charged anionic lipid composition of the cell plate. (**D**) Minimal distance (nm) between the interacting subunits of the RAB-A2a/Kin-12A/TIO kinase complex in all-atom molecular dynamics simulation over 300 nanoseconds. Pair 1 and Pair 2 refer to the two individual pairs in the hexamer complex (e.g. two pairs each for RAB-A2a/Kin-12A etc.). As for the AF3 work in Fig. 5A, the simulation incorporates the C-terminal regions of Kin-12A and TIO kinase, and the full sequence of RAB-A2a, in an overall hexamer complex. Movie version of simulation attached as Movie EV1.

 © The Author(s)

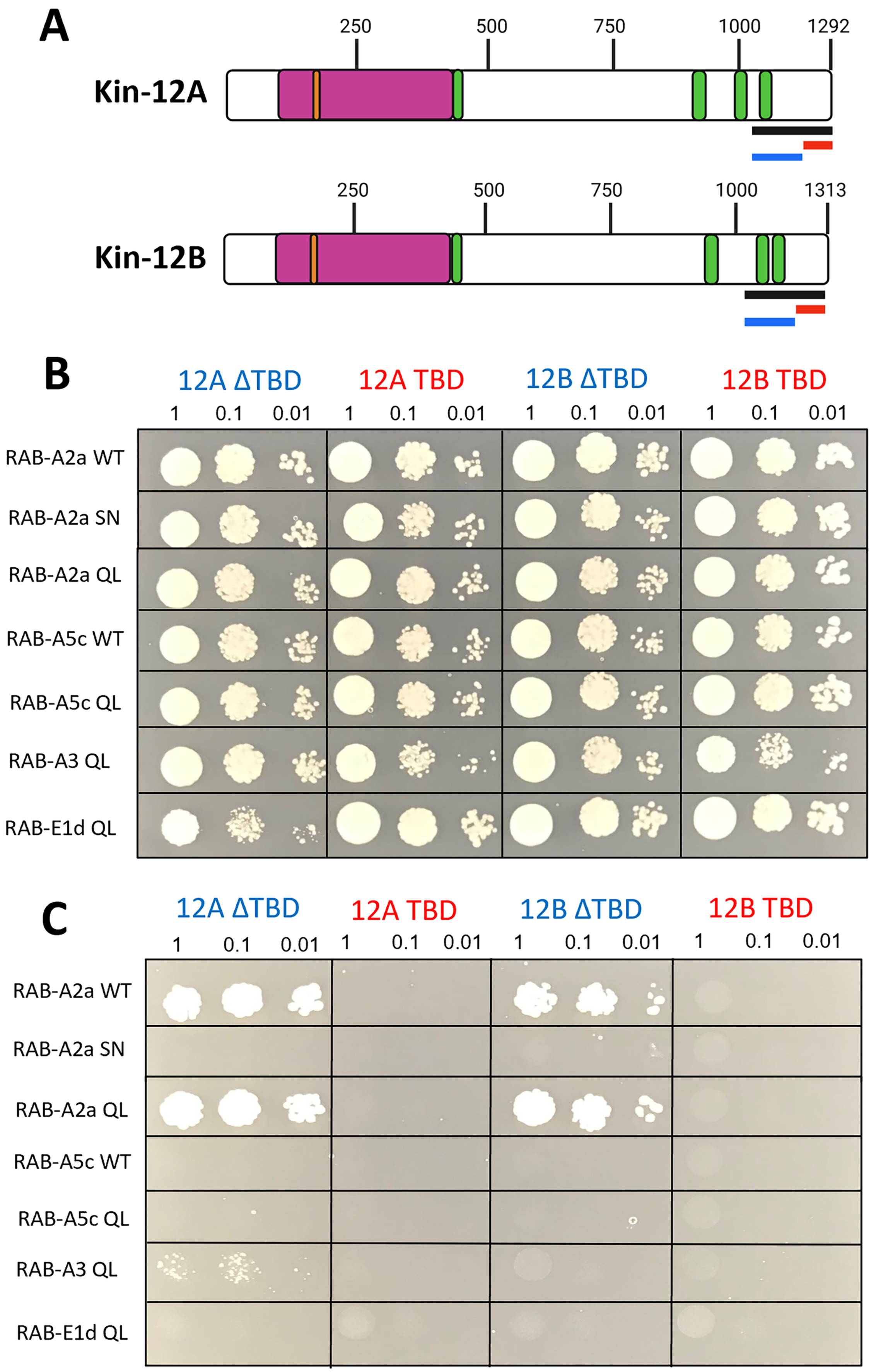
A
Kin-12A
250
500
750
1000
1292
Kin-12B
250
500
750
1000
1313
B
12A ΔTBD
12A TBD
12B ΔTBD
12B TBD
1 0.1 0.01
RAB-A2a WT
RAB-A2a SN
RAB-A2a QL
RAB-A5c WT
RAB-A5c QL
RAB-A3 QL
RAB-E1d QL
C
12A ΔTBD
12A TBD
12B ΔTBD
12B TBD
1 0.1 0.01
RAB-A2a WT
RAB-A2a SN
RAB-A2a QL
RAB-A5c WT
RAB-A5c QL
RAB-A3 QL
RAB-E1d QL

Figure EV7. The binding domains of RAB-A2a and TIO on Kin-12A/-12B are adjacent but do not overlap.

(**A**) Schematic depiction of Class II Kin-12s structure and binding domains of RAB-A2a and TIO. Black lines: clone region isolated as interactors of RAB-A2a in initial Y2H screen. Red lines: known TIO binding domains on Kin-12A/B. Blue lines: refined interaction domains of RAB-A2a (12 A/B ΔTBD) based on Y2H. (**B**, **C**) Pairwise Y2H tests between Kin-12A&B tail region truncations and Rab-A GTPase variants on SD-Leu-Trp (**B**) and SD-Leu-Trp-Ade-His (**C**).

 © The Author(s)

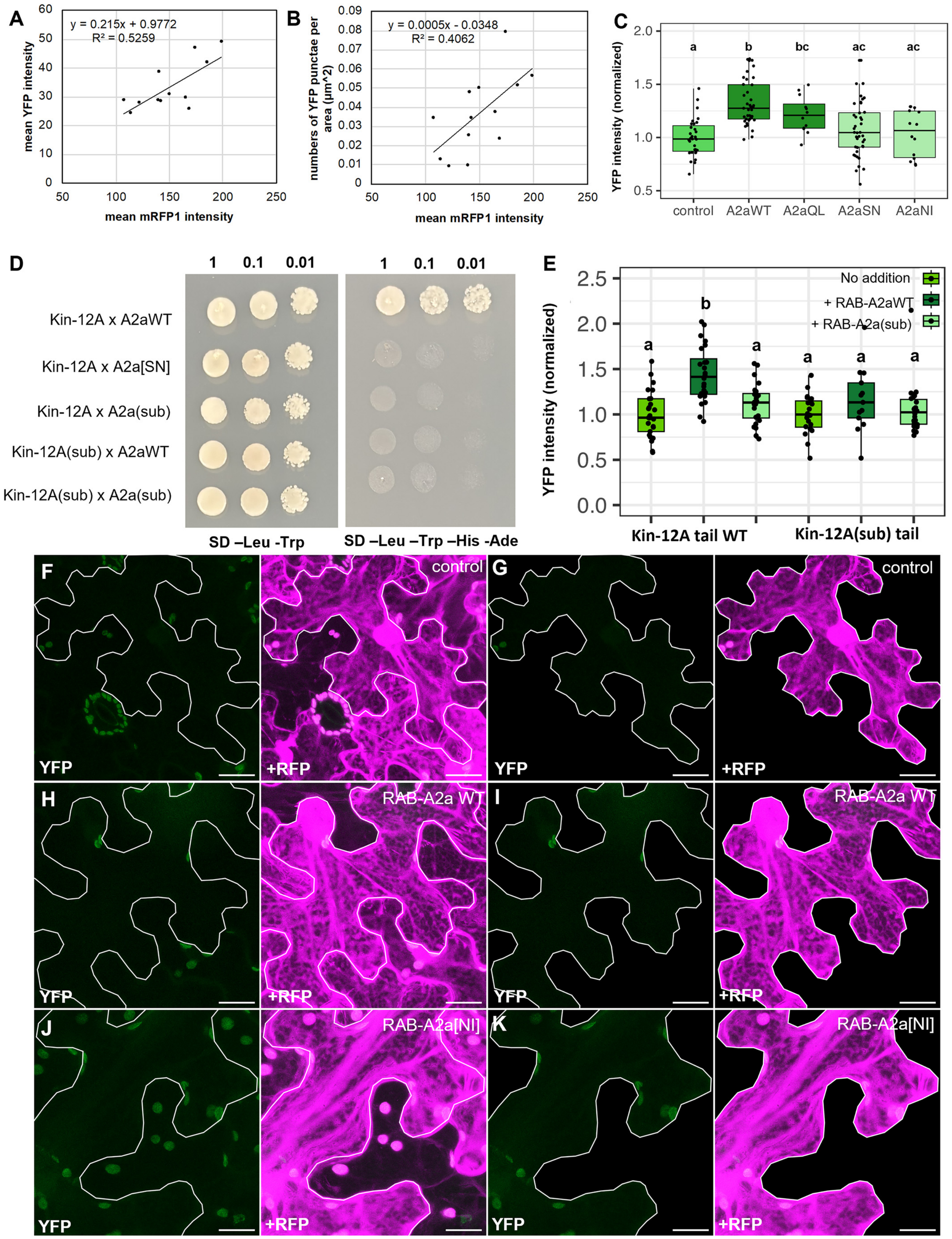
A
y = 0.215x + 0.9772
R² = 0.5259
mean YFP intensity
mean mRFP1 intensity
B
y = 0.0005x - 0.0348
R² = 0.4062
numbers of YFP punctae per area (µm^2)
mean mRFP1 intensity
C
YFP intensity (normalized)
a b bc ac ac
control A2aWT A2aQL A2aSN A2aNI
D
1 0.1 0.01
Kin-12A x A2aWT
Kin-12A x A2a[SN]
Kin-12A x A2a(sub)
Kin-12A(sub) x A2aWT
Kin-12A(sub) x A2a(sub)
SD –Leu -Trp
SD –Leu –Trp –His -Ade
E
YFP intensity (normalized)
No addition
+ RAB-A2aWT
+ RAB-A2a(sub)
Kin-12A tail WT
Kin-12A(sub) tail
F
G
H
I
J
K
control
RAB-A2a WT
RAB-A2a[NI]
YFP
+RFP

Figure EV8. Additional data relating to rBiFC assays.

(**A**, **B**) Correlation of mean YFP intensity (**A**) or mean YFP punctae per area (**B**) with mean mRFP1 intensity from *N. benthamiana* epidermal leaf cells transiently expressing nYFP:TIO and cYFP:Kin-12A and mRFP1 from a ratiometric BiFC system. (**C**) Quantification of YFP intensity normalized against mean RFP fluorescence from epidermal leaf cells transiently expressing nYFP:TIO and cYFP:Kin-12A and mRFP1 from a ratiometric BiFC system (control) or alongside RAB-A2a variants. Same letters denote no significant differences ($P \geq 0.05$) and different letters denote significant difference ($P < 0.05$), ANOVA and post-hoc Tukey test. Note the presence of RAB-A2a[WT] and RAB-A2a[QL], but not RAB-A2a[NI] or RAB-A2a[SN] significantly increased number of YFP punctae. $N = 31$ cells (control), 39 cells (A2aWT), 12 cells (A2aQL), 40 cells (A2aSN), 14 cells (A2aNI). (**D**) Pairwise Y2H tests between WT and substituted variants of Kin-12A tail region and RAB-A2a on SD-Leu-Trp and SD-Leu-Trp-Ade-His. (**E**) Quantification of mean YFP intensity per area normalized against mean RFP fluorescence in cells transiently expressing *nYFP:TIO C-terminus* with *cYFP: Kin-12A tail/cYFP: Kin-12A(sub) tail* and *mRFP1* from a ratiometric rBiFC vector alone at O.D. 600 0.1, or alongside RAB-A2aWT/RAB-A2a(sub) at O.D. 600 0.1 (combined O.D. 600 of 0.2). $N = 25$ cells (12A WT alone), 24 cells (12A WT + A2aWT), 24 cells (12A WT + A2a sub), 20 cells (12A sub alone), 14 cells (12A sub + A2a WT), 22 cells (12A sub + A2a sub) over 3 independent experiments. Same letters denote no significant differences ($P \geq 0.05$) and different letters denote significant difference ($P < 0.05$), ANOVA and post-hoc Tukey test. (**F–K**) CLSM maximum intensity projections of *N. benthamiana* epidermal leaf cells transiently expressing nYFP:TIO and cYFP:Kin-12F and mRFP1 from a ratiometric BiFC system on its own (**F**, **G**) or alongside RAB-A2aWT (**H**, **I**) or RAB-A2aNI (**J**, **K**) before (**F**, **H**, **J**) or after (**G**, **I**, **K**) cell segmentation. Note no YFP signal was detected, but chloroplast autofluorescence is visible. Scale bars, 20 µm.

 © The Author(s)

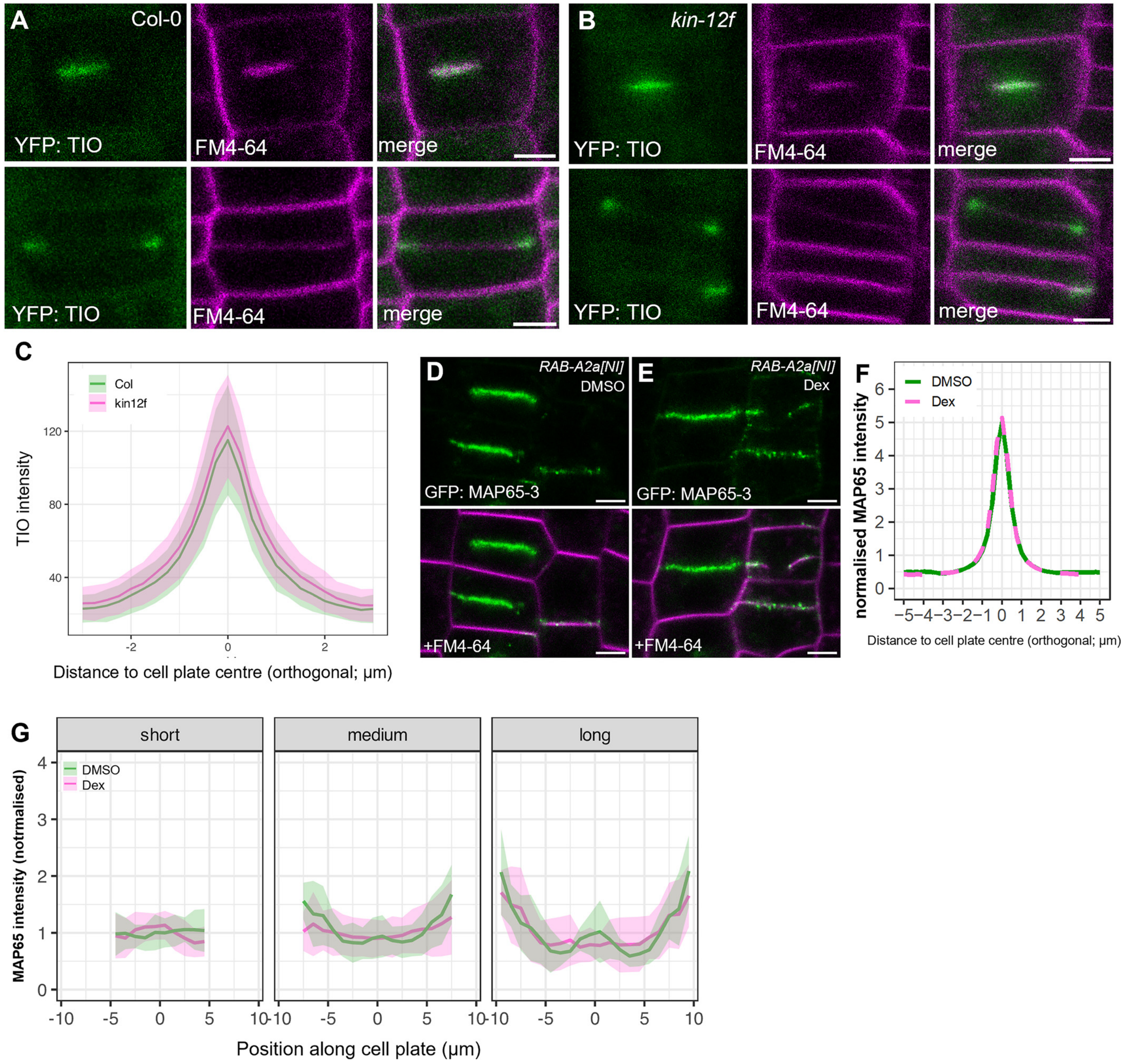

Figure EV9. Kin-12F does not affect TIO kinase intensity at the midzone and MAP65-3 localization at the midzone is not affected by expression of RAB-A2a[NI].

(**A**, **B**) CLSM sections of primary root meristematic epidermal cells expressing Dex»YFP: TIO in wild-type (**A**) and *kin-12f* (**B**). Roots were co-stained with FM4-64. Scale bars, 5 μm. (**C**) YFP: TIO enrichment orthogonal to the cell plate direction in wild-type (green) or *kin-12f* background (pink). Two-way ANOVA and post-hoc Tukey test showed no significant difference ($P > 0.05$). $N = 35$ (WT control) and 45 (kin-12f). (**D**, **E**) CLSM sections of primary root epidermal cells expressing GFP: MAP65-3 in absence (D) or presence (E) of DEX»RAB-A2aNI expression counterstained with FM4-64. (**F**) Mean normalized intensity of GFP: MAP65-3 in axis perpendicular to cell plate in presence or absence of RAB-A2a[NI]. $N = 79$ (DMSO) and 135 (Dex). (**G**) Relative fluorescence intensity of GFP: MAP65-3 along cell plates as those shown in (**D**, **E**). Cell plates were grouped by diameter into short (< 9 μm), medium (9–13 μm) and long (> 13 μm). Lines are mean values, shaded areas are +/− 1 SD. $N = 10$ (DMSO long), 13 (Dex long), 30 (DMSO medium), 42 (DMSO short), 55 (Dex short), and 60 (Dex medium). Scale bars, 5 μm.

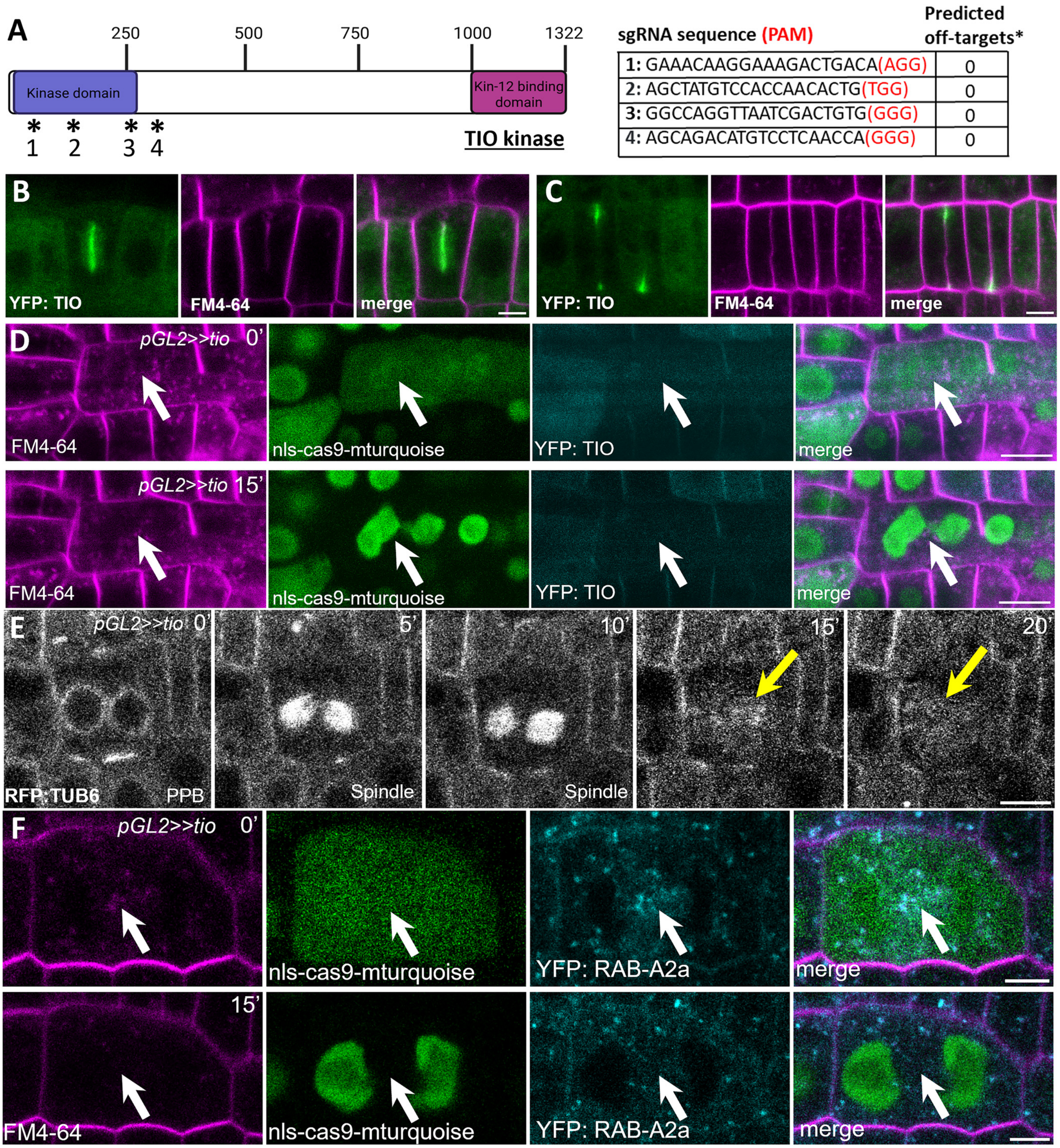
A
250
500
750
1000
1322
Kinase domain
Kin-12 binding domain
1 2 3 4
TIO kinase
sgRNA sequence (PAM)
Predicted off-targets*
1: GAAACAAGGAAAGACTGACA(AGG) 0
2: AGCTATGTCCACCAACACTG(TGG) 0
3: GGCCAGGTTAATCGACTGTG(GGG) 0
4: AGCAGACATGTCCTCAACCA(GGG) 0
B
YFP: TIO
FM4-64
merge
C
YFP: TIO
FM4-64
merge
D
pGL2>>tio 0'
FM4-64
nls-cas9-mturquoise
YFP: TIO
merge
pGL2>>tio 15'
FM4-64
nls-cas9-mturquoise
YFP: TIO
merge
E
pGL2>>tio 0'
5'
10'
15'
20'
RFP:TUB6
PPB
Spindle
Spindle
F
pGL2>>tio 0'
nls-cas9-mturquoise
YFP: RAB-A2a
merge
15'
FM4-64
nls-cas9-mturquoise
YFP: RAB-A2a
merge

 © The Author(s)

Figure EV10. TIO is required for somatic cytokinesis during which it plays a role in phragmoplast formation.

(**A**) Schematic of TIO kinase indicating positions of sgRNAs, and table indicating sgRNA sequences with predicted off-targets. sgRNA sequences in black, adjacent PAM site in red. *Off-targets as predicted by CHOPCHOP, which searches for sequences with up to 3 mismatches in the 20 bp sequence upstream of the PAM. 0 off-targets therefore means that no sequence matches were found that have 3 or fewer mismatches with the sgRNA sequence. (**B**, **C**) CLSM sections of primary root epidermal meristematic cells expressing DEX»YFP: TIO counterstained with FM4-64 during early-mid (**B**) and late (**C**) cytokinesis. (**D**) CLSM sections of primary root epidermal meristematic cells co-expressing *pGL2::tio::cas9-nls-mturquoise* (*pGL2»tio)* and DEX»YFP: TIO before (0 min) and after (15 min) nuclear reformation during cell division. White arrow indicates midzone position between future nuclei. (**E**) Sequential CLSM sections of primary root epidermal meristematic cells co-expressing *pGL2»tio* and RFP: TUB6. Yellow arrow indicates amorphous mass of microtubules at cell centre after spindle dissolution. (**F**) CLSM sections of primary root epidermal meristematic cells co-expressing *pGL2»tio* and YFP: RAB-A2a before (0 min) and after (15 min) nuclear reformation during cell division. White arrow indicates midzone position between future nuclei. Note the clustering of compartments at the midzone at 0 min but not at 15 min. Scale bar, 5 µm.
