# Supplementary material for: A Rab/Kinesin-12/kinase module couples vesicle delivery and phragmoplast dynamics during plant cell cytokinesis
Source: EMBO J. 2026 May 15;45(13):4694–732. doi: 10.1038/s44318-026-00804-1 (PMC13323771; doi:10.1038/s44318-026-00804-1)
Supplement: Supplementary file 2 — Peer Review File [file 44318_2026_804_MOESM2_ESM.pdf]

THE EMBO JOURNAL

A Rab/Kinesin-12/kinase module couples vesicle delivery and phragmoplast dynamics during plant cell cytokinesis

Liam Eliott, Moé Yamada, Monika Kalde, Michal Hála, Andrei Smertenko, Frédérique Rozier, James Evry, Niloufer Irani, Yvon Jaillais, Patrick J. Hussey, Viktor Zarsky, Roman Pleskot, Ian Moore, and Charlotte Kirchhelle

Corresponding author(s): Charlotte Kirchhelle (charlotte.kirchhelle@ens-lyon.fr) , Liam Eliott (liam.elliott@u-bordeaux.fr)

Review Timeline:

Submission Date:	25th Aug 25
Editorial Decision:	7th Oct 25
Revision Received:	25th Feb 26
Editorial Decision:	27th Mar 26
Revision Received:	20th Apr 26
Accepted:	29th Apr 26

Editor: Ieva Gailite

Transaction Report:

(Note: With the exception of the correction of typographical or spelling errors that could be a source of ambiguity, letters and reports are not edited. Depending on transfer agreements, referee reports obtained elsewhere may or may not be included in this compilation. Referee reports are anonymous unless the Referee chooses to sign their reports.)

Dear Dr. Kirchhelle,

Thank you for submitting your manuscript for consideration by the EMBO Journal. We have now received comments from three reviewers, which are included below for your information.

As you will see, the reviewers are generally positive in their assessment and appreciate the contribution of the study to the research field, while also indicating that further support would be needed for the proposed model of interactions between RABA2, kinesin-12 family proteins and TIO kinase in phragmoplast formation. Based on the generally positive assessments of reviewers, I would like to invite you to address their comments in a revised manuscript. I think that it would be useful to discuss the revision in more detail via email or phone/videoconferencing - please let me know which option you prefer.

We generally allow three months as standard revision time, which can be extended to six months in the case of major revisions. Should you foresee a problem in meeting this deadline, please let us know in advance to discuss an extension.

As a matter of policy, competing manuscripts published during this period will not negatively impact on our assessment of the conceptual advance presented by your study. However, please contact me as soon as possible upon publication of any related work to discuss the appropriate course of action.

When preparing your letter of response to the referees' comments, please bear in mind that this will form part of the Review Process File and will therefore be available online to the community. For more details on our Transparent Editorial Process, please visit our website: https://www.embopress.org/page/journal/14602075/authorguide#transparentprocess. Please also see the attached instructions for further guidelines on preparation of the revised manuscript.

Please feel free to contact me if have any further questions regarding the revision. Thank you for the opportunity to consider your work for publication, and I look forward to discussing your revision with you.

With best wishes,

Ieva Gailite

Ieva Gailite, PhD
Senior Scientific Editor
The EMBO Journal
Meyerhofstrasse 1
D-69117 Heidelberg
Tel: +4962218891309
i.gailite@embojournal.org

Instructions for preparing your revised manuscript:

Please make sure you upload a letter of response to the referees' comments together with the revised manuscript.

Please also check that the title and abstract of the manuscript are brief, yet explicit, even to non-specialists.

When assembling figures, please refer to our figure preparation guideline in order to ensure proper formatting and readability in print as well as on screen:
https://bit.ly/EMBOPressFigurePreparationGuideline
See also guidelines for figure legends: https://www.embopress.org/page/journal/14602075/authorguide#figureformat

At EMBO Press we ask authors to provide source data for the main manuscript figures. You will receive a separate email with instructions for providing source data with your revised manuscript, including how to upload and organize the files.

IMPORTANT: When you send the revision we will require
- a point-by-point response to the referees' comments, with a detailed description of the changes made (as a word file).
- a word file of the manuscript text.
- individual production quality figure files (one file per figure)
- a complete author checklist, which you can download from our author guidelines (https://www.embopress.org/page/journal/14602075/authorguide).
- Expanded View files (replacing Supplementary Information)
Please see out instructions to authors

https://www.embopress.org/page/journal/14602075/authorguide#expandedview
- a Reagents and Tools Table as part of the Methods section, which can be downloaded from our author guidelines (https://www.embopress.org/page/journal/14602075/authorguide#structuredmethods)

Please remember: Digital image enhancement is acceptable practice, as long as it accurately represents the original data and conforms to community standards. If a figure has been subjected to significant electronic manipulation, this must be noted in the figure legend or in the 'Materials and Methods' section. The editors reserve the right to request original versions of figures and the original images that were used to assemble the figure.

Further information is available in our Guide For Authors: https://www.embopress.org/page/journal/14602075/authorguide

We realize that it is difficult to revise to a specific deadline. In the interest of protecting the conceptual advance provided by the work, we recommend a revision within 3 months (5th Jan 2026). Please discuss the revision progress ahead of this time with the editor if you require more time to complete the revisions. Use the link below to submit your revision:

https://emboj.msubmit.net/cgi-bin/main.plex

Referee #1:

This work explores how Rab-A2a, 3 Kinesin 12s and TIO interact. The manuscript is beautifully written, and many of the experiments are sound and well described. The figures are nicely presented. The tools generated here, particularly the plasmids and Arabidopsis lines, are valuable and will support further discoveries. The use of tissue specific knockouts was exciting, but sometimes potentially misdirected. While this manuscript represents a lot of work, there are places where more work or clarification is needed to bolster the specific claims.

Summary: The cytokinetic GTPase Rab-A2a interacts with Kinesin12A, 12B and 12 F, and all of them localize to the phragmoplast midline, although Kin12F accumulates at the lagging edge. Interaction was demonstrated between Kin-12F and Rab-A2a by Y2H and by coIP. Conditional Cas9 tissue specific knockouts showed that a putative triple Kinesin 12A, B and F knockdown/knockout had cytokinesis defects. Rab-A2a localized to the phragmoplast midline in the mutant line, although fluorescence intensity was reduced. In vitro, movement along microtubules was observed for Kin12A and B but not Kin12F. Also, in contrast to Kin12A, Kin 12F remained at the phragmoplast midline in the map65-3 mutant. Phragmoplast expansion is slower in kin12a kin12b double mutants and kin 12f mutants but no cytokinesis defects were observed. Inducible overexpression of a less functional RabA2a also slowed phragmoplast expansion. Tio does not interact with Kin12F by Y2H but Kin12A/B do (as demonstrated earlier). Tissue specific knockout of TIO kinase was performed and cytokinetic defects were observed, similar to what has been observed in microspores, but also slowing phragmoplast expansion. Additionally, the Rab-A2a did not accumulate in tio cells [SF10]. The binding sites between TIO kinase and the kinesin 12s and the Kinesin 12 A and B with Raba2A were mapped and did not overlap. Transient interaction assays and AF3 suggested that Raba2A might enhance TIO kinase and kinesin 12 interaction.

Major comments:

The newly characterized Kinesin (12F) did not appear to participate in the TIO/Kinesin12 module, but still participated in phragmoplast expansion. How would interaction with Rab-A2a influence phragmoplast dynamics?

L166: Expression of Kin12F tail altered Rab-A2a localization, yet the conclusion was "This indicates that PM localisation of YFP:Kin-12F-tail was dependent on RAB-A2a
mediated exocytic transport from the TGN/EE25,36." ?

Was the triple mutant kin12a/b/f generated? It was surprising that tissue-specific CRISPR was used without an attempt to generate a mutant first. If it was generated, what was the phenotype? This is a critical experiment.

Relatedly, were potential off-target effects of CRISPR disruptions addressed? That YFP-Kin12F fluorescence was not detected in cells with cytokinetic defects suggests that Kin12F was disrupted but does not address whether any other genes were similarly disrupted. Analysis of multiple T2s was considered a strength, but did not alleviate concern about potential off target effects. This seems particularly relevant considering that other Kinesins may be off targets.

L 304: One image showing unidentified puncta near the cell plate was used to claim " We concluded that YFP:RAB-A2a-labelled membrane material of TGN/EE origin was partially mis-targeted in kin-12a/b pGL2>>kin-12f mutants, and that membrane mis-targeting during cytokinesis likely contributes to the partial or complete failures of cytokinesis in the absence of Class II Kin-12s, in addition to the reduced phragmoplast expansion rate." This statement would be better supported by several experiments. 1)

quantified co-localization with TNG/EE markers and 2) quantified comparison with kin12a.b mutant and Col lines. Showing that these proteins interact at the phragmoplast or phragmoplast midline would also bolster this hypothesis.

Showing kinesin motility in vitro is valuable, but it does not support the hypothesis that Rab-A2a vesicles are transported by Kinesins to the phragmoplast midline. The distinction between the localization of Rab-A2a and TIO seemed different in kin12 a/b (and f) lines.

[10.1111/j.1365-313X.2012.05077.x identified the interaction domains between Kin12A/B and TIO, and tested their ability to restore cytokinesis, which may be discussed more in the

Regarding Supplementary Figure 1: More images or some quantification of the localization with the antibody would be helpful. How often does it localize to the phragmoplast midline? How does immunodepletion alter the localization? These data are likely already available to analyze.

What are the guides used, and how specific are they for Kinesin-12F or TIO? A diagram would clarify this.

L 110: The Y2H data shown suggests that Rab-A2a binds variants with reduced nucleotide affinity, as well as unaltered Rab-A2a, and constitutively active Rab-A2a. Perhaps rephrase to "Y2H interactions suggest that either GTP-bound Rab-A2a or Rab-A2a lacking nucleotide bind Kin-12F."

Figure 5G-J: These experiments use r-BiFC in transient tobacco infiltration. As described, one cropped image each was used to demonstrate that Rab-A2a [WT] and [QL] increased Kin12A and TIO interacting puncta? Perhaps I missed something, but it was unclear from both the figure and the related SF8 and materials and methods how many times this experiment was repeated, how many cells were analyzed per independent experiment (for example: is it 31 cells or is that the number of puncta? Was the experiment repeated more than one time?). Please provide further information.

The conclusion that Rab-A2a enhances TIO and KIN12A binding seems very important for the speculative model. All of that hinging on a transient overexpression experiment in another organism in non dividing cells, where variability is frequently observed? Please discuss.

Figure 5K-O: Localization of TIO in kin-12a/b and with overexpression of Rab-A2a [SN] and [NI]. TIO localization is altered in kin-12a/b Without seeing the phragmoplast (vs. FM6-64 staining), the conclusion regarding the localization is not clear. As I understood, Rab-A2 [SN] and [NI] both inhibit cytokinesis. Whether patchy TIO localization depends on Rab-A2a or is altered in cells with cytokinetic failures seems unclear. Further, the MAP65-3 appeared patchy in SF9? L462-463.

SF10: This figure is used to show that Rab-A2a does not accumulate in tio knockdowns, but it was unclear whether there was a phragmoplast (or even a cell plate?) at the stages shown. Since the authors have the materials in hand, this could be examined more closely.

Minor comments:
Typos in L 847

Referee #2:

The GTPase RABA2a is known for its association with developing cell plate in Arabidopsis. The results summarized in this manuscript provide direct evidence showing that three homologous Kinesin-12 motors probably are its effectors. Because published results by others suggested interaction between Kin12A/B with the Fused kinase TIO, the authors reached a model of RABA2a-dependent recruitment of TIO to the phragmoplast midzone via Kin-12 motors. The work also supports a regulatory mechanism that links membrane trafficking and cytoskeletal organization during rapid expansion of the phragmoplast.

This comprehensive study answers a long-standing question regarding how RABA2a regulates cytokinesis. It also brings new insights into how cell plate-targeted membrane trafficking is tied to microtubule reorganization during rapid expansion of the phragmoplast. The authors also introduced experimental approaches that allowed conditional inactivation of essential genes in Arabidopsis. This work serves as a reference for future studies of other RAB proteins for their potential functions in the phragmoplast.

The authors are invited to provide explanations of the following points:
1. A very challenging matter related to the Arabidopsis RAB proteins is that multiple forms of RABA and perhaps RABE have been detected in the phragmoplast midzone. Although some of the published results may be questioned due to ectopic expression, it would be helpful if the authors could inform the readers whether the relationship between RABA2a and Kinesin-12 is reserved for RABA2a itself or beyond. The authors demonstrated that Kinesin-12A/B/F interacted with RABA2a but not RABA3, RABA5, and RABE by Y2H. Have other RABA2 and RABA1 been tested by similar assays? Perhaps single RABA2a mutant does not exhibit phenotypes in cell division or plant growth. Is it known whether RABA2 and other RAB genes function

redundantly in cytokinesis?
2. The authors reached a very strong conclusion for Kinesin-12 function in membrane trafficking in the phragmoplast. This conclusion was largely based on the detected interaction between the motors and RABA2a. If the working model is correct as illustrated in Figure 7, one would expect the dependence of Kinesin-12 localization on RABA2a. The question is whether the authors have such evidence.
3. It was intriguing that Kinesin-12F behaved differently from Kinesin-12A/B in the in vitro tests (Figure 4). Based on the fluorescent images presented, there was no obvious localization difference between Kin-12A/B and Kin-12F. It was unclear how the authors reached the conclusion as shown in the Figure 7 model. In the meantime, it is well known that many factors may contribute to the outcome of in vitro motility assays of kinesins. Without further evidence in vivo, perhaps it is too early to conclude Kin-12F functions as a tethering factor for older membrane compartments in the phragmoplast midzone.
4. If Kin-12F functions differently from Kin-12A/B, one would expect that the mutants behave differently although all contribute to phragmoplast expansion. It would be helpful for the authors to explain why the Kin-12F association with older membrane compartments contributes to the expansion-why step 3 is critical for steps 4 and 5 in Figure 7A.
5. It was very interesting that Kin-12A/B but not Kin-12F interact with the TIO kinase. The authors also concluded that TIO functioned upstream of phragmoplast formation. This conclusion seems contradictory to what is proposed in the model which showed the Kin-12 function in recruitment of TIO. Indeed, the tio mutant showed serious defects in phragmoplast formation (Figure 6). If the phragmoplast is not properly formed, it probably would not undergo centrifugal expansion.
6. In the manuscript, different terms were used in describing various truncated derivatives of Kin-12, including tail, TBD, delta TBD, C-term, and delta C-term. It would have been helpful if there is a diagram illustrating these different constructs, not only limited to the labeling in figure 1D. Supplementary figure 7A and B are highly repetitive so that may be combined.
7. The genome editing method described in the manuscript may be applicable to many other essential genes. Such a novelty may be emphasized if this is the first time that the method is introduced.
8. Supplementary figure 7 has a title of TIO localization but the contents are about MAP65-3 localization.
9. The Y2H results in Figure 1B and Supplementary Figure 7D seem contradictory regarding Kin-12F interactions with RABA5c and RABA3 as well as RABA2aSN. Clarification perhaps is necessary.
10. In Supplementary Figure 8, no obvious interaction between Kin-12F and TIO was detected by rBiFC when different RABA2a forms were expressed. Were similar rBiFC tests done for RABA2a and Kin-12?

Referee #3:

The motor protein family kinesin-12 (KIN12) is crucial for cell division in plants. The family is divided into two classes that overlap and differ in their localization patterns and functions. Until now, the functions of class II kinesin-12 have been associated with phragmoplast organization in *Arabidopsis*, but a recent report on kinesin-12-II members in Physcomitrium suggests involvement in vesicle transport to the cell plate. Kirchelle's research group is investigating the function of class II KIN12F and also reports a role in vesicle transport, confirming the findings from moss.

Working on members of the KIN12 family is challenging due to multiple functional redundancies and the potential lethality of knockout mutants. The authors circumvent these problems by using tissue-specific knockdown and rescue experiments. They found distinct localizations and functions for KIN12A/B and KIN12F during cytokinesis, and their simultaneous knockout resulted in additive effects. Kin12ab/f triple mutants are stunted and exhibit cell wall stubs, branched cell walls, and multinucleated cells- all characteristics of severe cytokinesis defects known from mutants impaired in phragmoplast organization or vesicle fusion. While KIN12A/B are localized in the anterior zone of the phragmoplast, KIN12F remains in the posterior zone of the phragmoplast. Consistent with its localization, KIN12F does not interact genetically with MAP65-3, as its localization remains unchanged in *map65-3* mutants. All three class II KIN12s interact with the small GTPase RabA2a, which is involved in vesicle transport. In addition, TIO kinase interacts with KIN12A/B, but not with KIN12F. Conditional knockout of TIO kinase under the GLABRA2 promoter induced cytokinesis defects comparable to those observed previously, including membrane mis-targeting.

The study provides a molecular framework involving several interaction partners in which KIN12F and its close orthologs KIN12A and KIN 12B function. Based on these results, an improved view on how phragmoplast organization and expansion are coupled with vesicle delivery and cell plate formation emerges and clarifies how feedback signaling between microtubule dynamics and cell plate formation might be mediated by a TIO kinase - KIN 12A/B -RabA2a- KIN 12F module. The authors proposed a plausible model based on their findings. RabA2a delivery depends on KIN12A/B. KIN12A/B recruits TIO kinase and RabA2a is required for its turnover at the phragmoplast. Non-motile-KIN12F provides a platform for RabA2a vesicles for cell plate formation. This report is a major advancement towards our understanding on the long-standing question of how phragmoplast organization and cell plate expansion are linked.

I find the report very thorough; it adds new findings, repeats and confirms earlier findings, and summarizes them in an attractive model.

Only the function of KIN12 is still somewhat unclear to me. The authors suggest that KIN12F forms a platform for vesicle fusion and reason that this is due to the incomplete ATP hydrolysis motif. Can KIN12F be converted into a mobile kinesin by replacing the incomplete ATP hydrolysis motif with one from KIN12A or KIN12B?

Referee #1:

This work explores how Rab-A2a, 3 Kinesin 12s and TIO interact. The manuscript is beautifully written, and many of the experiments are sound and well described. The figures are nicely presented. The tools generated here, particularly the plasmids and Arabidopsis lines, are valuable and will support further discoveries. The use of tissue specific knockouts was exciting, but sometimes potentially misdirected. While this manuscript represents a lot of work, there are places where more work or clarification is needed to bolster the specific claims.

Summary: The cytokinetic GTPase Rab-A2a interacts with Kinesin12A, 12B and 12 F, and all of them localize to the phragmoplast midline, although Kin12F accumulates at the lagging edge. Interaction was demonstrated between Kin-12F and Rab-A2a by Y2H and by coIP. Conditional Cas9 tissue specific knockouts showed that a putative triple Kinesin 12A, B and F knockdown/knockout had cytokinesis defects. Rab-A2a localized to the phragmoplast midline in the mutant line, although fluorescence intensity was reduced. In vitro, movement along microtubules was observed for Kin12A and B but not Kin12F. Also, in contrast to Kin12A, Kin 12F remained at the phragmoplast midline in the map65-3 mutant. Phragmoplast expansion is slower in kin12a kin12b double mutants and kin 12f mutants but no cytokinesis defects were observed. Inducible overexpression of a less functional RabA2a also slowed phragmoplast expansion. Tio does not interact with Kin12F by Y2H but Kin12A/B do (as demonstrated earlier). Tissue specific knockout of TIO kinase was performed and cytokinetic defects were observed, similar to what has been observed in microspores, but also slowing phragmoplast expansion. Additionally, the Rab-A2a did not accumulate in tio cells [SF10]. The binding sites between TIO kinase and the kinesin 12s and the Kinesin 12 A and B with Raba2A were mapped and did not overlap. Transient interaction assays and AF3 suggested that Raba2A might enhance TIO kinase and kinesin 12 interaction.

Major comments:

The newly characterized Kinesin (12F) did not appear to participate in the TIO/Kinesin12 module, but still participated in phragmoplast expansion. How would interaction with Rab-A2a influence phragmoplast dynamics?

We agree that it is not entirely clear why loss of KIN-12F affects phragmoplast expansion when it does not appear to bind TIO. One possibility is that stabilisation of RAB-A2a-containing vesicles at the lagging edge contributes to local enrichment of RAB-A2a at the midzone and thus enhances the potential for RAB-A2a/KIN-12A/B interactions promoting TIO recruitment. To test whether KIN-12F promotes TIO recruitment though such an indirect mechanism, we have now performed additional experiments that show YFP-TIO patterning at the midzone is not disrupted in the *kinesin-12f* SALK background that has reduced phragmoplast expansion rates (Fig S9A-C). This data suggests that rather than indirectly patterning TIO, Kin-12F plays a TIO-independent role in microtubule organization at the lagging zone that is required for proper phragmoplast expansion rates, e.g. through ensuring microtubule turnover. Alternatively, while we can find no evidence for Kin-12F binding to TIO, we cannot exclude that it is a target of TIO kinase activity in a transient “kiss and run” type interaction, or that it is a target of secondary factors downstream of TIO. Our follow-up priority is identifying the molecular targets of TIO, but further investigation here is beyond the scope of this manuscript. The phenotypes produced by loss of Kin-12A, B and F (slow expansion, sometimes failure to form phragmoplasts, amorphous membrane structures), align with those of loss of TIO kinase (failure to form phragmoplasts, slow and aberrant expansion in those that do form, amorphous membrane structures). This strongly indicates connected or at least parallel functions of these kinesins and TIO kinase, even if the finer details have yet to be resolved. We have now added new text in L671-675 to discuss this further.

L166: Expression of Kin12F tail altered Rab-A2a localization, yet the conclusion was "This indicates that PM localisation of YFP:Kin-12F-tail was dependent on RAB-A2a mediated exocytic transport from the TGN/EE25,36." ?

We have added an explanatory graphic to supplementary figure S2 to explain our reasoning, which we believe is sound. Expression of dominant-negative forms of RAB-A2a abolishes the association of Kin-12F tail with membranes, and renders it cytosolic. This indicates that RAB-A2a pulls Kin-12F onto membranes via their interaction. As RAB-A2a during interphase functions in secretory traffic between the TGN/EE and PM, we conclude that Kin-12F is pulled into secretion from the TGN/EE to the PM by RAB-A2a, and then persists in complex with RAB-A2a at the PM. This would result in the enhanced PM localization of RAB-A2a when it is co-expressed with Kin-12F tail. This is consistent with our observation that Kin-12F tail also accumulates in BFA bodies, which are largely made up of TGN/EE-derived membranes.

Was the triple mutant kin12a/b/f generated? It was surprising that tissue-specific CRISPR was used without an attempt to generate a mutant first. If it was generated, what was the phenotype? This is a critical experiment.

This we have attempted. We first tried to make a *kin-12a/b/f* mutant by crossing *kin-12a/b* and *kin-12f* SALK mutants. Despite genotyping over 600 F3 and F4 plants, we were not able to recover a *kin-12a/b/f* mutant. We then attempted stable line CRISPR (from pRPS5a) in the *kin-12a/b* background using the same sgRNAs

described here or *kin-12f*. In the T1 generation, we recovered plants that were heterozygous for a *kin-12f* mutation, which were all sterile. We also recovered a small number of plants which were genotyped as homozygous for a *kin-12f* mutation, but these died very shortly after being put onto soil. However, the *kin-12f* CRISPR in the wild-type background produced healthy T1 plants with no sterility issues. We therefore concluded that stable *kin-12a/b/f* mutants are likely lethal, and then turned to the tissue-specific CRISPR approach described here. Lethality of stable *kin-12a/b/f* mutants in *Arabidopsis* would be consistent with recent work that tried and failed to recover complete Class II Kinesin-12 knockouts in *Physcomitrium* (Yamada et al., 2025). We have included a brief summary regarding our failure to isolate stable triple knock-outs in the manuscript to clarify, although we kept it concise to not overcrowd the manuscript. We can expand further on this section if the reviewers think this would be useful.

Relatedly, were potential off-target effects of CRISPR disruptions addressed? That YFP-Kin12F fluorescence was not detected in cells with cytokinetic defects suggests that Kin12F was disrupted but does not address whether any other genes were similarly disrupted. Analysis of multiple T2s was considered a strength, but did not alleviate concern about potential off target effects. This seems particularly relevant considering that other Kinesins may be off targets.

We agree that one risk with tissue-specific CRISPR approaches is off-target effects. When designing the sgRNAs we took care to ensure that there were no predicted off-targets (see related comment from Reviewer 2 below). Moreover, the fact that we observed, similar, unusual phenotypes between our distinct CRISPR lines targeting Kin-12F and TIO kinase (slow phragmoplast expansion, phragmoplast formation failure, amorphous membrane structures) indicate that our approach has achieved good specificity. In the case of TIO kinase, we can provide further experimental evidence. During this study, we also attempted to strongly over-express TIO kinase in homozygous lines for prolonged periods, predicting that this might speed up phragmoplast expansion. However, we found that these lines have the same phenotype as *pGL2>tio* CRIPSR lines: large multinucleate cells and dramatic failures in phragmoplast formation (Response Fig 1A-E). We investigated the cause of this and, in short, we had accidentally made an inducible silencing knock-down of TIO kinase (Response Figure 1F-G). The fact that this inducible knock-down has the same phenotype as the tissue-specific CRISPR mutant strongly supports specificity in the CRISPR mutant. We do not feel that inclusion of this data in the manuscript would be productive as it would complicate an already crowded story, but summarize it here and would be happy for it to be included in a published review document. Additional information on the sgRNAs and off-target parameters has been added to Fig S3 and S10 as requested by reviewer 2.

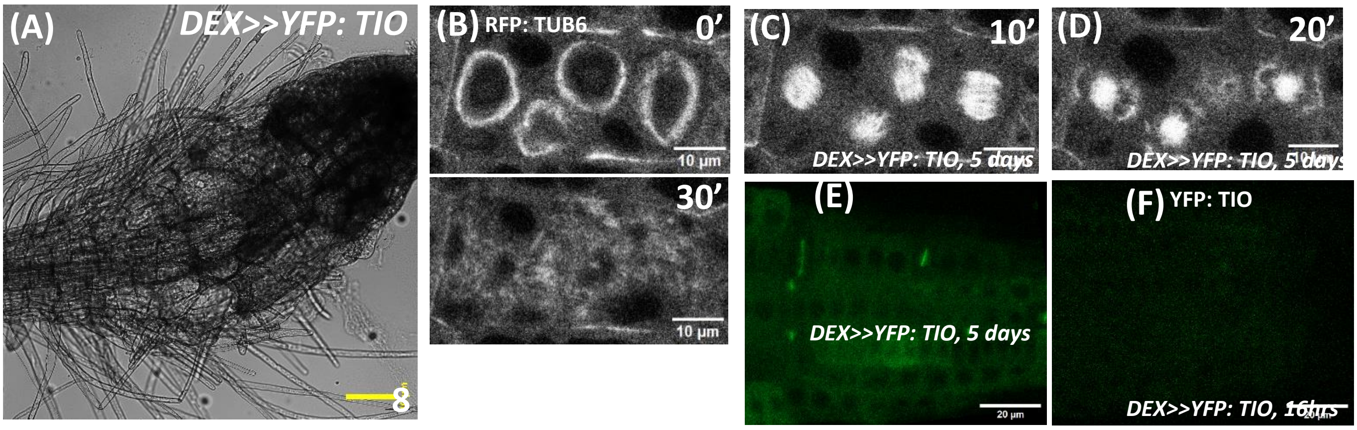

Response figure 1: Prolonged over-expression of YFP: TIO kinase inhibits phragmoplast formation due to silencing. (A) Brightfield image of homozygous *DEX>>YFP: TIO* line primary root grown on 5µM DEX for 8 days, displaying swollen cells and root tip. **(B-E)** CLSM slices of homozygous *DEX>>YFP: TIO* line co-expressing RFP: TUB6 at 10 min intervals grown on 5µM DEX for 5 days. Note the identical phenotype to the *pGL2>>tio* CRISPR line; phragmoplast initials form but fail to progress further. **(F-G)** CLSM slices of homozygous *DEX>>YFP: TIO* line at 16hrs (F) vs. 48hrs (G) induction with 5µM DEX. Prolonged homozygous *DEX>>YFP: TIO* expression leads to a sudden loss of YFP: TIO signal at around 48hrs.

L 304: One image showing unidentified puncta near the cell plate was used to claim " We concluded that YFP:RAB-A2a-labelled membrane material of TGN/EE origin was partially mis-targeted in kin-12a/b pGL2>>kin-12f mutants, and that membrane mis-targeting during cytokinesis likely contributes to the partial or complete failures of cytokinesis in the absence of Class II Kin-12s, in addition to the reduced phragmoplast expansion rate." This statement would be better supported by several experiments. 1) quantified co-localization with TNG/EE markers and 2) quantified comparison with kin12a.b mutant and Col lines. Showing that these proteins interact at the phragmoplast or phragmoplast midline would also bolster this hypothesis.

We have now developed a bespoke particle identification pipeline to quantify the YFP:RAB-A2a/FM4-64 puncta around the cell plate in the *kin-12a/b pGL2>>kin-12f* mutants compared to *kin-12a/b* and Col-0 (Fig 3H, L291-

294). This shows that significantly greater numbers of these punctae are present around the plate in *kin-12a/b pGL2>>kin-12f* mutants. We have not included an additional TGN/EE marker as this would require introducing an additional construct into an already triple mutant, which we believe is beyond the scope of reasonable revisions.

Showing kinesin motility in vitro is valuable, but it does not support the hypothesis that Rab-A2a vesicles are transported by Kinesins to the phragmoplast midline. The distinction between the localization of Rab-A2a and TIO seemed different in kin12 a/b (and f) lines.

We do not agree with this point; while the kinesin motility assay alone does not definitively show that they transport RAB-A2a vesicles, it is certainly supportive of that scenario when combined with the other existing evidence (RAB-A2a depletion in the double mutant, clear defects in cytokinesis and membrane mistargeting in the triple mutant, other recent work on Kin-12 in *Physcomitrium* etc). We are not entirely sure what the second point means, but both RAB-A2a and TIO exhibit a reduction in midzone intensity in the *kin-12a/b* mutants.

[10.1111/j.1365-313X.2012.05077.x identified the interaction domains between Kin12A/B and TIO, and tested their ability to restore cytokinesis, which may be discussed more in the

We agree that we had previously been too brief in presenting the information in this study. We have now added a new sentence in L655-657 to address this.

Regarding Supplementary Figure 1: More images or some quantification of the localization with the antibody would be helpful. How often does it localize to the phragmoplast midline? How does immunodepletion alter the localization? These data are likely already available to analyze.

We cannot easily quantify the immunlocalization data in the same manner as for the live images in this paper as they do not include an independent cell plate marker (which is the basis for quantification in the live images). Detailed discussion of the immunolocalization in the main manuscript would be of limited interest to most readers in an already dense paper, but we are happy to include it here. We observed 7 phragmoplasts in the standard immunolocalization experiments, all of which had Kin-12F signal at their midzone. The immunodepletion experiments removed the clear Kin-12F signal at the phragmoplast midzone, but in some cases a haze of signal remained in the area occupied by the phragmoplast. Importantly, treatment with pre-immune serum removed both signal from the phragmoplast midzone, as well as background signal that does occur with the Kin-12F antibody. Treatment of *kin-12f* SALK lines with the Kin-12F antibody resulted in retained background signal but specific loss of signal from the phragmoplast midzone.

What are the guides used, and how specific are they for Kinesin-12F or TIO? A diagram would clarify this.

Schematics and tables describing the guides used have been added to Figure S3 and Figure S10. CHOPCHOP predicts 0 off-targets for all of the sgRNAs, based on detecting up to 3 mismatches up to 20bp upstream of the PAM sequence. This has been explained in the legends of Fig S3 and Fig S10.

L 110: The Y2H data shown suggests that Rab-A2a binds variants with reduced nucleotide affinity, as well as unaltered Rab-A2a, and constitutively active Rab-A2a. Perhaps rephrase to "Y2H interactions suggest that either GTP-bound Rab-A2a or Rab-A2a lacking nucleotide bind Kin-12F."

This has been rephrased in L105-113, but not in the way suggested as that could imply that these variants substantially exist in plant cells, which is almost certainly untrue for RAB-A2a[NI].

Figure 5G-J: These experiments use r-BiFC in transient tobacco infiltration. As described, one cropped image each was used to demonstrate that Rab-A2a [WT] and [QL] increased Kin12A and TIO interacting puncta? Perhaps I missed something, but it was unclear from both the figure and the related SF8 and materials and methods how many times this experiment was repeated, how many cells were analyzed per independent experiment (for example: is it 31 cells or is that the number of puncta? Was the experiment repeated more than one time?). Please provide further information.

The 31 and other numbers in the legend of this figure refer to number of cells, not the number of punctae. In the initial submission, the rBiFC had been conducted 3 times independently, and an additional 3 experiments have now been conducted for the revision including the new combinations (see below). The figure legend for this and SF8 have been updated to clarify the number of cells and number of repeats. All experiments in this manuscript were done at least twice independently, often more.

The conclusion that Rab-A2a enhances TIO and KIN12A binding seems very important for the speculative model. All of that hinging on a transient overexpression experiment in another organism in non dividing cells, where variability is frequently observed? Please discuss.

We agree and acknowledge that our model is not definitive. It provides a parsimonious explanation for the observations made here and in the past. We have and are continuing to explore *in vitro* options to test this model, particularly the TIO kinase binding enhancement by RAB-A2a, but these are in the scale of years and so are well beyond the scope of reasonable revisions. We would also like to stress that our model currently incorporates data based on *in planta* dependency of TIO on Kin-12A/B and RAB-A2a function for proper localization, rBiFC data, and AF3 predictions.

To provide further support we have performed additional experiments: We computationally identified amino acids predicted to stabilise the interacting interface of Kin-12A and RAB-A2a. We then substituted these key residues, and found that their loss abolished the Kin-12A and RAB-A2a interaction in Y2H (Fig 5I and S8D). We then incorporated these substituted variants of Kin-12A and RAB-A2a into our rBiFC system (Fig 5I and S8E). We found that combinations of these substituted Kin-12A and RAB-A2a variants produced attenuated YFP punctae number and YFP signal similar to the already-described dominant-negative point mutants. This additional approach combined with the now large number of independent experiments producing the same results lend further support to our conclusions. These new results are outlined in L482-495.

We have now included in our "open questions" section we have added "Can enhanced interaction of TIO kinase with Kin-12A upon binding of RAB-A2a be demonstrated *in vitro*?" (L680-681)

Figure 5K-O: Localization of TIO in kin-12a/b and with overexpression of Rab-A2a [SN] and [NI]. TIO localization is altered in kin-12a/b Without seeing the phragmoplast (vs. FM6-64 staining), the conclusion regarding the localization is not clear. As I understood, Rab-A2 [SN] and [NI] both inhibit cytokinesis. Whether patchy TIO localization depends on Rab-A2a or is altered in cells with cytokinetic failures seems unclear. Further, the MAP65-3 appeared patchy in SF9? L462-463.

We do not have a line where simultaneous imaging of TIO, MTs and FM4-64 combined with Rab-A2a [SN]/[NI] would be possible. Our analyses show that YFP-TIO signal is considerably more diffuse perpendicular to the midzone in *kin-12a/b* (measured over 2μm each side). We have also separately demonstrated separately in this study (Fig S4) that phragmoplast morphology is, in fact, not substantially altered in *kin-12a/b* mutants, including its diameter perpendicular to the midzone. The reviewer is correct that RAB-A2a[NI] does quite aggressively inhibit cytokinesis, but the effect of RAB-A2a[SN] on cytokinesis is much milder. This indicates that inhibition of RAB-A2a can be disruptive to TIO patterning without substantial parallel effects on cytokinesis.

With regard to MAP65-3, we do find this to be sometimes patchy at the midzone even in wild-type backgrounds (examples of this can also be found in the literature: see Steiner et al., 2016 Plant J Fig 4, and Deng et al., 2024 Nature Comms Fig 5). The point we make with our quantification here is that aspects of MAP65-3 distribution and intensity do not differ upon expression of RAB-A2a, unlike YFP-TIO.

SF10: This figure is used to show that Rab-A2a does not accumulate in tio knockdowns, but it was unclear whether there was a phragmoplast (or even a cell plate?) at the stages shown. Since the authors have the materials in hand, this could be examined more closely.

We have now gathered further images of YFP:RAB-A2a in the *pGL2>>tio* mutant, and provide further elaboration here. We do indeed observe instances where no RAB-A2a/FM4 midzone accumulation is seen in *pGL2>>tio* cells undergoing cytokinesis (as visualized by nuclear disintegration and re-formation) when imaged at 5 min intervals. However, we have now also captured divisions where a RAB-A2a/FM4 cell plate began to form, but failed to produce a later cell plate or crosswall. Our interpretation of this is that the early stages of cell plate formation and RAB-A2a/FM4 accumulation can begin occur in *pGL2>>tio* cells during the phragmoplast initials phase (which remains in *pGL2>>tio* cells) but that subsequent phragmoplast dissolution prevents it from progressing beyond this. This indicates that TIO kinase is not *a priori* required for RAB-A2a accumulation at the midzone, which also lends further support to the directionality proposed in our final model. We have now included this data in Figure 6I and S10, and refer to it in L554-560.

Minor comments:

Typos in L 847

This has been corrected.

Referee #2:

The GTPase RABA2a is known for its association with developing cell plate in Arabidopsis. The results summarized in this manuscript provide direct evidence showing that three homologous Kinesin-12 motors probably are its effectors. Because published results by others suggested interaction between Kin12A/B with the

Fused kinase TIO, the authors reached a model of RABA2a-dependent recruitment of TIO to the phragmoplast midzone via Kin-12 motors. The work also supports a regulatory mechanism that links membrane trafficking and cytoskeletal organization during rapid expansion of the phragmoplast.

This comprehensive study answers a long-standing question regarding how RABA2a regulates cytokinesis. It also brings new insights into how cell plate-targeted membrane trafficking is tied to microtubule reorganization during rapid expansion of the phragmoplast. The authors also introduced experimental approaches that allowed conditional inactivation of essential genes in Arabidopsis. This work serves as a reference for future studies of other RAB proteins for their potential functions in the phragmoplast.

The authors are invited to provide explanations of the following points:
1. A very challenging matter related to the Arabidopsis RAB proteins is that multiple forms of RABA and perhaps RABE have been detected in the phragmoplast midzone. Although some of the published results may be questioned due to ectopic expression, it would be helpful if the authors could inform the readers whether the relationship between RABA2a and Kinesin-12 is reserved for RABA2a itself or beyond. The authors demonstrated that Kinesin-12A/B/F interacted with RABA2a but not RABA3, RABA5, and RABE by Y2H. Have other RABA2 and RABA1 been tested by similar assays? Perhaps single RABA2a mutant does not exhibit phenotypes in cell division or plant growth. Is it known whether RABA2 and other RAB genes function redundantly in cytokinesis?

It is often stated in the literature that Rab-A GTPase members within different clades are likely to act redundantly with each other, largely based on the fact that individual insertion mutants of Rab-A GTPases often have no or little phenotypic perturbation. In Chow et al., 2008 (Plant Cell), the authors localize all Rab-A2 and Rab-A3 members to the TGN/EE and cell plate, including demonstrating overlap of all individual Rab-A2 members in Arabidopsis at a TGN/EE domain with a heterologous-expressed *Pisum* Rab-A3 variant. They do not however definitively show that Rab-A2 members (4 in total) are functionally redundant with each other. Other Rab-A GTPases such as RAB-A5c has also been implicated in cytokinesis (Kirchhelle et al., 2016, Dev Cell), but we could not find any evidence that it interacts with Kin-12A/B/F.

We have not tested whether the other three Rab-A2 members interact with Kin-12A/B/F in Y2H, but suspect that they probably would. However, we do not believe that doing this at this stage would add much value, particularly as then we would then be obliged to follow up with other experiments *in planta* relating to the other three Rab-A2 paralogues in order to make a significant point.

2. The authors reached a very strong conclusion for Kinesin-12 function in membrane trafficking in the phragmoplast. This conclusion was largely based on the detected interaction between the motors and RABA2a. If the working model is correct as illustrated in Figure 7, one would expect the dependence of Kinesin-12 localization on RABA2a. The question is whether the authors have such evidence.

We do not understand this comment as we do not claim that RAB-A2a binding is required for Kin-12 localization to the midzone, nor does the working model require this. In fact, we showed in SF2 that inhibition of RAB-A2a function does not prevent Kin-12F from localizing to the midzone. This has perhaps been missed so this has now been moved to Fig 1H-J and referenced in L158-160. We propose in our model that Class II Kin-12s act to transport/position RAB-A2a to the midzone, which in turn directs TIO kinase recruitment to promote formation and expansion of the phragmoplast. This model does not necessitate nor imply a dependence of Class II Kin-12 localization on RAB-A2a.

3. It was intriguing that Kinesin-12F behaved differently from Kinesin-12A/B in the in vitro tests (Figure 4). Based on the fluorescent images presented, there was no obvious localization difference between Kin-12A/B and Kin-12F. It was unclear how the authors reached the conclusion as shown in the Figure 7 model. In the meantime, it is well known that many factors may contribute to the outcome of in vitro motility assays of kinesins. Without further evidence in vivo, perhaps it is too early to conclude Kin-12F functions as a tethering factor for older membrane compartments in the phragmoplast midzone.

Our analyses of Kin-12F distribution and Kin-12A distribution relative to RAB-A2a during cytokinesis has revealed the subtle but consistent difference that Kin-12F is depleted at the leading edge of the cell plate where vesicles are delivered, compared to the transition/lagging zone where fusion into a cell plate occurs. By contrast, Kin-12A is present in the leading edge. This was perhaps not clear in the previously submitted version as this data was spread across different figures (Fig 1 vs. Fig 4 vs. SF5). We have now centralized and expanded this data in Fig 4B-D so that this is easier to understand.

We accept that we do not definitively show that Kin-12F is a tether for older membranes at the lagging zone, but this interpretation does fit with our observations, including that Kin-12F does not depend on MAP65-3 for localization, and so we present it as a possibility in our speculative model. We do believe that Kin-12F is likely to be immobile; it shows no signs of motility in the gliding nor single molecule assays, lacks a complete LASGE

motif, and the PY motif conserved in Kin-12A/B that has been implicated in control of motility in other kinesins (e.g., Ganguly et al., 2018 Dev Cell). Our observation that YFP: Kin-12F rescues the phragmoplast expansion defect of *kin-12f* but not of *kin-12a/b* (Fig 4) also aligns with functional difference between them. We now indicate the lack of PY motif in L343-344 and Fig 3.

4. If Kin-12F functions differently from Kin-12A/B, one would expect that the mutants behave differently although all contribute to phragmoplast expansion. It would be helpful for the authors to explain why the Kin-12F association with older membrane compartments contributes to the expansion-why step 3 is critical for steps 4 and 5 in Figure 7A.

We agree that the exact function of Kin-12F is one of the key remaining questions from our study. As to why its loss impacts phragmoplast expansion, there are several possibilities. We now show that TIO patterning is not disrupted in the *kin-12f* mutant as it is in the *kin-12a/b* mutant (SF9A-C), so this is unlikely to be a causative factor. One possibility is that Kin-12F plays a TIO-independent role in microtubule organization at the lagging zone, in addition to binding RAB-A2a membranes, and that is required for proper phragmoplast expansion rates. Another is that Kin-12F may still be a target of TIO kinase activity (despite not having a TIO binding site), or a target of another factor that is downstream of TIO kinase. Following up on this by identifying the molecular targets of TIO kinase is our current priority. We now elaborate further on these possibilities in L671-675.

5. It was very interesting that Kin-12A/B but not Kin-12F interact with the TIO kinase. The authors also concluded that TIO functioned upstream of phragmoplast formation. This conclusion seems contradictory to what is proposed in the model which showed the Kin-12 function in recruitment of TIO. Indeed, the tio mutant showed serious defects in phragmoplast formation (Figure 6). If the phragmoplast is not properly formed, it probably would not undergo centrifugal expansion.

We do not claim that Kin-12F plays a role in TIO kinase recruitment/patterning at the midzone, only Kin-12A/B. We now provide additional evidence that Kin-12F does not play a role in patterning TIO kinase at the midzone by showing that YFP-TIO distribution is not disrupted in the *kin-12f* background, unlike in the *kin-12a/b* background.

Our data in a pGL2>>*tio* line does indeed show that about 45% of cells in these lines fail to progress beyond beyond the phragmoplast initials stage. The remaining 55% have a slower overall rate of expansion, and sometimes defects in shape and early fragmentation (Fig 6K). Our interpretation of this is that TIO kinase, positioned at least in part at the midzone by Kin-12A/B, is upstream of a fundamental process in MT organization that is required for both the stable formation of phragmoplast beyond the phragmoplast initials stage (which noticeably remains in *pGL2>>tio* lines) and subsequent phragmoplast expansion. A large proportion of phragmoplasts therefore fail entirely after phragmoplast initials stage, the rest that do manage to form have other problems (some of the 55% that do form may have partial rather than complete suppression of TIO).

6. In the manuscript, different terms were used in describing various truncated derivatives of Kin-12, including tail, TBD, delta TBD, C-term, and delta C-term. It would have been helpful if there is a diagram illustrating these different constructs, not only limited to the labeling in figure 1D. Supplementary figure 7A and B are highly repetitive so that may be combined.

We have removed the terms “C-term” and “delta C-term” (see comment #9 below) and have combined S7A and B to reduce repetition.

7. The genome editing method described in the manuscript may be applicable to many other essential genes. Such a novelty may be emphasized if this is the first time that the method is introduced.

Tissue-specific CRISPR-Cas9 has been described at least in principle in *Arabidopsis* before (e.g., Decaestecker et al., 2019. Plant Cell). The promoters etc. described in our manuscript are part of a larger set produced by the lab of Yvon Jaillais, who plans to produce a technical paper in due course.

8. Supplementary figure 7 has a title of TIO localization but the contents are about MAP65-3 localization.

We think that this is intended to mean SF9, whose title has now been corrected.

9. The Y2H results in Figure 1B and Supplementary Figure 7D seem contradictory regarding Kin-12F interactions with RABA5c and RABA3 as well as RABA2aSN. Clarification perhaps is necessary.

Thank you for pointing this out. This data dates from a previous version of the manuscript that we intended to remove to reduce the crowding, but left behind erroneously. In short, truncated versions of the Kin-12F tail that lack the extreme C-terminus (but still have the intact A2a binding domain) exhibit more promiscuous Rab interactions in Y2H than the full length Kin-12F tail. It is not entirely clear why this is the case, and it does not happen for Kin-12A and Kin-12B (except for a very slight degree between Kin-12A and RAB-A3 QL). We intended

but forgot to remove this data before submission to EMBO J as it does not add anything to the current story, and have now done so.

10. In Supplementary Figure 8, no obvious interaction between Kin-12F and TIO was detected by rBiFC when different RABA2a forms were expressed. Were similar rBiFC tests done for RABA2a and Kin-12?

We did not test in rBiFC the interaction between RAB-A2a and Kin-12 as we already include very strong evidence for this in the form of Y2H, *in planta* co-IP and *in planta* mis-localization work.

Referee #3:

The motor protein family kinesin-12 (KIN12) is crucial for cell division in plants. The family is divided into two classes that overlap and differ in their localization patterns and functions. Until now, the functions of class II kinesin-12 have been associated with phragmoplast organization in *Arabidopsis*, but a recent report on kinesin-12-II members in Physcomitrium suggests involvement in vesicle transport to the cell plate. Kirchelle's research group is investigating the function of class II KIN12F and also reports a role in vesicle transport, confirming the findings from moss.

Working on members of the KIN12 family is challenging due to multiple functional redundancies and the potential lethality of knockout mutants. The authors circumvent these problems by using tissue-specific knockdown and rescue experiments. They found distinct localizations and functions for KIN12A/B and KIN12F during cytokinesis, and their simultaneous knockout resulted in additive effects. Kin12ab/f triple mutants are stunted and exhibit cell wall stubs, branched cell walls, and multinucleated cells-all characteristics of severe cytokinesis defects known from mutants impaired in phragmoplast organization or vesicle fusion. While KIN12A/B are localized in the anterior zone of the phragmoplast, KIN12F remains in the posterior zone of the phragmoplast. Consistent with its localization, KIN12F does not interact genetically with MAP65-3, as its localization remains unchanged in *map65-3* mutants. All three class II KIN12s interact with the small GTPase RabA2a, which is involved in vesicle transport. In addition, TIO kinase interacts with KIN12A/B, but not with KIN12F. Conditional knockout of TIO kinase under the GLABRA2 promoter induced cytokinesis defects comparable to those observed previously, including membrane mis-targeting.

The study provides a molecular framework involving several interaction partners in which KIN12F and its close orthologs KIN12A and KIN 12B function. Based on these results, an improved view on how phragmoplast organization and expansion are coupled with vesicle delivery and cell plate formation emerges and clarifies how feedback signaling between microtubule dynamics and cell plate formation might be mediated by a TIO kinase - KIN 12A/B -RabA2a- KIN 12F module. The authors proposed a plausible model based on their findings. RabA2a delivery depends on KIN12A/B. KIN12A/B recruits TIO kinase and RabA2a is required for its turnover at the phragmoplast. Non-motile-KIN12F provides a platform for RabA2a vesicles for cell plate formation. This report is a major advancement towards our understanding on the long-standing question of how phragmoplast organization and cell plate expansion are linked.

I find the report very thorough; it adds new findings, repeats and confirms earlier findings, and summarizes them in an attractive model.

Only the function of KIN12 is still somewhat unclear to me. The authors suggest that KIN12F forms a platform for vesicle fusion and reason that this is due to the incomplete ATP hydrolysis motif. Can KIN12F be converted into a mobile kinesin by replacing the incomplete ATP hydrolysis motif with one from KIN12A or KIN12B?

This would indeed be very interesting to see, particularly if combined with a full head domain swap between Kin-12A and Kin-12F. We unfortunately do not have the constructs that this would require, and to give the most informative outcome this would also require *in planta* testing to see if these substitutions could shift the difference we observe between Kin-12A and Kin-12F localization at the midzone. We therefore think doing this would be beyond reasonable revisions at this stage, but it is an idea we wish that we had thought of sooner!

We agree that the one of the remaining questions regarding our work is what exactly Kin-12F does at the molecular level. Our current follow-up priority is to establish whether it is a target of TIO kinase activity or a TIO-dependent factor despite not appearing to bind to it, as this could be the missing link. L671-675 now elaborate further on this.

1st Revision - Editorial Decision 27th Mar 2026

Dear Charlotte,

Thank you for submitting the revised version of your manuscript to The EMBO Journal. The study has now been seen by two of the original referees, who find that their main concerns have been addressed satisfactorily and recommend acceptance of the manuscript after minor textual changes as requested by reviewer #1.

There are also a few editorial and formatting points that need to be addressed before I can extend official acceptance of the manuscript:

1. As part of the EMBO Press transparent editorial process, The EMBO Journal will publish online a Peer Review File to accompany accepted manuscripts. This file will be published in conjunction with your paper and will include the anonymous referee reports, your point-by-point response and all pertinent correspondence relating to the manuscript, including decision letters. Please note that the Author Checklist will be published at the end of the Peer Review File.
Please let us know if you want to remove or not any figures or data from the Peer Review File prior to publication. Please note that retaining unpublished data in the Peer Review File means that these count as published and that the Peer Review File would need to be referenced in future publications.
2. The manuscript sections should be in the following order: Title page - Abstract - Introduction - Results - Discussion - Methods - Data Availability - Acknowledgments - Disclosure and Competing Interests Statement - References - Figure Legends - (Main Tables with legends if applicable) - Expanded View Figure Legends.
3. Please upload the main and EV figures as individual production quality figure files in the .eps, .tif,or .jpg format (one file per figure).
4. Supplementary figures should either be compiled in a single Appendix prefaced with a brief table of contents including the page numbers, or turned in Expanded View (EV figures) ith nomenclature updated to Figure EV1-10. In such case, they should be uploaded as individual figure files and their legends assembled at the end of the manuscript after main figure legends.
5. Please include corresponding authors' email addresses on the title page of the manuscript.
6. CRediT has replaced the traditional author contributions section because it offers a systematic, machine-readable author contributions format that allows for more effective research assessment. Please remove the Authors Contributions from the manuscript and use the free text boxes beneath each contributing author's name in our online submission system to add specific details on the author's contribution. More information is available in our guide to authors.
7. Please rename "Competing interests" section into "Disclosure and competing interests statement".
8. Please rename the movies "Movie EV1" - "Movie EV2" and zip each movie file together with its corresponding legend.
9. Figure panels for Fig 6J is not mentioned in the manuscript text; please add the corresponding callouts.
10. In the line 565 there is a callout to "Figure J-L", please check and correct.
11. Please rename "Materials and Methods" section into "Methods".
12. Please update references according to The EMBO Journal style - where there are more than 10 authors on a paper, the first 10 should be listed, followed by 'et al.' Please remove DOIs from references to published articles, they should only be used for preprints and datasets that have not been published yet.
13. Please acknowledge BioRender use in a dedicated section in the Methods section using the following format:
Graphics:
(some of the... OR Figure #... OR synopsis) Graphics were created with BioRender.com.
14. We require a "Data Availability" section at the end of "Methods". As far as I can see, no data deposition in external databases is needed for this paper. If I am correct, then please state in this section: "This study includes no data deposited in external repositories". Further information can be found at https://link.springer.com/partners/embo-press/editorial-policies#Data%20availability%20statement.
15. Please add the information currently listed in the Primer Table in the Reagents and Tools table, Oligonucleotides section.
16. Please provide source data for the main manuscript figures as requested on October 7th, 2025.
17. Our data editors have flagged the following issues in figure legends that need correcting:
- Please provide the exact p values are not provided in the legends of figures 2Q, R; 3H, 5H, I, O, Q; 6J.
- Please define the box plots in terms of minima, maxima, centre, bounds of box and whiskers, and percentile in the legends of figures 2K, Q, R; 3H, P; 5H, I, Q.
- Please provide the information on the number and nature of replicates in the legends of figures 3P, 5Q.
18. Papers published in The EMBO Journal are accompanied online by a 'Synopsis' to enhance discoverability of the manuscript. It consists of A) a short (1-2 sentences) summary of the findings and their significance, B) 3-4 bullet points highlighting key results and C) a synopsis image that is 550x300-600 pixels large (width x height, jpeg or png format). You can either show a model or key data in the synopsis image. Please note that the image size is rather small and that text needs to be readable at the final size. Please send us this information together with the revised manuscript.

Please let me know if you have any questions regarding any of these points. You can use the link below to upload the revised files.

Thank you again for giving us the chance to consider your manuscript for The EMBO Journal. I look forward to receiving the final version.

With best wishes,

Ieva

Ieva Gailite, PhD
Senior Scientific Editor
The EMBO Journal
Meyerhofstrasse 1
D-69117 Heidelberg
Tel: +4962218891309
i.gailite@embojournal.org

We realize that it is difficult to revise to a specific deadline. In the interest of protecting the conceptual advance provided by the work, we recommend a revision within 3 months (25th Jun 2026). Please discuss the revision progress ahead of this time with the editor if you require more time to complete the revisions. Use the link below to submit your revision:

https://emboj.msubmit.net/cgi-bin/main.plex

Referee #1:

The authors are to be congratulated for addressing most reviewer comments very nicely and writing a fascinating paper. The remaining question I have is: how does Kin12F, which is non motile, recruit RABA2a to the forming cell plate? This seems to be the conclusion from Figure 3G. Some potential mechanisms should be discussed in the discussion section.

L185: tripe should be triple

Referee #2:

In the previous review, the authors were asked to provide clarifications regarding how their results may support the working model depicted in Figure 7. The revisions included further evidence showing the intriguing relationship among the RAB-A2a GTPase, three Kin-12 motors, and the fused kinase TIO1. The revision included, for example, how TIO1 localization was altered in the kin-12a/b mutant but not kin-12f. There is no question that the findings presented here advanced our knowledge regarding how these cytokinesis important proteins work together to drive the assembly and expansion of the phragmoplast. The story also inspires future experiments that could inform us how the seemingly immobile Kin-12F function redundantly as mobile Kin-12A/B and how the shared interaction with RAB-A2a could guide them to different domains in the phragmoplast midzone.

In short, the manuscript includes ample exciting findings so that is recommended for publication in this highly influential journal.

Dear Ieva,

We thank you and the reviewers for your revision response.

In response to the request from reviewer 1 for further details on how non-motile Kin-12F may recruit RAB-A2a to the forming cell plate we have added the following amended sentence to the discussion:

L441-448: This localisation is also consistent with the apparent lack of motile behaviour in Kin-12F, and we propose that it may primarily act to stabilise membrane-microtubule interactions during the formation of the cell plate tubulo-vesicular network, after the initial vesicle delivery to the phragmoplast leading edge. In this scenario, Kin-12F may attach from lagging edge zone microtubule plus ends to RAB-A2a positive membranes that have already transitioned from delivered vesicles into larger compartments, keeping these in position while they fuse into a tubulo-vesicular network.

The typo pointed out by reviewer 1 has been corrected.

Bests and thanks,
Charlotte

2nd Revision - Editorial Decision 29th Apr 2026

Dear Charlotte and Liam,

Thank you for addressing the final editorial and formatting points. I am now pleased to inform you that your manuscript has been accepted for publication in the EMBO Journal. Congratulations with a nice study!

Your manuscript will be processed for publication by EMBO Press. It will be copy edited and you will receive page proofs prior to publication. Please note that you will be contacted by Springer Nature Author Services to complete licensing and payment information.

You may qualify for financial assistance for your publication charges - either via a Springer Nature fully open access agreement or an EMBO initiative. Check your eligibility: https://link.springer.com/journal/44318/how-to-publish-with-us

Should you be planning a Press Release on your article, please get in contact with embo_production@springernature.com as early as possible in order to coordinate publication and release dates.

If you have any questions, please do not hesitate to contact the Editorial Office or me directly. Thank you for your contribution to The EMBO Journal, and I look forward to seeing your article online!

With best wishes,

Ieva

Ieva Gailite, PhD
Senior Scientific Editor
The EMBO Journal
Meyerhofstrasse 1
D-69117 Heidelberg
Tel: +4962218891309
i.gailite@embojournal.org

*****
Please note that it is The EMBO Journal policy for the transcript of the editorial process (containing referee reports and your response letters) to be published as an online supplement to each paper. If you should prefer removal of any referee-only figures included in the point-by-point response(s), e.g. because they may still be used for future publication or because they have been reproduced from published work by others, please do let us know immediately via response email.
More information is available here: https://link.springer.com/partners/embo-press/editorial-policies#Peer%20review

** Click here to be directed to your login page: https://emboj.msubmit.net

EMBO Press Author Checklist

Corresponding Author Name: Charlotte Kirchhelle	
Journal Submitted to: The EMBO Journal	
Manuscript Number: EMBOJ-2025-122269R	

USEFUL LINKS FOR COMPLETING THIS FORM
The EMBO Journal - Author Guidelines
EMBO Reports - Author Guidelines
Molecular Systems Biology - Author Guidelines
EMBO Molecular Medicine - Author Guidelines

Reporting Checklist for Life Science Articles (updated January
This checklist is adapted from Materials Design Analysis Reporting (MDAR) Checklist for Authors. MDAR establishes a minimum set of requirements in transparent reporting in the life sciences (see Statement of Task: 10.31222/osf.io/9sm4x). Please follow the journal's guidelines in preparing your
Please note that a copy of this checklist will be published alongside your article.

Abridged guidelines for figures
1. Data
The data shown in figures should satisfy the following conditions:
- the data were obtained and processed according to the field's best practice and are presented to reflect the results of the experiments in an accurate and unbiased manner.
- ideally, figure panels should include only measurements that are directly comparable to each other and obtained with the same assay.
- plots include clearly labeled error bars for independent experiments and sample sizes. Unless justified, error bars should not be shown for technical
- if n<5, the individual data points from each experiment should be plotted. Any statistical test employed should be justified.
- Source Data should be included to report the data underlying figures according to the guidelines set out in the authorship guidelines on Data

2. Captions
Each figure caption should contain the following information, for each panel where they are relevant:
- a specification of the experimental system investigated (eg cell line, species name).
- the assay(s) and method(s) used to carry out the reported observations and measurements.
- an explicit mention of the biological and chemical entity(ies) that are being measured.
- an explicit mention of the biological and chemical entity(ies) that are altered/varied/perturbed in a controlled manner.
- the exact sample size (n) for each experimental group/condition, given as a number, not a range;
- a description of the sample collection allowing the reader to understand whether the samples represent technical or biological replicates (including how many animals, litters, cultures, etc.).
- a statement of how many times the experiment shown was independently replicated in the laboratory.
- definitions of statistical methods and measures:
  - common tests, such as t-test (please specify whether paired vs. unpaired), simple χ2 tests, Wilcoxon and Mann-Whitney tests, can be unambiguously identified by name only, but more complex techniques should be described in the methods section;
  - are tests one-sided or two-sided?
  - are there adjustments for multiple comparisons?
  - exact statistical test results, e.g., P values = x but not P values < x;
  - definition of 'center values' as median or average;
  - definition of error bars as s.d. or s.e.m.

Please complete ALL of the questions below.
Select "Not Applicable" only when the requested information is not relevant for your study.

Materials

**Newly Created Materials**	**Information included in the manuscript?**	**In which section is the information available?** (Reagents and Tools Table, Materials and Methods, Figures, Data Availability Section)
New materials and reagents need to be available; do any restrictions apply?	Yes	Reagents and tools table, Material and Methods

**Antibodies**	**Information included in the manuscript?**	**In which section is the information available?** (Reagents and Tools Table, Materials and Methods, Figures, Data Availability Section)
For **antibodies** provide the following information:<br>- Commercial antibodies: RRID (if possible) or supplier name, catalogue number and or/clone number<br>- Non-commercial: RRID or citation	Yes	reagents and tools table

**DNA and RNA sequences**	**Information included in the manuscript?**	**In which section is the information available?** (Reagents and Tools Table, Materials and Methods, Figures, Data Availability Section)
**Short novel DNA or RNA including primers, probes**: provide the sequences.	Yes	material and methods

**Cell materials**	**Information included in the manuscript?**	**In which section is the information available?** (Reagents and Tools Table, Materials and Methods, Figures, Data Availability Section)
**Cell lines:** Provide species information, strain. Provide accession number in repository **OR** supplier name, catalog number, clone number, and/**OR** RRID.	Not Applicable	
**Primary cultures:** Provide species, strain, sex of origin, genetic modification status.	Not Applicable	
Report if the cell lines were recently **authenticated** (e.g., by STR profiling) and tested for mycoplasma contamination.	Not Applicable	

**Experimental animals**	**Information included in the manuscript?**	**In which section is the information available?** (Reagents and Tools Table, Materials and Methods, Figures, Data Availability Section)
**Laboratory animals or Model organisms:** Provide species, strain, sex, age, genetic modification status. Provide accession number in repository **OR** supplier name, catalog number, clone number, **OR** RRID.	Not Applicable	
**Animal observed in or captured from the field:** Provide species, sex, and age where possible.	Not Applicable	
Please detail **housing and husbandry conditions**.	Not Applicable	

**Plants and microbes**	**Information included in the manuscript?**	**In which section is the information available?** (Reagents and Tools Table, Materials and Methods, Figures, Data Availability Section)
**Plants:** provide species and strain, ecotype and cultivar where relevant, unique accession number if available, and source (including location for collected wild specimens).	Yes	Material and methods
**Microbes:** provide species and strain, unique accession number if available, and source.	Yes	Material and methods

**Human research participants**	**Information included in the manuscript?**	**In which section is the information available?** (Reagents and Tools Table, Materials and Methods, Figures, Data Availability Section)
If collected and within the bounds of privacy constraints report on age, sex and gender or ethnicity for all study participants.	Not Applicable	

**Core facilities**	**Information included in the manuscript?**	**In which section is the information available?** (Reagents and Tools Table, Materials and Methods, Figures, Data Availability Section)
If your work benefited from core facilities, was their service mentioned in the acknowledgments section?	Not Applicable	

Design

Study protocol	Information included in the manuscript?	In which section is the information available? (Reagents and Tools Table, Materials and Methods, Figures, Data Availability Section)
If study protocol has been **pre-registered, provide DOI in the manuscript**. For clinical trials, provide the trial registration number **OR** cite DOI.	Not Applicable	
Report the **clinical trial registration number** (at ClinicalTrials.gov or equivalent), where applicable.	Not Applicable	

Laboratory protocol	Information included in the manuscript?	In which section is the information available? (Reagents and Tools Table, Materials and Methods, Figures, Data Availability Section)
Provide DOI OR other citation details if **external detailed step-by-step protocols** are available.	Not Applicable	

Experimental study design and statistics	Information included in the manuscript?	In which section is the information available? (Reagents and Tools Table, Materials and Methods, Figures, Data Availability Section)
Include a statement about **sample size** estimate even if no statistical methods were used.	Yes	Material and Methods/Figures
Were any steps taken to minimize the effects of subjective bias when allocating animals/samples to treatment (e.g. **randomization procedure**)? If yes, have they been described?	Not Applicable	
Include a statement about **blinding** even if no blinding was done.	Not Applicable	
Describe **inclusion/exclusion criteria** if samples or animals were excluded from the analysis. Were the criteria pre-established?<br><br>If sample or data points were omitted from analysis, report if this was due to attrition or intentional exclusion and provide justification.	Not Applicable	
For every figure, are **statistical tests** justified as appropriate? Do the data meet the assumptions of the tests (e.g., normal distribution)? Describe any methods used to assess it. Is there an estimate of variation within each group of data? Is the variance similar between the groups that are being statistically compared?	Yes	Material and Methods/Figures

Sample definition and in-laboratory replication	Information included in the manuscript?	In which section is the information available? (Reagents and Tools Table, Materials and Methods, Figures, Data Availability Section)
In the figure legends: state number of times the experiment was **replicated** in laboratory.	Yes	Material and Methods
In the figure legends: define whether data describe **technical or biological replicates**.	Yes	Figure legends

Ethics

Ethics	Information included in the manuscript?	In which section is the information available? (Reagents and Tools Table, Materials and Methods, Figures, Data Availability Section)
Studies involving **human participants**: State details of **authority granting ethics approval** (IRB or equivalent committee(s), provide reference number for approval.	Not Applicable	
Studies involving **human participants**: Include a statement confirming that **informed consent** was obtained from all subjects and that the experiments conformed to the principles set out in the WMA Declaration of Helsinki and the Department of Health and Human Services Belmont Report.	Not Applicable	
Studies involving **human participants:** For publication of **patient photos**, include a statement confirming that consent to publish was obtained.	Not Applicable	
Studies involving experimental **animals**: State details of **authority granting ethics approval** (IRB or equivalent committee(s), provide reference number for approval. Include a statement of compliance with ethical regulations.	Not Applicable	
Studies involving **specimen and field samples:** State if relevant **permits** obtained, provide details of authority approving study; if none were required, explain why.	Not Applicable	

Dual Use Research of Concern (DURC)	Information included in the manuscript?	In which section is the information available? (Reagents and Tools Table, Materials and Methods, Figures, Data Availability Section)
Could your study fall under dual use research restrictions? Please check biosecurity documents and list of **select agents and toxins** (CDC): https://www.selectagents.gov/sat/list.htm	Not Applicable	
If you used a select agent, is the security level of the lab appropriate and reported in the manuscript?	Not Applicable	
If a study is subject to dual use research of concern regulations, is the name of the **authority granting approval and reference number** for the regulatory approval provided in the manuscript?	Not Applicable	

Reporting

The MDAR framework recommends adoption of discipline-specific guidelines, established and endorsed through community initiatives. Journals have their own policy about requiring specific guidelines and recommendations to complement MDAR.

Adherence to community standards	Information included in the manuscript?	In which section is the information available? (Reagents and Tools Table, Materials and Methods, Figures, Data Availability Section)
State if relevant guidelines or checklists (e.g., **ICMJE, MIBBI, ARRIVE, PRISMA**) have been followed or provided.	Not Applicable	
For **tumor marker prognostic studies,** we recommend that you follow the **REMARK** reporting guidelines (see link list at top right). See author guidelines, under 'Reporting Guidelines'. Please confirm you have followed these guidelines.	Not Applicable	
For **phase II and III randomized controlled trials**, please refer to the **CONSORT** flow diagram (see link list at top right) and submit the CONSORT checklist (see link list at top right) with your submission. See author guidelines, under 'Reporting Guidelines'. Please confirm you have submitted this list.	Not Applicable	

Data Availability

Data availability	Information included in the manuscript?	In which section is the information available? (Reagents and Tools Table, Materials and Methods, Figures, Data Availability Section)
Have **primary datasets** been deposited according to the journal's guidelines (see 'Data Deposition' section) and the respective accession numbers provided in the Data Availability Section?	Yes	Data Availaability Section
Were **human clinical and genomic datasets** deposited in a public access-controlled repository in accordance to ethical obligations to the patients and to the applicable consent agreement?	Not Applicable	
Are **computational models** that are central and integral to a study available without restrictions in a machine-readable form? Were the relevant accession numbers or links provided?	Not Applicable	
If publicly available data were reused, provide the respective **data citations in the reference list**.	Not Applicable	
